# The genus *Pterostichus* in China II: the subgenus *Circinatus* Sciaky, a species revision and phylogeny (Carabidae, Pterostichini)

**DOI:** 10.3897/zookeys.536.5982

**Published:** 2015-11-16

**Authors:** Hongliang Shi, Hongbin Liang

**Affiliations:** 1 College of Forestry, Beijing Forestry University, Beijing 100081, China; 2 Key laboratory of Zoological Systematics and Evolution, Institute of Zoology, Chinese Academy of Sciences, Beijing 100101, China

**Keywords:** China, *Pterostichus*, key, new species, endophallus, systematics

## Abstract

All of the known species of the Chinese endemic subgenus *Pterostichus (Circinatus)* are revised, keyed, and illustrated. Eleven new species and one new subspecies are described: *Pterostichus adelphus* **sp. n.** (Sichuan: Meigu, N28.66°, E103.06°); *Pterostichus ailaoicus* **sp. n.** (Yunnan: Xinping, N23.94°, E101.50°); *Pterostichus camelus* **sp. n.** (Sichuan: Mianning, N28.97°, E102.16°); *Pterostichus dimorphus* **sp. n.** (Yunnan: Dayao, N26.08°, E101.03°); *Pterostichus maitreya* **sp. n.** (Guizhou: Fanjingshan, N27.90°, E108.70°); *Pterostichus miao* **sp. n.** (Guangxi: Maoershan, N25.87°, E110.41°); *Pterostichus tumulus* **sp. n.** (Guizhou: Fanjingshan, N27.90°, E108.70°); *Pterostichus wangjiani* **sp. n.** (Yunnan: Dongchuan, N26.08°, E102.87°); *Pterostichus yan* **sp. n.** (Hubei: Shennongjia, N31.47°, E110.39°); *Pterostichus yuxiaodongi* **sp. n.** (Sichuan: Wolong, N30.99°, E103.15°); *Pterostichus zhygealu* **sp. n.** (Sichuan: Meigu, N28.67°, E103.05°); and *Pterostichus cavazzutianus mianningensis* **subsp. n.** (Sichuan: Mianning, N28.97°, E102.16°). *Pterostichus cavazzutianus* is proposed as a replacement name for *Pterostichus cavazuttii* Allegro and Sciaky 2010, preoccupied by Pterostichus (Sinosteropus) barbarae cavazuttii Sciaky and Facchini 2003. A lectotype is designated for *Pterostichus baenningeri* Jedlička 1931. Two species, *Pterostichus schuelkei* Sciaky & Wrase and *Pterostichus wenxianensis* Allegro & Sciaky, are moved from the subgenus *Circinatus* to *Morphohaptoderus*. An infra-subgeneric taxonomy is proposed for the subgenus *Circinatus* with four species groups. The male endophallus characters for most species of *Circinatus* were well studied, with three types of endophallus defined. A phylogenetic analysis based on adult morphological characters confirmed the infra-subgeneric classification and clarified some of the relationships among species. Two main lineages within *Circinatus* were identified from the phylogenetic analyses. Three of the four species groups were monophyletic, whereas the fourth group was paraphyletic.

## Introduction

In 2011, we began conducting a comprehensive investigation and taxonomic study of the *Pterostichus* of China. Our first contribution to the Chinese *Pterostichus* was the publication two years ago on the endemic subgenus *Wraseiellus* (Shi et al. 2013). In this paper, we focus on the subgenus *Circinatus* of the genus *Pterostichus*, which is also endemic to China. Following an expedition in 2012, a review of *Circinatus* was planned to describe some new species of the subgenus that were discovered in the provinces of Sichuan, Yunnan and Guizhou. During the expedition, most of the eleven previously described species were also collected. In this paper, eleven new species and one new subspecies are described. We expect to discover more new species of *Circinatus* in the near future, since the study of the Chinese *Pterostichus* fauna is still in its early stages compared with its high diversity.

The taxonomic value of the everted endophallus of Carabidae has been recognized in recent decades and was early on applied in some works on the genus *Carabus* (e.g., Ishikawa 1978, Imura et al. 1998). In the genus *Pterostichus*, the endophallic characters were also of value for both higher systematics (Sasakawa and Kubota 2007) and separation of similar species (Sasakawa 2009). However, in the Chinese *Pterostichus*, the endophallus was very poorly studied but it is now known for most of the *Circinatus* species (16 of 22). Information on this structure improves our understanding of the Chinese *Pterostichus* fauna and also provides solid support for the species determinations and the relationships among species described in the present paper.

The primary purposes of this paper are to (1) describe 12 new taxa of *Circinatus*; (2) provide a key for species determinations in this subgenus; (3) describe and illustrate the endophallus of most known species of *Circinatus* and estimate the rate of evolution of the endophallus in this subgenus; and (4) conduct a primary analysis of the relationships among species and classify the subgenus *Circinatus* into species groups. For all of the new taxa, complete descriptions, illustrations, and distribution maps are provided, particularly with the addition of endophallic characters. The eleven previously described species are also treated with new locality records, illustrations, and supplemental descriptions on male and female genitalia. In the taxonomic sections, the species are arranged alphabetically.

## Material and methods

### Material

This work was based primarily on the examination of *Circinatus* specimens, which included the type material of all known species except for two. The majority of the material was from the collection of the Institute of Zoology, Chinese Academy of Science, Beijing, China (IZAS). The specimens examined from other collections are indicated with abbreviations.

CCCC Collection of Changchin Chen, Tianjin, China

CDW Collection of David Wrase, Berlin, Germany

CRS Collection of Riccardo Sciaky, Milano, Italy

MNHN Muséum National d’Histoire Naturelle, Paris, France

MSNM Museo Civico di Storia Naturale, Milano, Italy

NHMB Naturhistorisches Museum, Basel, Switzerland

NMPC Národní Muzeum Přírodovědecké Muzeum, Prague, Czech Republic

### Methods

The methods and terminology follow previous work (Shi et al. 2013), except for the following supplements.

The male endophallus was prepared by microinjection. The median lobe of the aedeagus was soaked in 10% KOH solution at room temperature for 8–20 hours and was then moved to 100% ethanol. The basal orifice of the aedeagus was injected with 100% ethanol with the microinjector until the endophallus was fully everted. The treated genitalia remained in 100% ethanol for studying and imaging and were then transferred into glycerol for permanent storage. The endophallus everting process was not always successful; therefore, for very rare species, we did not always study the endophallus.

The reconstruction of the phylogeny was performed using WIN-PAUP Version 4.0b10 software. The cladograms were created with FigTree Version 1.4.0. Character evolution was reconstructed with WinClada and NONA software. Trees were edited with Adobe Photoshop software. Parameters used in the analyses of the phylogeny are listed in related texts.

### Terminology

In the genus *Pterostichus*, four types of elytral microsculpture are defined and are all present in the subgenus *Circinatus*. The elytral microsculpture sometimes forms a fine reticular pattern, and when the long diameter of one mesh is less than twice the length of its short diameter, it is called **isodiametric** (Fig. 130) microsculpture. When the long diameter of one mesh is more than twice the length of its short diameter, it is **transverse** (Fig. 131) microsculpture. **Linear** (Fig. 132) microsculpture is arranged almost horizontally, forming reticulations with the long diameter approximately tenfold the length of the short diameter. **Granular** (Fig. 129) microsculpture is similar to isodiametric but with the meshes strongly convex and forming granules, with elytral surface looking somewhat dull. In the same specimen, sometimes, the isodiametric microsculpture is on the basal half of the elytron, whereas transverse microsculpture is near the apex. The cases of linear microsculpture in *Circinatus* are always very weak and sometimes indistinct. Linear microsculpture may be responsible for the iridescent hue of the elytron. In *Pterostichus*, granular microsculpture is present only in the females of some species, and therefore, the elytral surface luster is different between the sexes. This type of sexual dimorphism occurs in many other subgenera in China, including all species of *Wraseiellus* and certain species of *Morphohaptoderus*, *Sinoreophilus*, *Metallophilus*, and *Pseudohaptoderus*. In *Circinatus*, females with granular microsculpture are found only in one species, *Pterostichus dimorphus* sp. n.

The gonopore lobe is the terminal lobe with an apical opening, namely, the true “gonopore.” However, we failed to evert the gonopore lobe in some of the studied specimens (see Fig. 34 for a fully everted gonopore lobe, and see Fig. 35 for a folded gonopore lobe). Therefore, for a better comparison between different species, when using the term gonopore, we refer to the base of the gonopore lobe but not the apical opening. The endophallic lobes are named based on their location in a presumed ideal endophallus model for each species group. The length of the right paramere was measured from the inner articulation to the apex (Fig. 28C).

## Taxonomy

### Subgenus Circinatus

Taxon classificationAnimaliaColeopteraCarabidae

Sciaky, 1996

#### Subgeneric characters.

The subgenus *Circinatus* is defined as follows: body length between 9.3–16.5 mm; elytral length approx 0.6 times body length or more; dorsal side blackish, without metallic color, elytron sometimes with iridescent shine. Eye large and convex; two supraorbital setae present; third antennomere glabrous except apical setae; terminal palpomere tubiform in both sexes; penultimate labial palpomere without seta near apex; submentum with two setae on each side, outer one much shorter than inner one. Pronotum usually round; hind angle usually completely rounded, rarely weakly defined; posterior seta distant from hind angle or located near hind angle; basal foveal inner groove long, poorly defined; outer groove less than half length of inner one, or completely absent; region between inner and outer grooves sometimes concave, so that basal fovea is present as a simple depression. Elytron with basal setigerous pore present; scutellar stria present, complete or nearly so; third interval with two setigerous pores (rarely one or three as individual aberrations), all adjacent to second stria, anterior one at approx middle, the posterior one at approx posterior fourth; ninth interval with umbilical pore series discontinuous and sparse in middle; seventh interval with two umbilical pores near apex. Metepisternum short, its length subequal to width of anterior margin; males with secondary sexual structures on terminal or penultimate sternum, or without such structures on sterna. Mesofemur with two setae on posterior margin, with single short spine subapically; metacoxa with two setae; metatrochanter with one seta; first metatarsomere with distinct carina on outer surface, more or less shallower in second and third metatarsomeres; fifth tarsomeres usually glabrous beneath, rarely setose. Male genitalia with apical orifice placed on dorsal side of aedeagus or somewhat twisted to left side; right paramere straight, slightly elongate, ratio length / width 2.5–4, apex rounded or obtuse. Endophallus variable, located on dorsal or ventral side of aedeagus. Female genitalia with spermatheca markedly elongate, seminal canal and receptaculum differentiated; receptaculum capitate or clavate; spermathecal gland inserted near base of receptaculum. Stylomere II saber-shaped, elongate, strongly bent outwards; outer margin with two (rarely three) ensiform setae, inner margin with one (rarely two) ensiform seta; two short nematiform setae located in a furrow near apex (Fig. 138). Female sternum VIII with transparent region in middle, V-shaped, triangular or quadrate, apical margin with fine setae or spines; female tergum VIII with apical half evenly chitinized.

#### Comparison.

The majority of the species in the subgenus *Circinatus* (16 of 22 known species) have a rounded pronotal hind angle and the pronotal posterior seta is distant from the hind angle. In the Chinese fauna of *Pterostichus*, six subgenera have this type of pronotum: *Eosteropus* Tschitschérine, *Oreolyperus* Tschitschérine, *Circinatus* Sciaky, *Gutta* Wrase & Schmidt, *Tubuliphallus* Sciaky & Allegro, and *Sinosteropus* Sciaky. Two of these subgenera have distributions that are distant from the others: *Eosteropus* is widely distributed in the Palearctic realm, and *Oreolyperus* is restricted to middle Asia. The distributions of the other four subgenera are close in range in southern China.

In comparison with *Eosteropus* and *Gutta*, *Circinatus* differs in having the elytral third interval usually with two setigerous pores, all pores adjacent to the second stria (the third interval usually with three pores, the first one adjacent to the third stria in the other two subgenera). Compared to *Oreolyperus*, *Circinatus* differs in having the elytral basal pore present (elytral basal pore absent in *Oreolyperus*). Compared to *Tubuliphallus*, *Circinatus* differs in having the elytral length more than 0.6 times the body length, apical orifice of aedeagus opened to the dorsal or left side (in *Tubuliphallus*, elytral length approximately 0.5 times the body length, apical orifice of aedeagus opened to the apex). Compared to *Sinosteropus*, *Circinatus* differs in: body size large (9.5–16.5 mm); body form less convex and relatively slender; pronotal basal fovea with the outer area of the inner groove usually flat or concave; right paramere straight and only slightly elongate. In *Sinosteropus*, the body size is small (6–10 mm); body form strongly convex and relatively stout; pronotal basal fovea with the outer area of the inner groove convex; right paramere usually strongly elongate, apex more or less bent.

Some species of the subgenera *Morphohaptoderus* Tschitschérine and *Neohaptoderus* Tschitschérine may have similar rounded pronotal hind angles. However, in these two subgenera, the posterior seta of the pronotum is always located very close to the hind angle, which distinguishes these subgenera from most species of *Circinatus*. Some species of *Morphohaptoderus* have pronota shaped similarly to *Circinatus*, but have a different number of setae on the metacoxa and male genitalia (for details see discussion under *Pterostichus schuelkei*).

Some species of *Circinatus* have a weakly defined hind angle and a pronotal posterior seta close to the hind angle (e.g., *Pterostichus baenningeri*), and these species therefore might have been misplaced into the subgenus *Neohaptoderus*. However, these species of *Circinatus* differ from the species of *Neohaptoderus* in having the pronotal basal foveal inner groove shallower and relatively weakly defined, the outer groove obsolete, and the apical orifice of the aedeagus opening ventrally. In *Neohaptoderus*, the pronotal basal foveal inner groove is deeper and always well defined, the outer groove is typically distinct, and the apical orifice of the aedeagus does not open ventrally.

#### Distribution.

**Maps 1–3.**
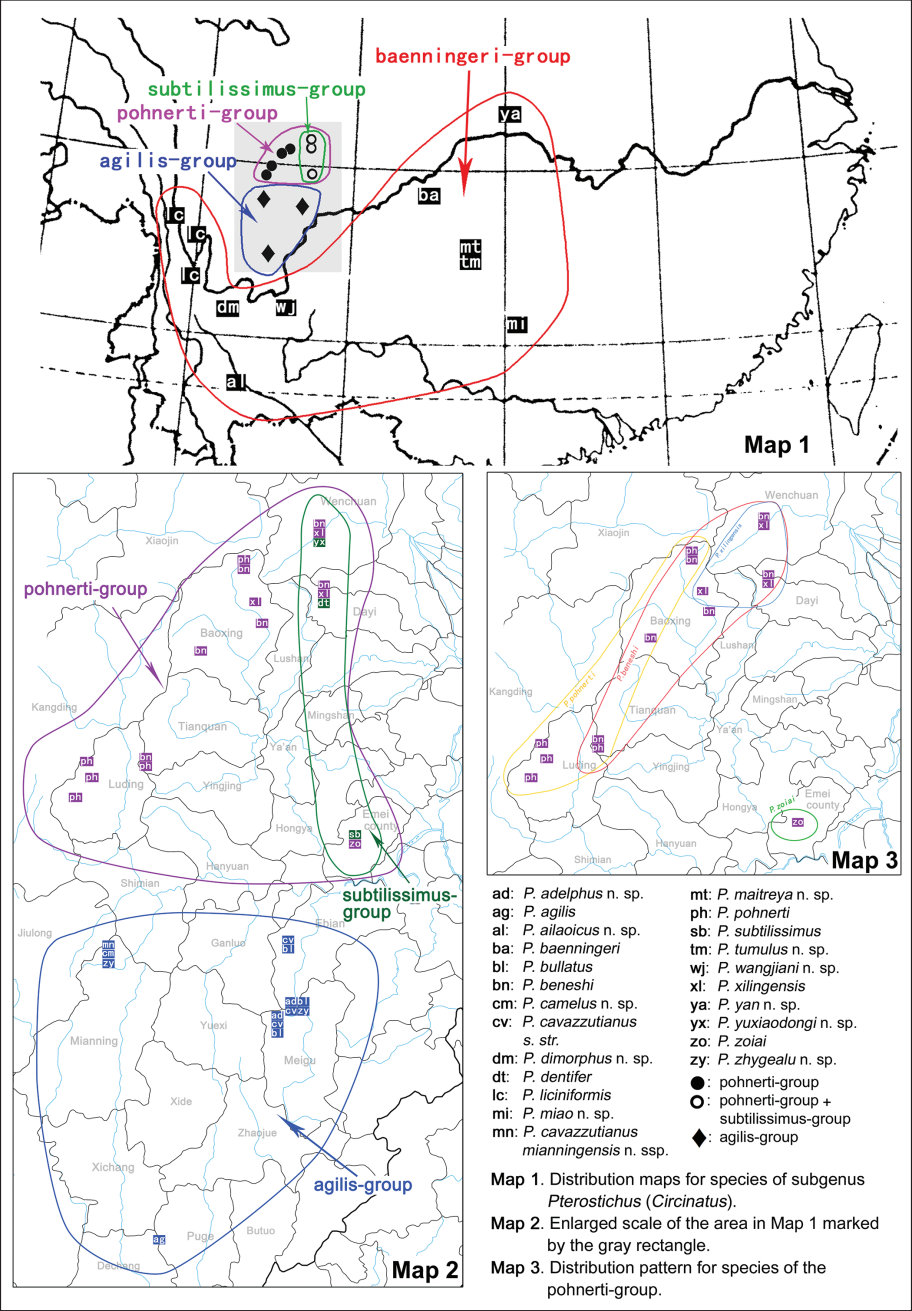


This subgenus includes 22 species and one subspecies, all endemic in China. The highest diversity is present in central-south Sichuan province (13 species), while other species are distributed in Yunnan (4 species), Guizhou (2 species), Chongqing (1 species), Guangxi (1 species) and Hubei (1 species) provinces (Maps 1, 2). All species are relatively narrowly distributed. Many of them were known only from one locality.

#### Literature.

In the present concept, the first species of *Circinatus* was described by Fairmaire (1888), although the original name *licinoides* is unavailable due to homonymy. Later, Jedlička described two more species (Jedlička 1931, 1934). However, in his time, these three species were not assigned to subgenera and were listed only as “*incertae sedis*” in his monograph (Jedlička 1962). Sciaky (1996) first established the subgenus *Circinatus*, and he added three new species. Later, one new species was described from Shaanxi (Sciaky and Wrase 1997). Allegro and Sciaky (2010) reviewed the subgenus *Circinatus*, described six new species and provided a key to all known species. Prior to the present work, a total of 13 species was included in the subgenus *Circinatus*, but two of these species actually do not belong in this subgenus.

#### Key to species of subgenus *Circinatus*

**Table:** 

1	Pronotum with two or more mid-lateral setae (Fig. 135); species from south Sichuan (Liangshan Yi Autonomous Prefecture)	**2**
–	Pronotum with only one mid-lateral seta (Fig. 136); species from other regions of China	**8**
2	Elytron with intervals interrupted, forming large bumps; Sichuan (Meigu, Ebian)	***Pterostichus bullatus* Allegro & Sciaky**
–	Elytron with normal intervals	**3**
3	Elytron with transverse or isodiametric microsculpture more or less distinct; male terminal sternum not modified, penultimate sternum modified (only barely tumid in *Pterostichus zhygealu*); apical lamella of aedeagus longer, more than one third length of the apical orifice	**4**
–	Elytron with very faint linear microsculpture, sometimes microsculpture invisible; male terminal sternum modified or not, penultimate sternum not modified; apical lamella of aedeagus shorter, approx one fourth length of the apical orifice	**6**
4	Fifth tarsomere setose beneath; males with two large tubercles on penultimate sternum; Sichuan (Mianning)	***Pterostichus camelus* sp. n.**
–	Fifth tarsomere glabrous beneath; male penultimate sternum without large tubercles, only slightly tumid on each side, middle region faintly depressed	**5**
5	Pronotal basal fovea with outer groove faintly defined, approx half length of the inner one, outer area of the inner groove distinctly concave; elytral humeral tooth indistinct, not pointed; apical lamella of aedeagus slightly hooked; larger species, body length 13.5–14.5 mm; Sichuan (Meigu, Ebian)	***Pterostichus zhygealu* sp. n.**
–	Pronotal basal fovea with outer groove completely vanished, outer area of the inner groove flat; elytral humeral tooth distinct, short and sharp, pointed backwards; apical lamella of aedeagus not hooked; smaller species, body length 10–12 mm; Sichuan (Luojishan)	***Pterostichus agilis* Allegro & Sciaky**
6	Pronotum round and wide (Fig. 4); basal fovea almost impunctate; elytral lateral margin wide and expanded; male terminal sternum not modified; apical lamella of aedeagus wide, located on the middle of aedeagus apex (Fig. 34B); Sichuan (Meigu, Ebian)	***Pterostichus adelphus* sp. n.**
–	Pronotum narrower (Fig. 5); basal fovea distinctly punctate; elytral lateral margin narrow and deep; males with terminal sternum somewhat depressed in the middle; apical lamella of aedeagus located on the right side of aedeagus apex (Fig. 35B)	**7**
7	Male with terminal sternum not bending downwards apically, slightly rugose subapically; apical lamella of aedeagus with length approx 0.8 times its basal width; Sichuan (Mianning)	***Pterostichus cavazzutianus mianningensis* ssp. n.**
–	Male with terminal sternum distinctly bending downwards apically, not rugose subapically; apical lamella of aedeagus with length approx same as its basal width; Sichuan (Meigu, Ebian)	***Pterostichus cavazzutianus* s. str. replacement name**
8	Pronotal posterior seta close to hind angle (Fig. 136), distance between seta and hind angle less than half of the distance between hind angle and inner basal foveal groove; hind angle usually more or less distinct	**9**
–	Pronotal posterior seta distant from hind angle (as in Fig. 135), distance between seta and hind angle approx same as the distance between hind angle and inner basal foveal groove; hind angle usually completely rounded (except for *Pterostichus tumulus* sp. n.)	**14**
9	Elytron with distinct transverse or isodiametric microsculpture; males with terminal sternum strongly concave in the middle; outer area of the inner basal foveal groove slightly convex; Yunnan (Dongchuan)	***Pterostichus wangjiani* sp. n.**
–	Elytron microsculpture usually indistinct, if present, then linear; males with two tubercles on terminal sternum; outer area of the inner basal foveal groove flat; species from other provinces of China	**10**
10	Tubercles on male terminal sternum large, approx one third length of the sternum; smaller species, body length 11–12 mm; Chongqing, Yunnan	**11**
–	Tubercles on male terminal sternum small or faint, less than a quarter length of the sternum; larger species, body length 12.5–14 mm; Hubei, Guizhou, Guangxi	**12**
11	Fifth tarsomeres setose beneath; tubercles on male terminal sternum distant from each other, distance between tubercles greater than the distance between setae on penultimate sternum; apical lamella of aedeagus approx same length as its basal width; Chongqing (Jinfoshan)	***Pterostichus baenningeri* Jedlička**
–	Fifth tarsomeres glabrous beneath; tubercles on male terminal sternum close to each other, distance between tubercles less than the distance between setae on penultimate sternum; apical lamella of aedeagus approx two times as long as its basal width; Yunnan (Ailaoshan)	***Pterostichus ailaoicus* sp. n.**
12	Pronotal lateral margin slightly sinuate before hind angle; elytral shoulder angle (formed by basal ridge and lateral margin) almost rounded; apical lamella of aedeagus distinctly longer than basal width, without groove dorsally; Guangxi (Maoershan)	***Pterostichus miao* sp. n.**
–	Pronotal lateral margin not sinuate before hind angle; elytral shoulder forming obtuse angle with the basal ridge and lateral margin; apical lamella of aedeagus almost same length as the basal width, base of apical lamella grooved dorsally	**13**
13	Pronotal hind angle rounded; tubercles on male terminal sternum faint; apical lamella of aedeagus gradually narrowed to apex (Fig. 31B); Hubei (Shennongjia)	***Pterostichus yan* sp. n.**
–	Pronotal hind angle forming indistinct obtuse angle; tubercles on male terminal sternum very small but distinct; apical lamella of aedeagus rounded, not narrowed to apex (Fig. 41B); Guizhou (Fanjingshan)	***Pterostichus maitreya* sp. n.**
14	Pronotal hind angle forming obtuse angle; male terminal sternum with an elongated tubercle at the middle; Guizhou (Fanjingshan)	***Pterostichus tumulus* sp. n.**
–	Pronotal hind angle completely rounded; male terminal sternum without middle tubercle	**15**
15	Male terminal sternum strongly depressed in the middle; elytral shoulder angle (between basal ridge and lateral margin) completely rounded OR females with granular elytral microsculpture; species from Yunnan	**16**
–	Male terminal sternum not modified; elytral shoulder angle forming distinct angle; females with normal elytral microsculpture, same as male; species from Sichuan	**17**
16	Elytral microsculpture granular in females, isodiametric as usual in males; elytral shoulder angle distinct; males with terminal sternum depressed in the middle, and bearing two sharp teeth pointing backward beside the depression; Yunnan (Dayao)	***Pterostichus dimorphus* sp. n.**
–	Both sexes with faint linear elytral microsculpture; elytral shoulder angle completely rounded; males with terminal sternum only depressed in the middle, without such teeth; Yunnan (Lijiang, Shangri-La, Deqin)	***Pterostichus liciniformis* Csiki**
17	Elytron not iridescent, with transverse or isodiametric microsculpture, shallow but usually distinct; larger species, body length between 14–16 mm; apex of aedeagus strongly bent to right side	**18**
–	Elytron with distinctly iridescent shine, microsculpture linear, very faint or invisible; smaller species, body length between 10.0–12.5 mm; apex of aedeagus not or only slightly bent to right side	**20**
18	Pronotal basal foveal inner and outer grooves completely fused, basal fovea simple and deep (Fig. 108); apical lamella of aedeagus long and truncate (Fig. 46B); Sichuan (Wolong)	***Pterostichus yuxiaodongi* sp. n.**
–	Pronotal basal fovea with anterior part of outer groove distinctly separated from the inner groove, so that basal fovea is a little bifid anteriorly (Fig. 106); apical lamella of aedeagus very short and rounded (Fig. 33B)	**19**
19	Elytral basal ridge strongly oblique lateral-posteriorly, humeral tooth long and sharp, pointing lateral-posteriorly; apex of aedeagus simple, not constricted (Fig. 33B); Sichuan (Dayi)	***Pterostichus dentifer* Allegro & Sciaky**
–	Elytral basal ridge weakly oblique lateral-posteriorly, humeral tooth short and obtuse, pointing laterally; apex part of aedeagus strongly constricted in the middle part of apical orifice (Fig. 45B); Sichuan (Emei Mt.)	***Pterostichus subtilissimus* Sciaky**
20	Pronotal basal fovea with outer groove completely vanished, outer area of the inner groove flat; elytron without humeral tooth	**21**
–	Pronotal basal fovea with outer groove present, forming a short ridge near the hind angle; elytron usually with very small humeral tooth	**22**
21	Apical lamella of aedeagus approx 3/4 as long as basal width; endophallus extended dorsally; Sichuan (Wolong, Baoxing, Tianquan, Xilingxueshan)	***Pterostichus beneshi* Sciaky**
–	Apical lamella of aedeagus approx half as long as basal width; endophallus bent dorsal-basally; Sichuan (Emei Mt.)	***Pterostichus zoiai* Sciaky**
22	Pronotal basal fovea slightly punctate; apical lamella located on right side of aedeagal apex; endophallus bent apically; Sichuan (Jiulong, Luding, Tianquan, Baoxing)	***Pterostichus pohnerti* Jedlička**
–	Pronotal basal fovea usually impunctate; apical lamella located on the middle of aedeagal apex; endophallus bent dorsally; Sichuan (Xilingxueshan, Wolong, Baoxing)	***Pterostichus xilingensis* Allegro & Sciaky**

### New taxa descriptions

#### Pterostichus (Circinatus) adelphus sp. n.

Taxon classificationAnimaliaColeopteraCarabidae

http://zoobank.org/F2B0B40D-EB12-46B9-AD4C-EB09F9576B16

Chinese common name: 弟兄通缘步甲 (Dì Xiōng Tōng Yuán Bù Jiă)

Figures 4 , 34 , 53 , 71 , 90 , 135

##### Type locality.

Sichuan: Meigu County, Dafengding national reserve, Hongxi station (N28.65850° E103.06123°), altitude 2541 m.

##### Type material.

**Holotype** (IZAS): male, body length = 11.1 mm, pin mounted, genitalia dissected and glued on plastic film pinned under specimen, “CHINA, Sichuan Prov., Meigu / county, Dafengding nat. res., / Hongxi station, mixed forest; / N28.65850 E103.06123"; “2541 m, 2012.VI.13 day, under dead / log; SHI Hongliang & YANG / Ganyan lgt. Inst. Zool., CAS / 美姑县大风顶洪溪保护站”; “HOLOTYPE ♂/ Pterostichus (Circinatus) / adelphus new species / des. SHI H.L. 2015” [red label]. **Paratypes, a total of 3 males, 1 female**: 3 males, 1 female (IZAS): “CHINA, Sichuan Prov., Yizi pass btw. Meigu county and Ebian county, mixed forest; N28.67477 E103.05248, 2923 m; 2012.VI.15; by pit fall trap; SHI Hongliang & LIU Ye leg.”.

##### Diagnosis.

Pronotum with three or four mid-lateral setae; hind angle completely rounded; basal fovea almost impunctate; pronotum wide and round; elytral microsculpture linear; males with terminal sternum not modified; apical lamella located on middle of aedeagal apex, length approx same as basal width. This new species has the widest apical lamella among the species of the *agilis*-group.

This new species is sympatric with and very similar to *Pterostichus cavazzutianus* s. str. Besides the significant differences in male genitalia (apical lamella shape and endophallus, Figs 34, 35), the two species also differ in: (1) *Pterostichus adelphus* sp. n. with wider and rounder pronotum, body slightly stouter than *Pterostichus cavazzutianus* s. str.; (2) basal fovea almost impunctate in *Pterostichus adelphus* sp. n. but distinctly punctate in *Pterostichus cavazzutianus* s. str.; (3) elytral lateral expansion much wider in *Pterostichus adelphus* sp. n.; (4) male terminal sternum not modified in *Pterostichus adelphus* sp. n., but shallowly depressed in *Pterostichus cavazzutianus* s. str.

##### Description.

Body form relatively stout, body length 9.9–11.1 mm; dorsal side black, shining; elytron with faint iridescent shine; mouthparts, antenna and tarsus yellowish brown; ventral side almost black. Both sexes with similar elytral microsculpture, very faint and linear. **Head**. Frons without punctures; antenna reaching elytron basal fifth; gena approx same length as eye, briefly tumid behind eye. **Pronotum** round, widest before middle, PW/PL = 1.21–1.32; usually four (sometimes three) mid-lateral setae present, first one close to anterior angle, last one near middle of lateral margin, a little distant from other three; posterior seta far distant from hind angle, distance between seta and hind angle approx same as distance between hind angle and inner basal foveal groove; hind angle completely rounded; basal fovea shallow, faintly defined; inner groove subparallel to median line, slightly curved outwards; outer groove completely vanished, outer area of inner groove flat; basal foveal area usually impunctate, sometimes with very sparse punctures on inner area of inner groove. **Elytron** oviform, basal ridge slightly oblique; elytral shoulder slightly narrowed, basal ridge and lateral margin forming obtuse angle, humeral tooth indistinct; intervals feebly convex; striae moderately deep, without punctures; scutellar stria short but complete; third interval with two setigerous pores adjacent to second stria; umbilical pore series on ninth interval sparse in middle, composed of 15–16 pores (6, 1, 8–9). **Ventral side**. Proepisternum impunctate or slightly punctate near posterior margin; mesepisternum densely punctate; metepisternum impunctate; terminal or penultimate sternum of males not modified. **Legs**. Fifth tarsomeres glabrous beneath; males with apical half of mesotibia slightly widened, inner margin slightly crenulate; first metatarsomere with distinct carina on outer surface, such carina on second and third metatarsomeres superficial. **Male genitalia**. Median lobe of aedeagus bent approx 90 degrees, apex slightly bent ventrally (Fig. 34A); ventral margin straight in middle, dorsal margin gradually curved; apical orifice large, slightly turned to left side, not opened on ventral side; apical lamella short, approx one fourth length of apical orifice, laminate, not thickened; in dorsal view, apical lamella located at middle of aedeagal apex, approx triangular with rounded apex, wide and short, length approx equal to basal width (Fig. 34B). Right paramere straight and slender, length approx 3.5 times greatest width, apex rounded (Fig. 34C). **Endophallus** (Fig. 34D, E, F) short, bent to ventral side, across apical lamella, and then turned to aedeagal base; gonopore (**gp**) located at approx same level as apical lamella. Six distinct lobes recognized: ventral-basal lobe I (**vb-I**) very small, close to base of apical lamella, membranous with its upper surface scaled; ventral-basal lobe II (**vb-II**) large and strongly chitinized, arcuate, strongly pointed, its upper surface with a membranous structure connected with vb-III; ventral-basal lobe III (**vb-III**) small, rounded with apex pointed, located on left side of vb-II, evenly decorated with very fine scales; ventral-apical lobe (**va**) short and wide, almost covered by vb-II, heavily pigmented, decorated with coarse scales, located on right side of vb-II; pre-apical lobe (**pa**) narrow and long, tubiform, without decoration, located on right lateral side; right lobe (**rl**) large, much thicker and a little longer than pa in ventral view, apex truncate, evenly decorated with fine scales. **Female genitalia**. Spermatheca (Fig. 53) with seminal canal approx three times as long as receptaculum; receptaculum capitate, club approx half length of receptaculum; spermathecal gland slightly expanded; seminal canal inserted at base of common oviduct, base of seminal canal sclerotized. Stylomere II with two ensiform setae at outer margin and one at middle of inner margin, two very short nematiform setae located in furrow near the apex. Female sternum VIII (Fig. 71B) with dense and fine spines on posterior margin; posterior margin rounded, shallowly notched in middle; posterior region chitinized, anterior region semi-chitinized, middle transparent region V-shaped, not adjacent to posterior notch in middle. Female tergum VIII (Fig. 71A) with anterior third well chitinized, posterior two thirds semi-chitinized and pigmented with dense spots.

##### Distribution.

This species was known only from the border between Meigu and Ebian counties (Sichuan, Liangshan Yi Autonomous Prefecture). The two collecting localities are approx 2 km apart. (Map 2)

##### Etymology.

The name *adelphus* derives from the Greek epithet “*adelph*” which means brother, referring to the similarity of this new species to a sympatric species *Pterostichus cavazzutianus*.

##### Affinities.

This new species seems to be close to *Pterostichus cavazzutianus* in their similarities in the elytral microsculpture and short apical lamella of aedeagus.

##### Habitat.

This species was collected in mixed forest with dominant large pines, and rich in dead logs. Most specimens were collected by pitfall trap, and also one was found under or in dead logs. In Yizi pass, *Pterostichus adelphus* sp. n. was found together with other three *Circinatus* species (*Pterostichus bullatus*, *Pterostichus cavazzutianus* s. str., *Pterostichus zhygealu* sp. n.), but seems rarer than other species. This locality has the richest diversity of the subgenus *Circinatus*.

##### Variation.

Two paratypes have three setigerous pores on one or both elytra.

#### Pterostichus (Circinatus) ailaoicus sp. n.

Taxon classificationAnimaliaColeopteraCarabidae

http://zoobank.org/D7ECDAEE-BB43-4C4A-B668-875E6B2540F2

Chinese common name: 哀牢通缘步甲 (Āi Láo Tōng Yuán Bù Jiă)

Figures 11 , 29 , 97

##### Type locality.

Yunnan: Xinping County, Jinshan pass in Ailao mountain (= Ailaoshan) (N23.947°, E101.499°), altitude 2351 m.

##### Type material.

**Holotype** (IZAS): male, body length = 10.9 mm, board mounted, genitalia dissected and glued on plastic film pinned under specimen, “CHINA, Yunnan, Xinping / county, Jinshan pass in Ailao / mount range, 2010.IX.14 / YANG Xiaodong lgt. / B10y5006, C.C.C.C.”; “N23°56.849’ / E101°29.993’ 2351 m / daytime, mixed forest, in or on dead log”; “HOLOTYPE ♂/ Pterostichus (Circinatus) / ailaoicus new species / des. SHI H.L. 2015” [red label].

##### Diagnosis.

Pronotum with single mid-lateral seta; posterior seta located almost at hind angle; hind angle forming indistinct obtuse angle, lateral margin straight before hind angle; elytral microsculpture almost invisible; elytral shoulder angle distinct; males with two large tubercles on terminal sternum; tubercles close to each other, distance between tubercles less than distance between setae on penultimate sternum; fifth tarsomeres glabrous beneath. Apical lamella of aedeagus long, its length approx 2.1 times basal width, apex widened, with a small tooth on right margin. Comparisons between similar species are given in Table 1 under *Pterostichus maitreya* sp. n.

**Table 1.** Comparisons for *Pterostichus maitreya*, *Pterostichus baenningeri*, *Pterostichus yan*, *Pterostichus ailaoicus* and *Pterostichus miao*.

Characters / Species	*Pterostichus baenningeri*	*Pterostichus maitreya*	*Pterostichus miao*	*Pterostichus ailaoicus*	*Pterostichus yan*
fifth tarsomeres beneath	setose	glabrous	glabrous	glabrous	glabrous
pronotal hind angle	weakly angulate	weakly angulate	rounded	weakly angulate	rounded
pronotal lateral margin, before hind angle	straight	straight	sinuate	straight	straight
elytral shoulder angle	obtuse angle	obtuse angle	rounded	obtuse angle	obtuse angle
tubercles on terminal sternum	large	medium	small	large	small
apical lamella widened or narrowed to apex	neutral	widened	widened	widened	narrowed
apical lamella oriented left	yes	no	no	no	yes
apical lamella L/W	1.3	1.0	1.7	2.1	1.1
apical lamella dorsal side, whether grooved on base	grooved	grooved	ungrooved	ungrooved	grooved
locality	Chongqing	Guizhou	Guangxi	Yunnan	Hubei

This new species is similar to *Pterostichus baenningeri* in body size and form, pronotal shape, and male terminal sternum. Despite the significant differences in male genitalia (shape of apical lamella, see Figs 29B and 40B), they also differ in: (1) *Pterostichus ailaoicus* sp. n. have the fifth tarsomeres glabrous beneath, but these are setose in *Pterostichus baenningeri*; (2) *Pterostichus ailaoicus* sp. n. have the two tubercles on the male terminal sternum close to each other, distance between tubercles less than the distance between setae on penultimate sternum; but in *Pterostichus baenningeri*, distance between tubercles on terminal sternum is greater than the distance between setae on penultimate sternum.

##### Description.

Body form fairly elongate, body length 10.9 mm; dorsal side almost black, moderately shining, elytron with faint iridescent shine; mouthparts, antenna, tarsus, tibia, and apex of femur reddish brown; ventral side blackish. Elytral microsculpture weak, barely visible, linear. **Head**. Frons without punctures; antenna reaching elytron basal sixth; gena shorter than length of eye, briefly tumid behind eye. **Pronotum** round, lateral margin curved in middle, nearly straight before hind angle, widest at approx anterior two fifths; posterior margin a little narrower than anterior margin; PW/PL = 1.10; one mid-lateral seta present, located a little before greatest width; posterior seta close to but a little anterior to hind angle; hind angle forming indistinct obtuse angle; basal fovea shallow, faintly defined; inner groove subparallel to median line, not reaching posterior margin; outer groove completely vanished; outer area of inner groove flat; basal foveal area finely punctate along inner groove. **Elytron** oviform, with basal ridge almost straight; elytral shoulder moderately widened, shoulder angle between basal ridge and lateral margin forming obtuse angle, humeral tooth very small, not jutting out; intervals feebly convex; striae moderately deep, without punctures; scutellar stria short, apex free; third interval with two setigerous pores adjacent to second stria; umbilical pore series on ninth interval sparse in middle, composed of 16–17 pores (6, 1, 9–10). **Ventral side**. Proepisternum without punctures, mesepisternum and metepisternum finely punctate; penultimate sternum not modified; terminal sternum with two large tubercles vaguely defined, approx one third length of terminal sternum; tubercles located a little before middle of sternum, distance between tubercles less than distance between two setae on penultimate sternum; region between tubercles not depressed. **Legs**. Fifth tarsomeres glabrous beneath; males with apical half of mesotibia slightly widened, inner margin crenulate; first metatarsomere with distinct carina on outer surface, such carina superficial on full length of second metatarsomere. **Male genitalia**. Median lobe of male genitalia bent approx 90 degrees, apex gradually bent ventrally (Fig. 29A); ventral margin slightly curved in middle, weakly sinuate before apex; dorsal margin gradually curved; apical orifice large, slightly turned to left side, opened on ventral side; in lateral view, apical lamella very long, laminate with base slightly thickened, sinuate before apex, apex pointing apical-ventrally, its length approx one third length of apical orifice; in dorsal view, apical lamella located on right side of aedeagal apex, its base not grooved on dorsal surface; apical lamella long with rounded apex, length approx 2.1 times basal width, distinctly widened at approx apical third, with a small tooth on right margin (Fig. 29B). Right paramere straight and stout, apical half fusiform, slightly enlarged and then narrowed to apex, inner margin evenly curved before apex; length approx three times greatest width; apex obtuse (Fig. 29C). Endophallus not studied. Female genitalia unknown.

##### Distribution.

This species is known only from the holotype collected from Ailaoshan Mt. in Yunnan Province (Map 1). The altitude is 2351 m.

##### Etymology.

This new species is named for its type locality, Ailao Mountain.

##### Affinities.

*Pterostichus ailaoicus* sp. n. is close to *Pterostichus baenningeri*, *Pterostichus maitreya* sp. n., *Pterostichus miao* sp. n., and *Pterostichus yan* sp. n. in their similarities of male terminal sternum and male genitalia.

#### Pterostichus (Circinatus) camelus sp. n.

Taxon classificationAnimaliaColeopteraCarabidae

http://zoobank.org/FCF37F99-6EF6-40F3-89CF-0C032729356C

Chinese common name: 骆驼通缘步甲 (Luò tuóTōng Yuán Bù Jiă)

Figures 9 , 28 , 60 , 76 , 95 , 118

##### Type locality.

Sichuan: Mianning County, Yele reserve (N28.96508°, E102.16137°), altitude 2988 m.

##### Type material.

**Holotype** (IZAS): male, body length = 12.7 mm, pin mounted, genitalia dissected and glued on plastic film pinned under specimen, “CHINA, Sichuan, Mianning / county, Yele reserve, / 2988 m, mixed forest; / N28.96508 E102.16137"; “2012.VI.24, pit fall trap; SHI / Hongliang, YANG Ganyan & / LIU Ye lgt., Inst. Zool., CAS / 冕宁县冶勒自然保护区”; “HOLOTYPE ♂/ Pterostichus (Circinatus) / camelus new species / des. SHI H.L. 2015” [red label]. **Paratypes, a total of 2 females**: 1 female (IZAS): same data as holotype. 1 female (IZAS): “China, Sichuan, Mianning county, Yele reserve, 2988 m, mixed forest; N28.96508 E102.16137; 2012.VI.22 day, under dead log; SHI Hongliang, YANG Ganyan & LIU Ye lgt.”

##### Diagnosis.

Body rather elongate; pronotum with three or four mid-lateral setae; hind angle completely rounded; basal fovea almost impunctate; fifth tarsomeres setose beneath; males with two large tubercles on penultimate sternum.

This species can be easily distinguished from all other *Circinatus* species of *agilis*-group (with multi-setae on pronotal lateral margin) by the setose fifth tarsomeres, and by the very special male secondary sexual character on penultimate sternum.

##### Description.

Body form fairly slender, body length 12.7–13.5 mm; dorsal side dark brown, moderately shining; elytron without iridescent shine; mouthparts, antenna, tarsus, and apex of tibia yellowish brown; ventral side brownish. Both sexes with distinct isodiametric microsculpture on elytron. **Head**. Frons without punctures; antenna reaching elytron basal fifth; right mandible without distinct tooth; left mandible with a tooth near base; gena a little shorter than length of eye, very briefly tumid behind eye. **Pronotum** distinctly narrowed to base, lateral margin almost straight before hind angle, widest before middle, PW/PL = 1.13–1.20; three or four mid-lateral setae present, first one close to anterior angle, last one near middle of lateral margin, setae usually evenly separated; posterior seta far distant from hind angle, distance between seta and hind angle approx same as distance between hind angle and inner basal foveal groove; hind angle completely rounded; basal fovea shallow, faintly defined; inner groove subparallel to median line, curved outwards; outer groove completely vanished, outer area of inner groove faintly depressed; basal foveal area more or less punctate around inner groove. **Elytron** oviform, with basal ridge almost horizontal; elytral shoulder distinctly narrowed, basal ridge and lateral margin forming obtuse angle, humeral tooth indistinct; intervals feebly convex; striae moderately deep, with faint punctures inside; scutellar stria short and complete; third interval with two setigerous pores adjacent to second stria; umbilical pore series on ninth interval sparse in middle, composed of 15–17 pores (6, 1–2, 8–9). **Ventral side**. Proepisternum and mesepisternum finely punctate; metepisternum impunctate; penultimate sternum of males (Fig. 118) with two large tubercles in middle, occupying posterior half of sternum, primary setae of penultimate sternum inserted on outer surface of each tubercle; male terminal sternum not modified. **Legs**. Fifth tarsomeres with three to four pairs of setae beneath; males with apical half of mesotibia not widened, inner margin not crenulate; first metatarsomere with distinct carina on outer surface, such carina on second and third metatarsomeres superficial. **Male genitalia**. Median lobe of male genitalia bent less than 90 degrees, apex gradually bent ventrally (Fig. 28A); ventral margin straight in middle, dorsal margin gradually curved; apical orifice large, slightly turned to left side, not opened on ventral side; apical lamella long, approx one third length of apical orifice, laminate with apex slightly thickened; in dorsal view, apical lamella slightly inclined right, nearly triangular with rounded apex, length approx equal to its basal width (Fig. 28B). Right paramere straight and stout, inner margin slightly expanded near middle, length approx 2.5 times greatest width, apex rounded (Fig. 28C). Endophallus not studied. **Female genitalia**. Spermatheca with seminal canal approx five times as long as receptaculum; receptaculum capitate (Fig. 60), club approx half length of receptaculum; seminal canal inserted at base of common oviduct, base of seminal canal sclerotized. Stylomere II with two ensiform setae at outer margin and one at basal third of inner margin; two short nematiform setae located in a furrow near apex. Female sternum VIII (Fig. 76B) with dense and fine spines on posterior margin; posterior margin curved, deeply notched in middle; posterior region chitinized, anterior region semi-chitinized, middle transparent region V-shaped, adjacent to posterior notch in middle; three transparent patches present on each side. Female tergum VIII (Fig. 76A) with major portion chitinized, mid-posterior region semi-chitinized with denser spots.

##### Distribution.

This species is known only from a single locality in Yele Reserve, Sichuan, Liangshan Yi Autonomous Prefecture. (Map 2)

##### Etymology.

The scientific name *camelus* comes from the generic name of the camel, referring to the two large tubercles on the male penultimate sternum of this new species, which are reminiscent of the Bactrian camel (*Camelus bactrianus*).

##### Affinities.

This new species seems to be allied to *Pterostichus agilis* in that both species have male secondary sexual characters on the penultimate sternum and similar aedeagi.

##### Habitat.

*Pterostichus camelus* sp. n. was collected in mixed forest with dominant large pines, rich in dead logs. Two specimens were collected by pitfall trap, and the third was found under or in dead logs. In Yele Reserve, *Pterostichus camelus* sp. n. was found together with two other *Circinatus* species, *Pterostichus cavazzutianus mianningensis* ssp. n., and *Pterostichus zhygealu* sp. n.

##### Variation.

One female paratype has the elytral third and fourth striae briefly fused at approx basal third.

#### Pterostichus (Circinatus) dimorphus sp. n.

Taxon classificationAnimaliaColeopteraCarabidae

http://zoobank.org/7A78F55C-4284-4C51-97A7-9778022866EC

Chinese common name: 两型通缘步甲 (Liăng Xíng Tōng Yuán Bù Jiă)

Figures 17 , 18 , 44 , 65 , 81 , 103 , 123 , 133

##### Type locality.

Yunnan: Dayao County, Xiaobaicaoling, Zhuanwanhe station (N26.07700°, E101.03183°), altitude 2881 m.

##### Type material.

**Holotype** (IZAS): male, body length = 11.8 mm, pin mounted, genitalia dissected and glued on plastic film pinned under specimen, “CHINA, Yunnan, Dayao, / Santai town, Xiaobaicaoling / Mt., Zhuanwanhe con. stat. / N26.07700, E101.03183"; “2881 m, 2012.V.29 night, / mixed forest, / SHI Hongliang, LIU Ye leg. / Institute of Zoology, CAS / 三台乡小白草岭转弯河”; “HOLOTYPE ♂/ Pterostichus (Circinatus) / dimorphus new species / des. SHI H.L. 2015” [red label]. **Paratypes, a total of 3 males and 2 females**: 1 female (IZAS): the same data as holotype. 1 male, 1 female (IZAS): “CHINA, Yunnan, Dayao, Santai town, pass of Xiaobaicaoling, 3105 m, N26.05615, E101.05448, 2012.V.30 day, in dead wood, mixed forest, SHI Hongliang leg.”. 1 male (IZAS): “CHINA, Yunnan, Dayao, Santai town, pass of Xiaobaicaoling, 3105 m, N26.05615, E101.05448, 2012.V.29 day, under stone, mixed forest, SHI Hongliang leg.”. 1 female (IZAS): “CHINA, Yunnan, Dayao, Santai town, pass of Xiaobaicaoling, 3105 m, N26.05615, E101.05448, 2012.V.30, pit fall trap, mixed forest, SHI Hongliang & LIU Ye leg.”

##### Diagnosis.

Pronotum with single mid-lateral seta; hind angle completely rounded; basal fovea almost impunctate; each sex with different elytral microsculpture: granular in females (elytron luster dull), and isodiametric as usual in males; terminal sternum of male weakly depressed in middle, with two sharp teeth near posterior margin pointing to apex of sternum.

The females of this new species can be rapidly distinguished from all the other *Circinatus* species by the special granular elytral microsculpture. The males are similar to *Pterostichus liciniformis*. In addition to the different sexual characters on the male terminal sternum, *Pterostichus liciniformis* also differs from the new species by its smaller and stouter body form.

##### Description.

Body form fairly slender, body length 11.5–11.9 mm; dorsal side almost black, moderately shining, female elytron dull; elytron without iridescent shine; mouthparts, antenna, tarsus, and apex of tibia dark brown; ventral side blackish. Microsculpture isodiametric on vertex, transverse on pronotum. Elytral microsculpture different in each sexes: isodiametric as usual in males; granular in females, making elytral luster much duller in females. **Head**. Frons without punctures; antenna hardly reaching elytron base; gena approx same length as eye, briefly tumid behind eye. **Pronotum** narrowed to base, posterior margin a little narrower than anterior margin; lateral margin evenly rounded, widest a little before middle, PW/PL = 1.16–1.21; one mid-lateral seta present, located a little before greatest width; posterior seta far distant from hind angle, distance between seta and hind angle greater than distance between hind angle and inner basal foveal groove; hind angle completely rounded; basal fovea shallow, faintly defined; inner groove subparallel to median line, basal half oblique outwards; outer groove completely vanished, outer area of inner groove flat; basal foveal area with sparse and fine punctures on inner side of inner groove. **Elytron** oviform, with basal ridge slightly curved; elytral shoulder slightly narrowed, basal ridge and lateral margin forming obtuse angle, humeral tooth small but distinct; intervals almost even; striae moderately deep, with faint punctures inside; scutellar stria short, apex free; third interval with two setigerous pores adjacent to second stria; umbilical pore series on ninth interval sparse in middle, composed of 14–16 pores (5–6, 1–2, 7–9). **Ventral side**. Proepisternum impunctate, mesepisternum and metepisternum finely punctate; male terminal sternum weakly depressed in middle, with two sharp teeth close to posterior margin pointing to apex of sternum (Fig. 123). **Legs**. Fifth tarsomeres glabrous beneath; males with apical half of mesotibia slightly widened, inner margin not crenulate; only first metatarsomere with carina on outer surface, fairly distinct. **Male genitalia**. Median lobe of male genitalia bent approx 90 degrees, apex nearly straight, a little bent ventrally (Fig. 44A); ventral margin straight in middle, dorsal margin gradually curved; apical orifice large, slightly turned to left side, largely opened on ventral side (Fig. 133); in lateral view, apical lamella laminate, slightly twisted, apex not thickened, its length approx one fifth length of apical orifice; in dorsal view, apical lamella narrow and small, located on right side, pointing apically, length approx 1.5 times basal width, apex rounded (Fig. 44B). Right paramere straight and stout, inner margin slightly expanded near middle, length approx 3.5 times greatest width, apex rounded (Fig. 44C). **Endophallus** (Figs 44D, E, F) very thick, bent to ventral side from ventral opening of apical orifice, major portion of endophallus located on left-ventral side of aedeagus; gonopore (**gp**, gonopore lobe folded in Fig. 44) located close to aedeagal base, pointing to right side. Four distinct lobes recognized: left lobe (**lf**) large, apex bifid in left view, decorated with fine scales, located on left side of endophallus; right lobe (**rl**) large, pointed to right side with truncate apex in ventral view, located on ventral side of endophallus, adjacent to vl, with a small pigmented patch near base; ventral lobe (**vl**) smaller than rl, composed of two sub-lobes, with a narrow pigmented band across its full width; pre-apical lobe (**pa**) indistinct, weakly pointed, just before gp; basal lobe absent. Ventral piece (**vp**, only partly shown in Fig. 44F) is a short and arcuate chitinized piece, located just behind gp. **Female genitalia**. Spermatheca with seminal canal approx two times as long as receptaculum; receptaculum clavate (Fig. 65); seminal canal inserted at base of common oviduct, base of seminal canal with a very fine sclerotized spine. Stylomere II with two ensiform setae at outer margin and one at middle of inner margin; two short nematiform setae located in a furrow near apex. Female sternum VIII (Fig. 81B) with dense and fine setae on posterior margin; posterior margin curved, deeply notched in middle; posterior region chitinized, anterior region semi-chitinized, pigmented with sparse denser areas; anterior margin notched in middle; middle transparent region arrow-shaped, adjacent to posterior and anterior notches in middle. Female tergum VIII (Fig. 81A) with most of region semi-chitinized, pigmented with dense spots, only lateral-anterior region with two chitinized patches.

##### Distribution.

This species is known only from Xiaobaicaoling Mts., Dayao County of north Yunnan (Map 1). The two collecting localities are very close, approx 1 km apart. The altitude range is 2881–3105 m.

##### Etymology.

The scientific name “*dimorphus*” of the new species comes from Greek meaning “two forms”, referring to the different elytral luster between male and female.

##### Affinities.

This new species appears close to *Pterostichus liciniformis* and has the following similarities: the pronotal hind angle completely rounded, males with the terminal sternum distinctly concave, and the median lobe of the aedeagus with the apical orifice opened ventrally. These two species are the only members of the *baenningeri*-group that have the typical *Circinatus* pronotum (posterior seta distant from hind angle; hind angle completely rounded). However, based on aedeagal characters (apical orifice largely opened on the ventral side and apical lamella abruptly narrowed after the apical orifice), *Pterostichus dimorphus* sp. n. could also be close to *Pterostichus wangjiani*.

##### Habitat.

*Pterostichus dimorphus* sp. n. was collected in mixed forest with dominant large pines and rhododendron trees approx 3 – 5 m tall. Some individuals were found inside dead logs in day time, others were found running along tree trunks during night or were taken by pitfall traps.

#### Pterostichus (Circinatus) maitreya sp. n.

Taxon classificationAnimaliaColeopteraCarabidae

http://zoobank.org/3919AFBF-E5D9-456A-8239-0D2C5FAD3120

Chinese common name: 弥勒通缘步甲 (Mí Lè Tōng Yuán Bù Jiă)

Figures 13 , 41 , 63 , 79 , 99 , 120

##### Type locality.

Guizhou: Fanjingshan Mt. (N27.90°, E108.70°), altitude 1778–1973 m.

##### Type material.

**Holotype** (IZAS): male, body length = 13.5 mm, pin mounted, genitalia dissected and glued on plastic film pinned under specimen, “CHINA, Guizhou, Jiangkou, / Fanjing Mt. S. Slope, 4500–/ 5300 steps, 1778–1973 m / N27.90180, E108.70372–/ N27.90784, E108.70052"; “2012.VIII.25 night, on tree / trunk, broadleaf forest, SHI / Hongliang, HUANG Xinlei, / LIU Yizhou leg. IOZ, CAS / 梵净山4500–5300步”; “HOLOTYPE ♂/ Pterostichus (Circinatus) / maitreya new species / des. SHI H.L. 2015” [red label]. **Paratypes, a total of 4 males and 1 female**: 1 male (IZAS): “CHINA, Guizhou, Fanjing Shan, 2300 m, 2001.7.31, Liang H.B. leg.”. 2 males, 1 female (IZAS): “ CHINA, Guizhou, Fanjing Shan, 2000 m, 2001.8.1, Liang H.B. leg.”. 1 male (IZAS): “CHINA, Guizhou, Jiangkou, Fanjing Mt. south slope, 4500 steps (Huixiangping), N27.90180, E108.70372, 1778 m, 2012.VIII.25 day, on ground, broadleaf forest, HUANG Xinlei leg. “.

##### Diagnosis.

Pronotum with single mid-lateral seta; posterior seta located almost at hind angle; hind angle forming an indistinct obtuse angle; elytron with linear microsculpture; males with two small tubercles on terminal sternum; fifth tarsomeres glabrous beneath.

There are five *Circinatus* species (*Pterostichus maitreya*, *Pterostichus baenningeri*, *Pterostichus yan*, *Pterostichus ailaoicus* and *Pterostichus miao*) similar in the following external and genital characters: (1) male terminal sternum with two tubercles; (2) pronotal posterior seta very close to hind angle; (3) aedeagus with median lobe slender, apical lamella located on the right side of apex, narrower than half width of apical orifice apex; (4) elytral microsculpture linear, sometimes hardly visible. These five species are considered to be closely allied with each other. The differences among them are listed in Table 1.

These five species have relatively similar median lobe of aedeagus, but significant differences are expected to be found in the endophallus. We studied only *Pterostichus baenningeri* and *Pterostichus maitreya* sp. n. Most endophallic lobes of these two species can be recognized as homologous, but their shapes and positions are all different (Figs 40, 41).

##### Description.

Body form slightly elongate, relatively large species within subgenus, body length 13.0–13.8 mm; dorsal side almost black, moderately shining, elytron with iridescent shine; mouthparts, antenna, tarsus, tibia, and apex of femur reddish brown; ventral side blackish. Both sexes with elytral microsculpture weak and linear. **Head**. Frons without punctures; antenna reaching elytron basal fifth; gena shorter than length of eye, briefly tumid behind eye. **Pronotum** nearly round, lateral margin strongly curved, widest a little before middle; posterior margin a little narrower than anterior margin; PW/PL = 1.13–1.17; one mid-lateral seta present, located a little before greatest width; posterior seta very close to hind angle; hind angle forming an indistinct obtuse angle; basal fovea shallow, faintly defined; inner groove subparallel to median line, basal half oblique outwards; outer groove very faint, indistinct but present, close to hind angle, approx one fourth length of inner one, outer area of inner groove flat; basal foveal area with fine punctures on inner side of inner groove. **Elytron** oviform, with basal ridge slightly curved; elytral shoulder moderately widened, shoulder angle between basal ridge and lateral margin forming obtuse angle, humeral tooth very small, not pointed; intervals feebly convex; striae moderately deep, without punctures; scutellar stria short, apex free; third interval with two setigerous pores adjacent to second stria; umbilical pore series on ninth interval sparse in middle, composed of 16–17 pores (6, 1–2, 9–10). **Ventral side**. Proepisternum and mesepisternum impunctate, metepisternum finely punctate; male terminal sternum with two small tubercles, located a little before middle of sternum, region between tubercles slightly depressed. (Fig. 120). **Legs**. Fifth tarsomeres glabrous beneath; males with apical half of mesotibia widened, inner margin crenulate; first two metatarsomeres with distinct carina on outer surface, such carina on basal half of third metatarsomere superficial. **Male genitalia**. Median lobe of male genitalia bent approx 90 degrees, apex abruptly bent ventrally (Fig. 41A); ventral margin straight in middle, strongly bent ventrally near apex; dorsal margin gradually curved; apical orifice large, slightly turned to left side, opened on ventral side; in lateral view, apical lamella short, laminate with base slightly thickened, not twisted, its length approx one eighth length of apical orifice; in dorsal view, apical lamella short and rounded, located on right side of median lobe apex, pointing apical-ventrally, its base distinctly grooved on dorsal surface; length of apical lamella approx equal to its basal width (Fig. 41B). Right paramere straight and stout, subtriangular, apical half slightly enlarged, apex strongly narrowed and slightly hooked, length approx three times greatest width, apex pointed (Fig. 41C). **Endophallus** (Fig. 41D, E, F) bent to ventral side across left side of aedeagus, major parts of endophallus located on ventral side of aedeagus (in lateral view); gonopore (**gp**, gonopore lobe folded in Fig. 41) located at level a little before apical lamella, pointing to aedeagal base. Six distinct lobes recognized: basal lobe (**bl**) small and compressed, close to base of apical lamella, lower surface strongly chitinized; dorsal lobe (**dl**) small and rounded, close to apex of apical lamella, membranous without decoration; ventral lobe (**vl**) large, completely rounded, located on ventral side of endophallus, decorated with very fine scales; pre-apical lobe (**pa**) close to lf, evenly rounded; right lobe (**rl**) small and helicon-shaped, located on base of vl, decorated with fine scales; left lobe (**lf**) largest, on left side of endophallus, just before gp, elongated and slightly bent, decorated with large spines, gradually changing to fine scales from lobe apex to gp. Endophallus without any chitinized piece. **Female genitalia**. Spermatheca with seminal canal approx five times as long as receptaculum; receptaculum capitate (Fig. 63), club approx half length of receptaculum; seminal canal inserted at base of common oviduct, base of seminal canal not sclerotized. Stylomere II with two ensiform setae at basal half of outer margin, and one near middle of inner margin; two short nematiform setae located in a furrow near apex. Female sternum VIII (Fig. 79B) with sparse fine setae on posterior margin; posterior margin almost straight, slightly notched in middle; posterior region chitinized; anterior region semi-chitinized, without denser pigmented spots, deeply notched in middle; middle transparent region triangular, adjacent to anterior and posterior notches in middle. Female tergum VIII (Fig. 79A) with major portion semi-chitinized, without denser pigmentation; lateral-anterior region with two chitinized patches; anterior margin slightly notched in middle, posterior margin arcuate.

##### Distribution.

This species is known only from Fanjingshan Mt. in Guizhou Province (Map 1). The altitude range is 1778–2300 m.

##### Etymology.

This new species is named for Maitreya Buddha. Its type locality Fanjingshan Mountain (= Mount Fanjing) is one of the sacred Buddhist mountains of China, and is traditionally regarded as the bodhimanda of *bodhisattva Maitreyabuddha* (Mi Le Fo in Chinese).

##### Affinities.

*Pterostichus maitreya* sp. n. is close to *Pterostichus baenningeri*, *Pterostichus yan* sp. n., *Pterostichus ailaoicus* sp. n. and *Pterostichus miao* sp. n. in their similarities of male terminal sternum and male genitalia (see diagnosis above).

##### Habitat.

Specimens of *Pterostichus maitreya* sp. n. were collected from mid-high altitude mixed forest at Fanjingshan Mountain. They were found running along living tree trunks during the night. It is presumed that this species hides under bark in the day, and hunts on living tree trunks at night.

#### Pterostichus (Circinatus) cavazzutianus mianningensis subsp. n.

Taxon classificationAnimaliaColeopteraCarabidae

http://zoobank.org/F72E714C-7DED-4B7A-A9E6-095CB591E765

Chinese common name:卡瓦通缘步甲冕宁亚种 (Kă Wă Tōng Yuán Bù Jiă Miăn Níng Yà Zhŏng)

Figures 6 , 36 , 58 , 73 , 92 , 115

##### Type locality.

Sichuan: Mianning County, Yele (N28.96508°, E102.16137°), altitude 2988 m.

##### Type material.

**Holotype** (IZAS): male, body length = 11.4 mm, pin mounted, genitalia dissected and glued on plastic film pinned under specimen, “CHINA, Sichuan, Mianning / county, Yele reserve, / 2988 m, mixed forest; / N28.96508 E102.16137"; “2012.VI.24, pit fall trap; SHI / Hongliang, YANG Ganyan & / LIU Ye lgt., Inst. Zool., CAS / 冕宁县冶勒自然保护区”; “HOLOTYPE ♂/ Pterostichus (Circinatus) / cavazzutianus / mianningensis new subspecies / des. SHI H.L. 2015” [red label]. **Paratypes, a total of 18 males and 21 females**: 14 males, 20 females (IZAS): the same data as holotype. 4 males, 1 female (IZAS): “China, Sichuan, Mianning county, Yele conservation, mixed forest, 2988 m, N28.96508 E102.16137, 2012.VI.22 day, under dead log; SHI Hongliang, YANG Ganyan & LIU Ye lgt.”

##### Diagnosis.

Pronotum usually with three mid-lateral setae; hind angle completely rounded; basal fovea slightly punctate; lateral margin of elytron narrow and deep; male terminal sternum slightly depressed in middle, faintly rugose in depression; apical lamella of aedeagus located on right side of aedeagal apex, basal width a little greater than length.

*Pterostichus cavazzutianus mianningensis* ssp. n. is sympatric with *Pterostichus camelus* sp. n. The latter can be readily distinguished by its larger body size and fifth tarsomeres setose beneath.

Compared to the nominotypical subspecies, in addition to their allopatric distributions, these two subspecies differ in the following four aspects. (1) Male terminal sternum: both subspecies have terminal sternum slightly depressed in males, but in *Pterostichus cavazzutianus* s. str., apex of terminal sternum slightly bending downwards, and almost even in *Pterostichus cavazzutianus mianningensis* ssp. n.; inside the depression, *Pterostichus cavazzutianus mianningensis* ssp. n. has faint wrinkles, such wrinkles lacking in *Pterostichus cavazzutianus* s. str. (2) Median lobe of aedeagus: *Pterostichus cavazzutianus mianningensis* ssp. n. has the apical lamella wider, length / basal width approx 0.8; this ratio in *Pterostichus cavazzutianus* s. str. is approx 1.0. (3) Endophallus with four major lobes slightly different: vb-I with its apex pointed in *Pterostichus cavazzutianus* s. str., and completely rounded in *Pterostichus cavazzutianus mianningensis* ssp. n.; vb-II more chitinized and less capitate in *Pterostichus cavazzutianus mianningensis* ssp. n.; va with its piece much wider and shorter in *Pterostichus cavazzutianus mianningensis* ssp. n.; rl less distinct in *Pterostichus cavazzutianus* s. str.; dorsal surface of endophallus slightly angulate in *Pterostichus cavazzutianus* s. str., but evenly curved in *Pterostichus cavazzutianus mianningensis* ssp. n. (4) female sternum VIII: the transparent region small, approx semicircular in *Pterostichus cavazzutianus* s. str., but V-shaped with vague extensions in *Pterostichus cavazzutianus mianningensis* subsp. n.

##### Description.

Body length 10.4–11.6 mm; dorsal side almost black, moderately shining; elytron with faint iridescent shine; mouthparts, antenna, tarsus, and apex of tibia yellowish brown; ventral side brownish. Both sexes with faint linear elytral microsculpture. **Head**. Frons without punctures; antenna reaching elytron basal sixth; gena approx same length as eye, briefly tumid behind eye. **Pronotum** round, widest before middle, PW/PL = 1.10–1.17; usually three (occasional four) mid-lateral setae present, first one close to anterior angle, last one near middle of lateral margin, a little distant from rest ones; posterior seta distant from hind angle, distance between seta and hind angle approx same as distance between hind angle and inner basal foveal groove; hind angle completely rounded; basal fovea shallow, faintly defined; inner groove subparallel to median line, slightly curved outwards; outer groove completely vanished, outer area of inner groove flat; basal foveal area usually sparsely punctate on inner side of inner groove. **Elytron** oviform, basal ridge not oblique; elytral shoulder moderately narrowed, basal ridge and lateral margin forming obtuse angle, humeral tooth very small; intervals feebly convex; striae moderately deep, with fine punctures in basal half; scutellar stria short, complete or not; third interval with two setigerous pores adjacent to second stria; umbilical pore series on ninth interval sparse in middle, composed of 15–16 pores (6, 1–2, 8–9). **Ventral side**. Proepisternum impunctate or slightly punctate near anterior margin; mesepisternum densely punctate; metepisternum sparsely punctate; male terminal sternum slightly depressed, faintly rugose in depression, depression occupying posterior two thirds length of terminal sternum, apex of terminal sternum nearly flat (Fig. 115). **Legs**. Fifth tarsomeres glabrous beneath; males with apical half of mesotibia not widened, inner margin slightly crenulate; first metatarsomere with distinct carina on outer surface, such carina on second metatarsomere superficial. **Male genitalia**. Median lobe of male genitalia bent approx 90 degrees, apex not bent ventrally (Fig. 36A); ventral margin straight before apex, dorsal margin completely curved; apical orifice large, slightly turned to left side, not opened on ventral side; apical lamella short, approx one fourth length of apical orifice, laminate, apex not thickened; in dorsal view, apical lamella distinctly inclined right, nearly triangular with rounded apex, its length approx 0.8 times basal width (Fig. 36B). Right paramere straight and stout, inner margin slightly expanded near middle, length approx 3.5 times greatest width, apex rounded (Fig. 36C). **Endophallus** (Fig. 36D, E, F) short, bent to ventral side across apical lamella, and then turned to aedeagal base; gonopore (**gp**) located at approx same level as apical lamella, pointing to aedeagal base; gonopore lobe (**gpl**) bent to right side of aedeagus. Four distinct lobes recognized: ventral-basal lobe I (**vb-I**) small, close to base of apical lamella, compressed, its upper surface heavily scaled; ventral-basal lobe II (**vb-II**) large and long, close to apical lamella, pointing to ventral face of median lobe, apex a little capitate, upper surface strongly chitinized, lower surface membranous, apex scaled; ventral-apical lobe (**va**) at right side of vb-II, upper surface strongly chitinized, forming a transverse sinuate piece, lower surface membranous; right lobe (**rl**) small and membranous, on right surface of endophallus, weakly pointed. **Female genitalia**. Spermatheca with seminal canal approx three times as long as receptaculum; receptaculum capitate (Fig. 58), club approx half length of receptaculum; seminal canal inserted at base of common oviduct, base of seminal canal sclerotized. Stylomere II with one or two ensiform setae at outer margin, and one at basal third of inner margin; two very short nematiform setae located in a furrow near apex. Female sternum VIII (Fig. 73B) nearly evenly chitinized; posterior margin nearly straight, with fine setae, notched in middle; middle transparent region V-shaped, its anterior margin vaguely defined, adjacent to anterior and posterior notches in middle. Female tergum VIII (Fig. 73A) semi-chitinized, posterior region without denser pigmentation, anterior margin weakly notched in middle, posterior margin evenly arcuate.

##### Distribution.

This species is known only from a single locality in Yele Reserve in Liangshan Yi Autonomous Prefecture, Sichuan province (Map 2).

##### Etymology.

The scientific name comes from the type locality, Mianning County.

##### Affinities.

This new subspecies is proposed for the small but stable differences from the nominotypical subspecies in the male terminal sternum, the median lobe of the aedeagus and the endophallus (see diagnosis above). They were not treated as two distinct species because of their allopatric distributions, identical external appearances, and only very small differences in genital characters. The type localities of these two subspecies are approximately 100 km apart and are at almost the same altitude.

##### Habitat.

*Pterostichus cavazzutianus mianningensis* subsp. n. was collected in mixed forest with dominant giant pines, and rich in dead logs. Most specimens were collected by pitfall trap, and others were found under or in dead logs. In Yele Reserve, *Pterostichus cavazzutianus mianningensis* subsp. n. was found together with two larger-sized *Circinatus* species, *Pterostichus camelus* sp. n. and *Pterostichus zhygealu* sp. n. But *Pterostichus cavazzutianus mianningensis* subsp. n. is much more common than the other two.

#### Pterostichus (Circinatus) miao sp. n.

Taxon classificationAnimaliaColeopteraCarabidae

http://zoobank.org/9BD079AE-6AF9-4B9F-9981-1B76B50F04A8

Chinese common name: 喵通缘步甲 (Miāo Tōng Yuán Bù Jiă)

Figures 14 , 30 , 100 , 121

##### Type locality.

Guangxi: Maoershan Mt. (N25.87°, E110.41°), altitude 2100 m.

##### Type material.

**Holotype** (IZAS): male, body length = 12.5 mm, pin mounted, genitalia dissected and glued on plastic film pinned under specimen, “China, Guangxi Guilin. / Huajiang Gaozhai / Maoershan Nature Reserve”; “2100 m; 2011.VI.3 D / Huang Xinlei Collector. / Inst. of Zoology, CAS / 广西桂林猫儿山保护区”; “HOLOTYPE ♂/ Pterostichus (Circinatus) / miao new species / des. SHI H.L. 2015” [red label]. **Paratype, a total of 1 male**: 1 male (IZAS): “CHINA: Guangxi Prov. / Mt. Maoershan / Huilong Temple / 25°54’N, 110°27’E / alt. 1650m, 23-VII-2012 / ZHU, SONG & HU leg.”.

##### Diagnosis.

Pronotum with single mid-lateral seta; posterior seta very close to hind angle; hind angle nearly rounded, lateral margin slightly sinuate before hind angle; elytral microsculpture linear and faint; elytral shoulder angle completely rounded; males with two small tubercles on terminal sternum; fifth tarsomeres glabrous beneath. For comparisons between similar species see table 1 under *Pterostichus maitreya*.

##### Description.

Body form fairly elongate, body length 12.5 mm; dorsal side blackish, moderately shining, elytron with distinct iridescent shine; mouthparts, antenna, tarsus, tibia, and apex of femur reddish brown; ventral side blackish. Elytral microsculpture very faint, linear. **Head**. Frons without punctures; antenna reaching elytron basal fifth; gena shorter than length of eye, briefly tumid behind eye. **Pronotum** a little elongate, lateral margin curved in middle, slightly sinuate before hind angle, widest at approx anterior third; posterior margin a little narrower than anterior margin; PW/PL = 1.12; one mid-lateral seta present, a little before greatest width; posterior seta close to hind angle; hind angle nearly rounded, not forming obtuse angle; basal fovea shallow, faintly defined; inner groove subparallel to median line, not reaching posterior margin; outer groove completely vanished; outer area of inner groove flat; basal foveal area finely punctate along inner groove. **Elytron** oviform, with basal ridge almost straight; elytral shoulder moderately widened, shoulder angle between basal ridge and lateral margin completely rounded, not forming obtuse angle, without humeral tooth; intervals feebly convex; striae moderately deep, without punctures; scutellar stria short, apex free; third interval with two setigerous pores adjacent to second stria; umbilical pore series on ninth interval sparse in middle, composed of 15 pores (6, 1, 8). **Ventral side**. Proepisternum and mesepisternum finely punctate near anterior margin, metepisternum heavily punctate; male terminal sternum with two very small tubercles, tubercles indistinctly defined, at approx anterior third of sternum, region between tubercles shallowly depressed (Fig. 121). **Legs**. Fifth tarsomeres glabrous beneath; males with apical half of mesotibia slightly widened, inner margin crenulate; first two metatarsomeres with distinct carina on outer surface, such carina on basal half of third metatarsomere superficial. **Male genitalia**. Median lobe of male genitalia bent approx 90 degrees, apex gradually bent ventrally (Fig. 30A); ventral margin slightly curved in middle; dorsal margin gradually curved; apical orifice large, slightly turned to left side, opened on ventral side; in lateral view, apical lamella laminate with base slightly thickened, not sinuate or twisted, its length approx one fifth length of apical orifice; in dorsal view, apical lamella located on right side of aedeagal apex, pointing apical-ventrally, not oriented to left side; apical lamella narrow, longish oviform, slightly widened to apex, apex rounded, its base not grooved on dorsal surface; length of apical lamella approx 1.7 times its basal width (Fig. 30B). Right paramere straight and stout, nearly triangular, apical half slightly enlarged and then narrowed to apex, inner margin slightly sinuate before apex; length approx 2.5 times greatest width; apex obtuse, wider than *Pterostichus baenningeri* (Fig. 30C). Endophallus not studied. Female genitalia unknown.

##### Distribution.

This species is known only from Maoershan Mt. in Guangxi Province (Map 1). The altitude range is 1650-2100 m.

##### Etymology.

This new species is named for its type locality, Maoershan Mt. In Chinese, the mountain name means “mountain of the cat”. A cat, as a common pet, is always given the nickname “Miao” in Chinese.

##### Affinities.

*Pterostichus miao* sp. n. is close to *Pterostichus baenningeri*, *Pterostichus maitreya* sp. n., *Pterostichus ailaoicus* sp. n., and *Pterostichus yan* sp. n. because of the similarity of the male terminal sternum and the male genitalia. Among these five species, *Pterostichus miao* sp. n. is similar to *Pterostichus ailaoicus* sp. n. in male genitalia; both species have relatively long apical lamella, 1.7 or 2.1 times as long as its basal width (approximately the same length as the basal width in the other three species), and the apical lamella of the aedeagus without a groove on the dorsal side (with a distinct groove near the base of the dorsal side in the other three species). However, based on external characters, *Pterostichus miao* sp. n. appears to be unique among these five species: the elytral shoulder angle rounded (forming obtuse angle in the other species), and the male terminal sternum with two tubercles located at approximately the basal third (near the middle in the other species).

#### Pterostichus (Circinatus) tumulus sp. n.

Taxon classificationAnimaliaColeopteraCarabidae

http://zoobank.org/0F976BFE-1BC4-45E5-BB2D-FEA080B20B5B

Chinese common name: 丘通缘步甲 (Qīu Tōng Yuán Bù Jiă)

Figures 20 , 32 , 55 , 83 , 105 , 125

##### Type locality.

Guizhou: Fanjingshan Mt. (ca. N27.90°, E108.70°), altitude 1780 m.

##### Type material.

**Holotype** (IZAS): male, body length 13.7 mm, pin mounted, genitalia dissected and glued on plastic film pinned under specimen, “CHINA, Guizhou / Fanjing Shan, / 1780 m, 2001.8.1"; “Liang H.B. leg. / Institute of Zoology / Chinese Acad. Sci.”; “HOLOTYPE ♂/ Pterostichus (Circinatus) / tumulus new species / des. SHI H.L. 2015” [red label]. **Paratype, a total of 1 female**: 1 female (IZAS): “CHINA, Guizhou, Jiangkou, / Fanjing Mt. S. Slope, 4500–/ 5300 steps, 1778–1973 m / N27.90180, E108.70372–/ N27.90784, E108.70052"; “2012.VIII.25 night, on tree / trunk, broadleaf forest, SHI / Hongliang, HUANG Xinlei, / LIU Yizhou leg. IOZ, CAS / 梵净山4500–5300步”.

##### Diagnosis.

Pronotum with single mid-lateral seta; posterior seta distant from hind angle; pronotum strongly narrowed to base, hind angle forming obtuse angle, lateral margin slightly sinuate before hind angle; elytral microsculpture distinct, approx isodiametric; males with one elongate large tubercle on terminal sternum; fifth tarsomeres glabrous beneath.

The sp. n. differs from other species of *Circinatus* in having the pronotal hind angle not completely rounded, forming an obtuse angle, except for four species (*Pterostichus baenningeri*, *Pterostichus maitreya* sp. n., *Pterostichus ailaoicus* sp. n. and *Pterostichus wangjiani* sp. n.), which also have the hind angle more or less obtuse. But *Pterostichus tumulus* sp. n. can be easily distinguished from latter four species by: (1) pronotal posterior seta distant from hind angle (very close to hind angle in latter four species); (2) elytral microsculpture distinct, almost isodiametric (very faint and linear in latter four species except for *Pterostichus wangjiani* sp. n.); (3) males with one large tubercle on terminal sternum (different characters in latter four species).

In Fanjingshan Mt., *Pterostichus tumulus* sp. n. is sympatric with and almost the same size as *Pterostichus maitreya* sp. n., but they can be easily separated by characters listed above.

##### Description.

Body form a little elongate, body length = 13.7–13.8 mm; dorsal side blackish, moderately shining, elytron without iridescent shine; mouthparts, antenna, tarsus, apex of tibia dark brown; ventral side blackish. Elytral microsculpture distinct, isodiametric or slightly transverse. **Head**. Frons without punctures; antenna reaching elytron basal eighth; gena shorter than length of eye, briefly tumid behind eye. **Pronotum** strongly narrowed to base, lateral margin curved in middle, slightly sinuate before hind angle, widest at approx anterior third; posterior margin much narrower than anterior margin; PW/PL = 1.17–1.18. One mid-lateral seta present, located at greatest width; posterior seta distant from hind angle, distance between seta and hind angle approx equal to distance between hind angle and inner basal foveal groove; hind angle distinct, forming obtuse angle; lateral margin slightly elevated near hind angle. Basal fovea moderately deep but not well defined; inner groove subparallel to median line, almost straight near posterior margin; outer groove faint but present, shallowly engraved near hind angle, approx one third length of inner one; outer area of inner groove deeply depressed; basal foveal area without punctures. **Elytron** oviform, with basal ridge almost straight; elytral shoulder a little widened, shoulder angle between basal ridge and lateral margin forming indistinct obtuse angle, humeral tooth small but present; intervals feebly convex; striae moderately deep, without punctures; scutellar stria long, apex free; third interval with two setigerous pores adjacent to second stria; umbilical pore series on ninth interval sparse in middle, composed of 16–17 pores (6, 1, 9–10). **Ventral side**. Proepisternum almost impunctate, very sparsely punctate near posterior margin; mesepisternum and metepisternum densely and finely punctate. Male terminal sternum with an elongate tubercle on middle, tubercle apex rounded and impunctate; tubercle gradually turned into a short ridge at approx apical two fifths of sternum; ridge not reaching apex of sternum, region after it with fine longitudinal wrinkles (Fig. 125). **Legs**. Fifth tarsomeres glabrous beneath; males with apical half of mesotibia slightly widened, inner margin crenulate; first metatarsomere with distinct carina on outer surface, such carina on basal half of second metatarsomere superficial. **Male genitalia**. Median lobe of male genitalia bent approx 90 degrees, apex abruptly bent ventrally (Fig. 32A); ventral margin straight in middle, then sinuate; dorsal margin gradually curved; apical orifice large, slightly turned to left side, opened on ventral side; in lateral view, apical lamella laminate with base slightly thickened, distinctly sinuate and turned dorsally, its length approx one sixth length of apical orifice; in dorsal view, apical lamella on right side of aedeagal apex, pointing apically, slightly oriented to left side; apical lamella short, roundish, widened to apex, apex rounded, its base grooved on dorsal surface; length of apical lamella approx 1.2 times its basal width (Fig. 32B). Right paramere straight and narrow, nearly digitiform, apical half only slightly enlarged and then gradually narrowed to apex, apex narrowly rounded; length approx 3.7 times greatest width (Fig. 32C). Endophallus not studied. **Female genitalia**. Spermatheca (Fig. 55) with seminal canal approx two times as long as receptaculum; receptaculum clavate, gradually expanded to apex; seminal canal inserted at base of common oviduct, base of seminal canal strongly sclerotized. Stylomere II with two ensiform setae near middle of outer margin, and a smaller one near middle of inner margin; two short nematiform setae located in a furrow near apex. Female sternum VIII (Fig. 83B) short and wide, approx evenly chitinized, without denser pigmentation; posterior margin with sparse fine setae, notched in middle; anterior margin deeply notched in middle; middle transparent region wide and V-shaped, adjacent to anterior notch in middle. Female tergum VIII (Fig. 83A) with major portion well chitinized, with irregular fine spots, a narrow region along posterior margin less chitinized; anterior margin weakly notched in middle, posterior margin slightly arcuate.

##### Distribution.

This species is known only from Fanjingshan Mt. in Guizhou Province (Map 1). The altitude range is approx 1778–1973 m.

##### Etymology.

The scientific name comes from a Latin noun “*tumulus*”, which means small hill, referring the male of new species with a tubercle on terminal sternum.

##### Affinities.

In *Pterostichus tumulus* sp. n., the aedeagus is very similar to *Pterostichus baenningeri*, and the new species is assumed to be close to this and related species (listed in Table 1). These species all have the small apical lamella located on the right side of the median lobe apex and grooved on the dorsal surface. However, *Pterostichus tumulus* sp. n. differs from the other species because of the position of posterior pronotal seta, elytral microsculpture, male terminal sternum character, and shape of the receptaculum. These characters suggest an intermediate position for *Pterostichus tumulus* between *Pterostichus baenningeri* and some species from northern Yunnan, such as *Pterostichus dimorphus* sp. n.

##### Habitat.

*Pterostichus tumulus* sp. n. was collected together with *Pterostichus maitreya* sp. n. from mid-high altitude mixed forest in Fanjingshan Mountain, but seems rarer.

#### Pterostichus (Circinatus) wangjiani sp. n.

Taxon classificationAnimaliaColeopteraCarabidae

http://zoobank.org/F8F56D18-8C2C-4B9C-9C3B-6B7A065AB210

Chinese common name: 王剑通缘步甲 (Wáng Jiàn Tōng Yuán Bù Jiă)

Figures 19 , 43 , 66 , 82 , 104 , 124 , 134 , 136

##### Type locality.

Yunnan: Dongchuan, Jiaozishan Mt. (also called Jiaozixueshan Mt.), N26.08475°, E102.87425° – N26.08553°, E102.86436°, altitude 3405–4223 m.

All three paratypes were collected by pitfall trap in one locality (N26.08383, E102.88794, 3367 m). The holotype was collected under a stone along the way to the highest peak of Jiaozishan (3405–4223 m). It is presumed that the holotype was collected from middle-high altitude (ca. 3400–3700 m).

##### Type material.

**Holotype** (IZAS): male, body length = 10.5 mm, pin mounted, genitalia dissected and glued on plastic film pinned under specimen, “China, Yunnan, Dongchuan, / Fazhe, Jiaozishan Mt., way / from Xiaohai to The Peak; / N26.08475°, E102.87425°- / N26.08553°, E102.86436° “; “3405–4223 m, 2010.V.28; / Shi Hongliang, under stone. / Inst. of Zoology, CAS / 东川轿子山小海至轿子顶”; “IOZ(E) 1891157"; “HOLOTYPE ♂/ Pterostichus (Circinatus) / wangjiani new species / des. SHI H.L. 2015” [red label]. **Paratypes, a total of 1 male and 2 females**: 1 male, 2 females (IZAS): “China, Yunnan, Dongchuan, Fazhe, Jiaozishan Mt., way to Dahai; N26.08383, E102.88794, 3367 m, 2010.V.28, pit fall trap, Shi Hongliang lgt.”.

##### Diagnosis.

Pronotum approx quadrate, with single mid-lateral seta; posterior seta close to hind angle, distance between seta and hind angle less than half distance between hind angle and inner basal foveal groove; hind angle forming obtuse angle; elytron with distinct microsculpture, transverse or isodiametric; males with terminal sternum strongly concave in middle, concavity occupying four fifths length of terminal sternum.

The new species can be distinguished from most other *Circinatus* species by the position of the pronotal hind seta (very close to hind angle), except for *Pterostichus baenningeri* and its related four species (listed in table 1). *Pterostichus wangjiani* sp. n. can be distinguished from them by different elytral microsculpture, as well as by the different male terminal sternum. *Pterostichus baenningeri* and its related species all have two tubercles on male terminal sternum, but in *Pterostichus wangjiani* sp. n., the terminal sternum is strongly concave and without such tubercles.

##### Description.

Body form relatively stout, relatively smaller species within subgenus, body length 9.5–10.5 mm; dorsal side blackish, moderately shining, elytron without iridescent shine; mouthparts, antenna, tarsus, and apex of tibia reddish brown; ventral side blackish. Both sexes with similar elytral microsculpture, isodiametric on disc, gradually turned to transverse on elytral apex and outer intervals. **Head**. Frons without punctures; antenna reaching elytron basal sixth; gena approx same length as eye, briefly tumid behind eye. **Pronotum** approx quadrate, lateral margin weakly curved, widest approx in middle; posterior margin a little narrower than anterior margin; PW/PL = 1.20–1.21; one mid-lateral seta present, a little before middle; posterior seta close to but a little distant from hind angle, distance between seta and hind angle much shorter than distance between hind angle and inner basal foveal groove; hind angle forming indistinct obtuse angle (Fig. 104); basal fovea shallow, faintly defined; inner groove subparallel to median line, basal half oblique outwards; outer groove indistinct but present, close to hind angle, approx one fourth length of inner one, outer area of inner groove slightly convex; basal foveal area almost impunctate. **Elytron** oviform, with basal ridge slightly curved; elytral shoulder moderately widened, shoulder angle between basal ridge and lateral margin completely rounded, humeral tooth absent; intervals feebly convex; striae moderately deep, faintly punctate before middle; scutellar stria short, apex free; third interval with two setigerous pores adjacent to second stria; umbilical pore series on ninth interval sparse in middle, composed of 15–16 pores (6, 1–2, 8–9). **Ventral side**. Proepisternum impunctate, mesepisternum and metepisternum impunctate; terminal sternum of male strongly concave in middle, concavity occupying four fifths length of terminal sternum, border of concavity carinate at anterior half, apex of terminal sternum flat. (Fig. 124). **Legs**. Fifth tarsomeres glabrous beneath; males with apical half of mesotibia slightly widened, inner margin slightly crenulate; first metatarsomere with distinct carina on outer surface, such carina on second metatarsomere superficial. **Male genitalia**. Median lobe of male genitalia bent less than 90 degrees, apex gradually bent ventrally (Fig. 43A); ventral margin evenly curved in middle, turned ventrally near apex; apical orifice large, slightly turned to left side, largely opened on ventral side; in lateral view, apical lamella very short, slightly twisted, laminate, a little thickened, its length approx one eighth length of apical orifice; in dorsal view, apical lamella short and wide, located on right side of aedeagal apex, pointing apically, length approx 0.8 times its basal width, apex rounded (Fig. 43B). Right paramere straight and stout, apical half slightly enlarged, length approx 2.5 times greatest width, apex rounded (Fig. 43C). **Endophallus** (Fig. 43D, E, F) bent to ventral side across left side of aedeagus, major portion of endophallus located on ventral side of aedeagus (in lateral view); gonopore (**gp**, gonopore lobe folded in Fig. 43) located at level much before apical lamella, pointing to right side. Six distinct lobes recognized: basal lobe (**bl**) small, close to apical lamella, decorated with very fine scales; right lobe (**rl**) small and compressed, decorated with fine spines near apex; left lobe I (**lf-I**) largest, basal half decorated with fine scales, apex coniform and well chitinized; left lobe II (**lf-II**) long and coniform, apex chitinized; ventral lobe (**vl**) long and slender, covered by lf-I, apex sharp, strongly prolonged and bent, its upper surface chitinized; pre-apical lobe (**pa**) just before gp, small and coniform, apex clearly chitinized. Besides those six lobes, a patch present close to gp, decorated with dense spines. **Female genitalia**. Spermatheca with seminal canal approx four times as long as receptaculum; receptaculum tubiform (Fig. 66), apical half slightly clavate; seminal canal inserted at base of common oviduct, base of seminal canal sclerotized. Stylomere II with two ensiform setae at outer margin, and one near middle of inner margin; two very short nematiform setae located in a furrow near apex. Female sternum VIII (Fig. 82B) with dense spines on posterior margin; posterior margin almost straight, slightly notched in middle; posterior region chitinized, anterior region semi-chitinized, without denser pigmentation, deeply notched in middle; middle transparent region V-shaped, adjacent to posterior notch but not to anterior notch in middle. Female tergum VIII (Fig. 82A) with major portion semi-chitinized, sparsely pigmented with irregular small spots, only lateral-anterior region with two chitinized patches; anterior margin deeply notched in middle.

##### Distribution.

This species is known only from Jiaozishan Mountain located on the border between Dongchuan and Luquan counties of north Yunnan (Map 1).

##### Etymology.

The new species is named for Dr Wang Jian (Honghe University, Mengzi, Yunnan), a specialist in reptiles and close friend of the first author. With his company and help, the first author explored Jiaozishan Mountain and discovered this interesting new species.

##### Affinities.

At first examination, we did not recognize this species as a member of *Circinatus* because of the unique pronotal shape (pronotum approximately quadrate with an obtuse hind angle). However, after careful study, it was found that this species is very similar to *Pterostichus liciniformis* based on the male terminal sternum and the aedeagus. Moreover, in spite of the pronotal shape, the following important characters are all consistent with *Circinatus*: the pronotal basal foveal grooves faintly defined, the pronotal posterior seta located before the hind angle (although only a short distance before), the third elytral interval with two setigerous pores, the metacoxa with two setae, and the right paramere short and straight. Some species of *Circinatus* also have a somewhat well-defined pronotal hind angle, such as in *Pterostichus maitreya* sp. n. Thus, this new species is included in the subgenus *Circinatus*.

From the position of the pronotal posterior seta and the male secondary sex characters on the terminal sternum, this new species shows an intermediate status between the other two species from northwestern Yunnan (*Pterostichus liciniformis* and *Pterostichus dimorphus* sp. n.) and the other five species (listed in Table 1) of the *baenningeri*-group (*vide infra*) from the eastern provinces. For detailed discussions, see the section on infra-subgeneric taxonomy.

##### Habitat.

Three paratypes of *Pterostichus wangjiani* sp. n. were collected by pitfall trap in mixed forest with dominant *Picea* and *Rhododendron* trees approx 3–5 m tall. The holotype was collected under rocks on open land along path.

#### Pterostichus (Circinatus) yan sp. n.

Taxon classificationAnimaliaColeopteraCarabidae

http://zoobank.org/A98DC829-A8E5-45E8-93CD-8E29DBB5BA84

Chinese common name: 炎通缘步甲 (Yán Tōng Yuán Bù Jiă)

Figures 15 , 31 , 101

##### Type locality.

Hubei: Shennongjia, Muyu (ca. N31.47°, E110.39°), altitude 2000 m.

##### Type material.

**Holotype** (NHMB): male, body length = 12.8 mm, board mounted, genitalia dissected and glued on plastic film pinned under specimen, “CHINA, W HUBEI prov. / Dashennongjia Nat. Res. / Muyu, E slope, 2000 m / 12–15 Jun 1997, Bolm. lgt.”; “HOLOTYPE ♂/ Pterostichus (Circinatus) / yan new species / des. SHI H.L. 2015” [red label].

##### Diagnosis.

Pronotum with single mid-lateral seta; posterior seta close to hind angle; hind angle nearly rounded; elytron with microsculpture hardly visible, linear; males with two small tubercles on terminal sternum; fifth tarsomeres glabrous beneath. For comparisons between similar species see table 1 under *Pterostichus maitreya*.

##### Description.

Body form slightly elongate, relatively larger-sized species within subgenus, body length 12.8 mm; dorsal side blackish, moderately shining, elytron with distinct iridescent shine; mouthparts, antenna, tarsus, tibia, and apex of femur reddish brown; ventral side blackish. Elytral microsculpture very faint, linear. **Head**. Frons without punctures; antenna reaching elytron basal fifth; gena shorter than length of eye, briefly tumid behind eye. **Pronotum** nearly round, lateral margin strongly curved, widest a little before middle; posterior margin narrower than anterior margin; PW/PL = 1.15; one mid-lateral seta present, at approx anterior third; posterior seta very close to hind angle; hind angle nearly rounded, not forming obtuse angle; basal fovea shallow, faintly defined; inner groove subparallel to median line, basal half slightly oblique outwards; outer groove indistinct; outer area of inner groove slightly depressed; basal foveal area distinctly punctate. **Elytron** oviform, with basal ridge almost straight; elytral shoulder moderately widened, shoulder angle between basal ridge and lateral margin forming obtuse angle, humeral tooth very small, a little pointed; intervals feebly convex; striae moderately deep, without punctures; scutellar stria short, apex free; third interval with two setigerous pores adjacent to second stria; umbilical pore series on ninth interval sparse in middle, composed of 16–17 pores (6–7, 2, 8). **Ventral side**. Proepisternum impunctate, mesepisternum and metepisternum densely punctate; male terminal sternum with two small tubercles, tubercles indistinctly defined, a little before middle of sternum, region between tubercles slightly depressed. **Legs**. Fifth tarsomeres glabrous beneath; males with apical half of mesotibia widened, inner margin crenulate; first two metatarsomeres with distinct carina on outer surface, such carina on basal half of third metatarsomere superficial. **Male genitalia**. Median lobe of male genitalia bent approx 90 degrees, apex slightly bent ventrally (Fig. 31A); ventral margin straight in middle, turned ventrally near apex; dorsal margin gradually curved; apical orifice large, slightly turned to left side, opened on ventral side; in lateral view, apical lamella short and slightly sinuate, laminate with base slightly thickened, not twisted, its length approx one eighth length of apical orifice; in dorsal view, apical lamella located on right side of aedeagal apex, pointing apical-ventrally, slightly oriented to left side; apical lamella nearly triangular, gradually narrowed to apex, apex rounded, its base distinctly grooved on dorsal surface; length of apical lamella approx 1.1 times its basal width (Fig. 31B). Right paramere straight and stout, nearly triangular, apical half slightly enlarged, strongly narrowed and slightly hooked to apex; length approx 2.8 times greatest width, apex pointed (Fig. 31C). Endophallus not studied. Female genitalia unknown.

##### Distribution.

This species is known only from the holotype collected from Shennongjia in Hubei Province (Map 1). The altitude is approx 2000 m.

##### Etymology.

This new species is named for Emperor Yan, also called Shennong, who was a mythical emperor in ancient China, and generally regarded as one of the ancestors of all Chinese nations. The place Shennongjia (a mountain in west Hubei province, the type locality of this new species) is named for Emperor Yan as well.

##### Affinities.

*Pterostichus yan* sp. n. is close to *Pterostichus baenningeri*, *Pterostichus maitreya* sp. n., *Pterostichus ailaoicus* sp. n., and *Pterostichus miao* sp. n. in their similarities of male terminal sternum and male genitalia.

#### Pterostichus (Circinatus) yuxiaodongi sp. n.

Taxon classificationAnimaliaColeopteraCarabidae

http://zoobank.org/991E54E3-54D9-485E-AE91-484D523C6096

Chinese common name: 于晓东通缘步甲 (Yú Xiăo Dōng Tōng Yuán Bù Jiă)

Figures 23 , 46 , 108

##### Type locality.

Sichuan: Wolong, Wuyipeng (ca. N31.01°, E103.18°), altitude 2535 m.

##### Type material.

**Holotype** (IZAS): male, body length = 15.5 mm, pin mounted, genitalia dissected and glued on plastic film pinned under specimen, “CHINA, Sichuan, Wenchuan / county, Wolong nat. reserve, / Wuyipeng; 2535 m, mixed / forest; 2004.VI.29–VII.14 / YU Xiaodong lgt., HWM3–2"; “IOZ(E) 1891268"; “HOLOTYPE ♂/ Pterostichus (Circinatus) / yuxiaodongi new species / des. SHI H.L. 2015” [red label].

##### Diagnosis.

Pronotum with single mid-lateral seta; posterior seta distant from hind angle, hind angle completely rounded; basal foveal grooves completely fused, forming simple and deep basal fovea; elytron with faint transverse microsculpture; basal ridge strongly oblique; shoulder angle obtuse, humeral tooth short and obtuse; males with terminal sternum not modified; median lobe of aedeagal apex strongly bent to right side, apical lamella a little shorter than its basal width.

This new species is very similar to *Pterostichus dentifer* and *Pterostichus subtilissimus*. They are all relatively large sized within the subgenus, and have the aedeagal apex strongly bent to the right side. *Pterostichus yuxiaodongi* sp. n. differs from the latter two species by: (1) body form relatively stout, pronotum less narrowed to base (body form a little slenderer, pronotum strongly narrowed to base in the other two species); (2) pronotal basal foveal inner and outer grooves completely fused, forming simple deep fovea (basal fovea bifid anteriorly, inner and outer grooves not fused at anterior part in the other two species); (3) elytral humeral tooth less distinct than the other two species; (4) apical lamella much longer than the other two species.

##### Description.

Body form somewhat elongate, body length 15.5 mm; dorsal side blackish, moderately shining, elytron without iridescent shine; mouthparts, antenna, tarsus, apex of tibia dark brown; ventral side blackish. Elytral microsculpture shallow, transverse. **Head**. Frons without punctures; antenna reaching elytron basal sixth; gena a little shorter than length of eye, briefly tumid behind eye. **Pronotum** a little narrowed to base, lateral margin fairly curved in middle, almost straight before hind angle, widest at approx anterior third; posterior margin a little narrower than anterior margin; PW/PL = 1.17. One mid-lateral seta present, at greatest width. Posterior seta distant from hind angle, distance between seta and hind angle a little shorter than distance between hind angle and inner basal foveal groove; hind angle completely rounded, weakly extended backward; posterior margin slightly emarginate in middle. Basal foveal inner and outer grooves completely fused together, region between them deeply depressed, so that full basal foveal region forms a simple and deep depression; outer margin of depression approx four fifths as long as inner margin; region between pronotal lateral margin and basal fovea approx flat; basal foveal area without punctures, slightly rugose in depression. **Elytron** oviform, with basal ridge strongly oblique lateral-posteriorly; elytral shoulder strongly narrowed, shoulder angle between basal ridge and lateral margin forming indistinct obtuse angle, humeral tooth small and obtuse, but distinct, pointing to lateral-posterior side; intervals moderately convex; striae somewhat deep, without punctures; scutellar stria moderately long, apex conjunct to first stria; third interval with two setigerous pores adjacent to second stria; umbilical pore series on ninth interval sparse in middle, composed of 17–18 pores (6, 3, 8–9). **Ventral side**. Proepisternum impunctate; mesepisternum and metepisternum very sparsely punctate near anterior margin. Terminal and penultimate sterna of males not modified. **Legs**. Fifth tarsomeres glabrous beneath; males with apical half of mesotibia not widened, inner margin simple; first two metatarsomeres with distinct carina on outer surface, such carina absent on third metatarsomere. **Male genitalia**. Median lobe of male genitalia bent approx 90 degrees, apex not turned ventrally (Fig. 46A); in lateral view, ventral margin straight in middle, and then straight before apex; dorsal margin slightly curved; apical orifice large, located on dorsal side, not turned to left, not opened on ventral side; apical lamella laminate, not turn dorsally or ventrally. In dorsal view, median lobe strongly bent to right side and gradually narrowed after middle of apical orifice; apical orifice not constricted in middle; apical lamella approx quadrate, its length approx 0.8 times its basal width, pointing right-apically; apex truncate; left margin of apical lamella straight; right margin shorter and sinuate, forming an indistinct obtuse angle near apex (Fig. 46B). Right paramere straight, shorter than other species in subgenus; apical half distinctly enlarged, nearly rounded, very faintly bent to its right side; apex completely rounded; length approx 2.4 times greatest width (Fig. 46C). **Endophallus** (Figs 46D, E, G) without any chitinized piece; bent to dorsal-apical side of aedeagus, all parts of endophallus located on dorsal side of aedeagus; gonopore (**gp**, gonopore lobe folded in Fig. 46) located at level a little beyond apical lamella, pointing to aedeagal base; dense and heavy spines present on dorsal surface of endophallus close to gp. Three major lobes recognized: basal lobe (**bl**) large and rounded, located at level of apical lamella, on right side of endophallus; middle lobe (**ml**) approx same size as bl, apex finely scaled, located adjacent to dorsal face of median lobe; pre-apical lobe (**pa**) small, tubiform, between ml and gp, without decoration. Female genitalia unknown.

##### Distribution.

This species is known only from the holotype collected in Wolong, Sichuan Province (Map 2). The altitude is 2535 m.

##### Etymology.

The new species is dedicated to our friend, Dr Yu Xiaodong (IZAS), who conducted a series of fruitful expeditions in West Sichuan, and collected a large number of interesting *Pterostichus* specimens, including this new species.

##### Affinities.

*Pterostichus yuxiaodongi* sp. n. is closest to *Pterostichus dentifer* and *Pterostichus subtilissimus*. These three species are considered to form a species group within the subgenus (For morphology definition see the discussion under infra-subgeneric taxonomy).

##### Habitat.

The new species was collected by pitfall trap in mixed forest. Along with a very large number of *Pterostichus* specimens of other species, only one specimen of *Pterostichus yuxiaodongi* sp. n. was collected in the same batch. So this new species is presumed to be very rare at its type locality, Wolong.

#### Pterostichus (Circinatus) zhygealu sp. n.

Taxon classificationAnimaliaColeopteraCarabidae

http://zoobank.org/BB708E40-C604-41BD-AFD2-2389FD3162C7

Chinese common name: 彝王通缘步甲 (Yí Wáng Tōng Yuán Bù Jiă)

Figures 8 , 38 , 54 , 75 , 94 , 117

##### Type locality.

Sichuan: Dafengding national reserve, Yizi pass between Meigu and Ebian County (N28.67477° E103.05248°), altitude 2923 m.

##### Type material.

**Holotype** (IZAS): male, body length = 13.5 mm, pin mounted, genitalia dissected and glued on plastic film pinned under specimen, “CHINA, Sichuan prov., Yizi / pass btw. Meigu county and / Ebian county, mixed forest, / 2923 m, / N28.67477 E103.05248"; “2012.VI.12, in dead log, / SHI Hongliang, LIU Ye leg., / Institute of Zoology, CAS. / 美姑县—峨边县椅子垭口”; “HOLOTYPE ♂/ Pterostichus (Circinatus) / zhygealu new species / des. SHI H.L. 2015” [red label]. **Paratypes, a total of 3 males and 7 females**: 1 male, 2 females (IZAS): the same data as holotype. 2 males, 5 females (IZAS): “CHINA, Sichuan Prov., Yizi pass btw. Meigu county and Ebian county, mixed forest; N28.67477 E103.05248, 2923 m; 2012.VI.15; by pit fall trap; SHI Hongliang & LIU Ye leg.”.

##### Non-type material.

1 female (IZAS): “CHINA, Sichuan, Mianning county, Yele conservation, mixed forest, N28.96508, E102.16137; 2988 m, 2012.VI.22, under stone, YANG Xiaodong lgt.”

##### Diagnosis.

Relatively large-sized species within subgenus; pronotum with three or four mid-lateral setae; hind angle completely rounded; outer basal foveal groove faintly defined, approx half length of inner one, outer area of inner groove distinctly concave; elytron with distinct transverse or isodiametric microsculpture; humeral tooth indistinct, not pointed; males with terminal sternum unmodified, penultimate sternum weakly tumid on each side; apical lamella of aedeagus long, slightly hooked to left.

This new species can be easily distinguished from all other species of the *agilis*-group by its distinct larger size. Including the new species, a total of three known *Circinatus* species (the other two are *Pterostichus agilis* and *Pterostichus camelus* sp. n.) have their male secondary sexual characters on the penultimate sternum. *Pterostichus camelus* sp. n. is distinguishable from the others by its fifth tarsomeres setose, and different male penultimate sternum. *Pterostichus agilis* is the species most similar to *Pterostichus zhygealu* sp. n. in their similar male secondary sexual characters on penultimate sternum (weakly tumid on each side). These two species can be separated by: (1) *Pterostichus zhygealu* sp. n. is much larger than *Pterostichus agilis*; (2) *Pterostichus zhygealu* sp. n. have the outer groove of the pronotal basal foveal present, but completely vanished in *Pterostichus agilis*; (3) elytral humeral tooth almost invisible in *Pterostichus zhygealu* sp. n., but sharp and distinct in *Pterostichus agilis*; (4) apical lamella of aedeagus with obtuse hook in *Pterostichus zhygealu* sp. n., but simple in *Pterostichus agilis*.

Among its sympatric species, *Pterostichus zhygealu* sp. n. is most similar to *Pterostichus adelphus* sp. n. In addition to the difference in size, they also differ in elytral microsculpture: isodiametric or transverse in *Pterostichus zhygealu* sp. n., very faint and linear in *Pterostichus adelphus* sp. n.

##### Description.

Body form stout, body length 13.5–14.5 mm; dorsal side black, shining; elytron without iridescent shine; mouthparts, antenna and tarsus dark brown; ventral side blackish. Both sexes with distinct elytral microsculpture, isodiametric on disc, gradually turned to transverse on elytral apex and outer intervals. **Head**. Frons without punctures; antenna reaching elytron basal fifth; gena approx same length as eye, briefly tumid behind eye. **Pronotum** round, widest at approx anterior third, PW/PL = 1.22–1.28; usually four (sometimes three) mid-lateral setae present, first one close to anterior angle, last one near middle of lateral margin, a little distant from others; posterior seta distant from hind angle, distance between seta and hind angle approx same as distance between hind angle and inner basal foveal groove; hind angle completely rounded; basal fovea shallow, faintly defined; inner groove subparallel to median line, slightly curved outwards; outer groove faint, approx half length of inner one, outer area of inner groove distinctly concave; basal foveal area usually impunctate, sometimes with sparse punctures on inner side of inner groove. **Elytron** oviform, with basal ridge weakly oblique; elytral shoulder slightly narrowed, basal ridge and lateral margin forming obtuse angle, humeral tooth indistinct, not pointed; intervals feebly convex; striae moderately deep, without punctures; scutellar stria short, usually incomplete; third interval with two setigerous pores adjacent to second stria; umbilical pore series on ninth interval sparse in middle, composed of 15–18 pores (6, 1–3, 7–9). **Ventral side**. Proepisternum impunctate; mesepisternum finely punctate on anterior half; metepisternum impunctate or very sparsely punctate; penultimate sternum of males very faintly tumid on each side, tumidities close to primary setae, middle region between tumidities faintly depressed (Fig. 117); terminal sternum of males without special structure. **Legs**. Fifth tarsomeres without setae beneath; males with apical half of mesotibia slightly widened, inner margin slightly crenulate; first metatarsomere with distinct carina on outer surface, such carina on second metatarsomere superficial. **Male** **genitalia**. Median lobe of male genitalia bent approx 90 degrees, apex slightly bent ventrally (Fig. 38A); ventral margin straight in middle, slightly sinuate before apex; dorsal margin gradually curved; apical orifice large, slightly turned to left side, not opened on ventral side; apical lamella long, approx half length of apical orifice, laminate, not thickened; in dorsal view, apical lamella distinctly inclined to right, nearly triangular with rounded apex, apex a little enlarged, slightly hooked to left; apical lamella length approx 1.5 times basal width (Fig. 38B). Right paramere straight and fine, length approx 3.5 times greatest width, apex rounded (Fig. 38C). **Endophallus** (Fig. 38 D, E, F) bent to ventral side across apical lamella; gonopore (**gp**, gonopore lobe folded in Fig. 38) located at level of apical lamella, pointing to aedeagal base. Four distinct lobes recognized, the three (vb-I, vb-II, va) located on ventral side of endophallus strongly chitinized: ventral-basal lobe I (**vb-I**) close to base of apical lamella, very small, covered by vb-II, upper surface strongly chitinized, lower surface membranous; ventral-basal lobe II (**vb-II**) larger than vb-I, upper surface strongly chitinized, lower surface membranous, apex of lower surface distinctly projected; ventral-apical lobe (**va**) close to apex of apical lamella, approx same length as vb-II, upper surface strongly chitinized, its apex strongly prolonged and sinuate, lower surface membranous; right lobe (**rl**) small and rounded, decorated with fine scales. **Female genitalia**. Spermatheca (Fig. 54) with seminal canal approx 5.5 times as long as receptaculum; receptaculum capitate, club approx half length of receptaculum; spermathecal gland slightly expanded; seminal canal inserted at base of common oviduct, base of seminal canal sclerotized. Stylomere II with two ensiform setae at outer margin, and one near middle of inner margin; two short nematiform setae located in a furrow near apex. Female sternum VIII (Fig. 75B) with dense and fine spines on posterior margin; posterior margin rounded, shallowly notched in middle; posterior region well chitinized, anterior region also, middle transparent region V-shaped, almost adjacent to posterior notch in middle. Female tergum VIII (Fig. 75A) with anterior half chitinized, posterior half semi-chitinized pigmented with sparse spots, without transparent region in middle.

##### Distribution.

Most specimens of this species were collected from the type locality (Yizi pass) on the border between Meigu and Ebian County (Sichuan, Liangshan Yi Autonomous Prefecture). One female, slightly different from other specimens, was collected in Yele Reserve in Mianning County. (Map 2)

##### Etymology.

The scientific name of the new species comes from Zhygealu, an honored hero in the Yi people’s legend. Yi is the local nation living in Liangshan Yi Autonomous Prefecture (Sichuan Province), where all known species of the *agilis*-group are distributed. This species deserves a hero’s name as the largest-sized of all known *agilis*-group species.

##### Affinities.

This new species is closest to *Pterostichus agilis*. Despite the difference in size, these two species are very similar in the following characters: (1) elytral microsculpture transverse or isodiametric; (2) male secondary sexual characters on the penultimate sternum; and (3) apical lamella of the aedeagus long and bent to the right. The type locality of *Pterostichus agilis* is Luojishan (Sichuan), a mountain approximately 150 km southwest of the Yizi Pass, which is the type locality of *Pterostichus zhygealu* sp. n.

##### Habitat.

*Pterostichus zhygealu* sp. n. was collected in mixed forest with dominant large pines, and rich in dead logs. In daytime, they hide in dead logs, usually under bark. In nighttime, they come out, and can be trapped by pitfalls. *Pterostichus zhygealu* sp. n. lives together with three other *Circinatus* species. Among them, *Pterostichus zhygealu* sp. n. and *Pterostichus adelphus* sp. n. are rarer than the other two species (*Pterostichus bullatus* and *Pterostichus cavazzutianus*).

##### Variation.

One female collected from Mianning County is slightly different from the holotype and the paratypes in the following characters: pronotum narrower, strongly narrowed to base, and body size small with a body length of 12.7 mm. Because of these morphological differences and a different locality (approximately 100 km NW of the type locality), we suspected that it might represent a different subspecies and excluded it from the type series.

### Supplemental notes on previously described species

#### Pterostichus (Circinatus) agilis

Taxon classificationAnimaliaColeopteraCarabidae

Allegro & Sciaky, 2010

Chinese common name: 捷通缘步甲 (Jié Tōng Yuán Bù Jiă)

Figures 7 , 37 , 59 , 74 , 93 , 116

Pterostichus (Circinatus) agilis Allegro and Sciaky 2010: 11 (Original: *Pterostichus (Circinatus)*; holotype deposited in CRS).

##### Type locality.

Sichuan: Xichang, Luojishan, altitude 2200–2800 m.

##### Type material examined.

**Holotype** of *Pterostichus agilis* Allegro & Sciaky (CRS): teneral male, body length = 11.8 mm, board mounted, genitalia dissected and mounted by eupral on plastic film, pinned under specimen, “CHINA S Sichuan / Luojishan 2200–2800 / m 16–25.vii.1996 / leg. S.Kasantsev”; “ HOLOTYPUS ♂ / Pterostichus / (Circinatus)/ agilis sp. n. / Allegro & Sciaky det. 2010” [red label].

##### Non-type material examined

(total 44 specimens). 3 males (IZAS), “China, Sichuan, Puge county, Luojishan, along tourist path, meadow or mixed forest; 2012.VI.8 D, N27.58423, E102.40257– N27.58253, E102.39497, 2549–2805 m, YANG Xiaodong leg.”; 1 female, “China, Sichuan, Puge county, Luojishan, tourist path, mixed forest, N27.58253, E102.39497, 2805 m, 2012.VI.10, under stone, YANG Xiaodong lgt.”; 2 males, 3 females (IZAS), “China, Sichuan, Puge county, Luojishan, near No.1 glacial groove, mixed forest, 2012.VI.10 D, N27.58445, E102.37913, 3470 m, SHI Hongliang & LIU Ye leg.”; 1 male, 3 female (IZAS), “ China, Sichuan, Puge county, Luojishan, near No.1 glacial groove, mixed forest, /b27.58445, E102.37913, 2012.VI.10, pit fall trap, 3470 m, HUANG Hao leg.”; 7 males 5 females (IZAS), “China, Sichuan, Puge county, Luojishan, upper ropeway station, mixed forest; 2012.VI.9 D, N27.58108, E102.38012, 3595 m, YANG Xiaodong leg.”; 3 males, 5 females (IZAS), “China, Sichuan, Puge county, Luojishan, near ropway upper station, N27.58108, E102.38012, 3595 m, 2012.VI.10, pitfall trap, under rhododendra forest, SHI Hongliang & LIU Ye leg.”; 1 male, 4 females (IZAS), “China, Sichuan, Puge county, Luojishan, Heilongtan lake, mixed forest; N27.57913, E102.37397, 3678 m, 2012.VI.9 N, LIU Ye & SHI Hongliang leg.”; 3 males 1 female (IZAS), “China, Sichuan, Puge county, Luojishan, Xiancao lake, mixed forest, N27.57490, E102.36378; 2012.VI.9 D 3848 m, LIU Ye & SHI Hongliang leg.”; 2 females (IZAS), “China, Sichuan, Puge county, Luojishan, Xianya lake, mixed forest; N27.57263 E102.36597, 2012.VI.10 pitfall trap, 3859 m, LIU Ye & SHI Hongliang leg.”.

##### Diagnosis.

Pronotum with three to five mid-lateral setae; hind angle completely rounded; outer basal foveal groove vanished, outer area of inner groove completely flat; elytron with distinct isodiametric microsculpture; elytral humeral tooth distinct, short and sharp, pointed backwards; fifth tarsomeres glabrous beneath; males with terminal sternum unmodified, penultimate sternum slightly tumid on each side; apical lamella of aedeagus long, slightly bent to left.

Based on elytral microsculpture and male sternum, *Pterostichus agilis* is most similar to *Pterostichus camelus* sp. n. and *Pterostichus zhygealu* sp. n. For comparisons between them see the diagnosis of *Pterostichus zhygealu* sp. n.

##### Supplemental description.

**Male sternum**. Penultimate sternum of males faintly tumid on each side, tumidities close to primary setae, middle region between tumidities slightly depressed (Fig. 116); terminal sternum of males without special structures. **Male genitalia**. Median lobe of male genitalia bent approx 90 degrees, apex gradually and slightly bent ventrally (Fig. 37A); ventral margin straight in middle, faintly sinuate before apex; dorsal margin gradually curved; apical orifice large, slightly turned to left side, not opened on ventral side; apical lamella long, approx half length of apical orifice, laminate, not thickened; in dorsal view, apical lamella somewhat inclined right, approx triangular with rounded apex, apex a little prolonged, slightly bent to left, not hooked; apical lamella length approx 1.5 times its basal width (Fig. 37B). Right paramere straight and slender, length approx three times greatest width, apex rounded (Fig. 37C). **Endophallus** (Fig. 37D, E, F) bent to ventral side across apical lamella; gonopore (**gp**) located at level a little beyond apical lamella, pointing to aedeagal base; area around left face of gp decorated with fine scales. Four distinct lobes recognized, the three (vb-I, vb-II, va) located on ventral side of endophallus strongly chitinized: ventral-basal lobe I (**vb-I**) close to left margin of apical lamella base, very small, upper surface decorated with heavy spines and strongly chitinized, lower surface membranous; ventral-basal lobe II (**vb-II**) larger than vb-I, half chitinized half membranous, apex of chitinized right surface spined and slightly enlarged, membranous left surface with elongate apex, forming a digitiform extension on left side (in dorsal view); ventral-apical lobe (**va**) close to apex of apical lamella, evenly chitinized, without membranous region, approx same length as vb-II, broadly triangular with long apex; right lobe (**rl**) small and rounded, decorated with fine scales, located on right side of endophallus, close to gp. **Female genitalia.** Spermatheca with seminal canal approx 3.5 times as long as receptaculum; receptaculum capitate (Fig. 59), club approx two fifths length of receptaculum; spermathecal gland slightly expanded; seminal canal inserted at base of common oviduct, base of seminal canal sclerotized. Stylomere II with two or three ensiform setae at outer margin, and two near middle of inner margin; two short nematiform setae located in a furrow near apex. Female sternum VIII (Fig. 74B) with dense and fine spines on posterior margin; posterior margin slightly rounded, shallowly notched in middle; posterior region well chitinized, anterior region also; middle transparent region V-shaped, almost adjacent to posterior notch in middle. Female tergum VIII (Fig. 74A) evenly chitinized except for darker patches near anterior corner, without spots.

##### Distribution.

So far, this species is known only from Luojishan Mountain in Puge County of south Sichuan (Map 2). The highest peak of Luojishan is 4359 m high. We explored this mountain in 2012. This species was found from altitudes ranging from 2549–3859 m, but most specimens were collected above 3400 m.

##### Affinities.

*Pterostichus agilis* is closest to *Pterostichus zhygealu* sp. n. For details see the discussion under *Pterostichus zhygealu* sp. n. and *agilis*-group below.

##### Remarks.

The holotype is teneral. Its genitalia were distorted when mounting in eupral film. The illustration of the genitalia in the original literature is inadequate, so a redescription is provided of the male genitalia herein based on mature specimens.

#### Pterostichus (Circinatus) baenningeri

Taxon classificationAnimaliaColeopteraCarabidae

Jedlička, 1931

Chinese common name: 本宁通缘步甲 (Běn Níng Tōng Yuán Bù Jiă)

Figures 12 , 40 , 62 , 78 , 98 , 119

Pterostichus (Circinatus) baenningeri Jedlička 1931: 24 (Original: *Pterostichus* (*incertae sedis*); Lectotype deposited in NMPC); Jedlička 1962: 299 (*Pterostichus* (*incertae sedis*)); Sciaky 1996: 221; Bousquet 2003: 488; Lorenz 2005: 280; Allegro and Sciaky 2010: 6.

##### Type locality.

Chongqing: Jinfoshan Mt. (N29.10°, E107.21°). The labels of type series only indicate the locality Chung-King (= Chongqing). But the preface of the original literature (Jedlička 1931) indicated that all material was collected from Kinfushan (= Jinfoshan) of Chung-king (= Chongqing) in May and June 1929.

##### Type material examined.

**Lectotype** (designated herein) of *Pterostichus baenningeri* Jedlička (NMPC): male, body length = 11.0 mm, board mounted, genitalia dissected and mounted on plastic film, pinned under specimen, “CHUNG-KING / Sz tschuan / Coll Dr. Becker”; “TYPE” [red label]; “Mus. Nat. Pragae / Inv. *22374*” [orange label]; ”*Bänningeri* / *type*, *mihi* / Det. Ing. Jedlička” [pink label]. **Paralecototypes** of *Pterostichus baenningeri* Jedlička, 3 males (NMPC) ”CHUNG-KING / Sz tschuan / Coll Dr. Becker”; ”TYPE” [red label]; ”Mus. Nat. Pragae / Inv. *22375*”(22376 and 22377 in other two) [orange label]; ”*Bänningeri* / *mihi* / Det. Ing. Jedlička”. 1 male (MSNM) ”CHUNG-KING / Sz tschuan / Coll Dr. Becker”; ”TYPE” [red label]; ”*Pterostichus* / *Bänningeri* / *sp. n.* / Det. Ing. Jedlička”[pink label].

##### Notes on types.

According to the original description, this species was described based on 11 males, but a holotype was not designated in the original

##### Description.

In the collection of NMPC, a total of four syntypes was found, with the first one (male) bearing a pink determination label and the others bearing white labels. We designate the syntype bearing the pink label, with a serial label no. 22374, as the lectotype.

##### Non-type material examined

(4 specimens). 1 male, 1 female (IZAS), “China, Chongqing, Nanchuan, Jinfoshan Mt., near the peak, stone forest garden. 2185 m, N29.02708, E107.18507, 2012.VIII.19, pit fall trap, broadleaf forest with bamboo, SHI Hongliang, HUANG Xinlei & LIU Yizhou lgt.”; 1 male (IZAS), same data, but: “2012.VIII.17 day, under stone”; 1 male (NHMB), “China, SE Sichuan, Jinfo Shan, 29°01N 107°14E, 1700–1950 m, 24–29.VI.98. J. Schneider”.

##### Diagnosis.

Pronotum with single mid-lateral seta; posterior seta close to hind angle; hind angle forming indistinct obtuse angle; elytron with microsculpture almost invisible; males with two large tubercles on terminal sternum; fifth tarsomeres setose beneath.

This species differs from all other known *Circinatus* species in having the fifth tarsomeres setose beneath, except for *Pterostichus camelus* sp. n. These two species are so different that they can be separated by several characters, such as the different number of pronotal mid-lateral setae, pronotal hind angle shape and so on. *Pterostichus maitreya* sp. n., *Pterostichus baenningeri*, *Pterostichus yan* sp. n., *Pterostichus ailaoicus* sp. n. and *Pterostichus miao* sp. n. are considered close to each other. For comparisons see Table 1 under *Pterostichus maitreya*.

##### Supplemental description.

**Male sternum**. Penultimate sternum not modified; terminal sternum with two large tubercles with indistinct borders, covering approx one third length of sternum; tubercles a little before middle of sternum, distance between tubercles a little greater than distance between setae on penultimate sternum; region between tubercles strongly depressed (Fig. 119). **Endophallus** (Fig. 40D, E, F) bent to ventral side across left side of aedeagus, major portion of endophallus located on ventral side of aedeagus (in lateral view); gonopore (**gp**, gonopore lobe folded in Fig. 40) located at level a little beyond apical lamella, pointing to aedeagal apex. Six distinct lobes recognized: basal lobe (**bl**) close to base of apical lamella, its main portion membranous, decorated with sparse scales, covered by a narrow chitinized piece; ventral lobe (**vl**) very large, bearing two small sub-lobes (**vl-I**, **vl-II**), major portion of vl membranous, sub-lobes with apex strongly scaled; vl-I with heavy and dense scales, its apex darkest in full endophallus; vl-II on right side of vl-I, wider than vl-I; left lobe (**lf**) located on left side of endophallus, a little larger than vl-I, apex rounded and scaled, distant from vl; right lobe (**rl**) small and strongly pointed, clavate with rounded apex, apex scaled; pre-apical lobe (**pa**) very small, on ventral face of endophallus, just before gp, without decoration. Apical patch (**ap**) is a scaled region near gp, not pointed, on right side of endophallus. Endophallus without any chitinized piece. **Female genitalia**. Spermatheca with seminal canal approx two times as long as receptaculum; receptaculum capitate (Fig. 62), club approx half length of receptaculum; seminal canal inserted at base of common oviduct, base of seminal canal not sclerotized. Stylomere II with two ensiform setae at basal half of outer margin, and a small one near middle of inner margin; two short nematiform setae located in a furrow near apex. Female sternum VIII (Fig. 78B) with setae on posterior margin, setae on middle region heavier; posterior margin almost straight, slightly notched in middle; posterior region chitinized, anterior region semi-chitinized, without denser pigmentation, deeply notched in middle; middle transparent region arrow-shaped, adjacent to anterior and posterior notches in middle. Female tergum VIII (Fig. 78A) semi-chitinized, with sparse and very fine spots; lateral-anterior region with darker patches; anterior margin slightly notched in middle, posterior margin evenly arcuate.

##### Distribution.

So far, this species is known only from the type locality, Jinfoshan Mt. of Chongqing, China. (Map 1) The altitude range is 1700–2185 m.

##### Affinities.

*Pterostichus baenningeri* is close to *Pterostichus miao* sp. n., *Pterostichus maitreya* sp. n., *Pterostichus ailaoicus* sp. n. and *Pterostichus yan* sp. n. in their similarities in male terminal sternum and male genitalia. Although the presence of setae beneath the fifth tarsomeres of *Pterostichus baenningeri* is unique in this species group, *Pterostichus baenningeri* seems not to be an isolated species. Thus, the fifth tarsomeres setose beneath should be considered as autopomorphies.

#### Pterostichus (Circinatus) beneshi

Taxon classificationAnimaliaColeopteraCarabidae

Sciaky, 1996

Chinese common name: 贝氏通缘步甲 (Bèi Shì Tōng Yuán Bù Jiă)

Figures 24 , 50 , 51 , 68 , 87 , 109

Pterostichus (Circinatus) beneshi Sciaky 1996: 225 (Original: *Pterostichus (Circinatus)*; holotype deposited in CRS); Bousquet 2003: 488; Lorenz 2005: 280; Allegro and Sciaky 2010: 4.

##### Type locality.

Sichuan: Wolong (ca. N31.03°, E103.18°), Wenchuan County, Sichuan province.

##### Type material examined.

**Holotype** of *Pterostichus beneshi* Sciaky (CRS), male, body length = 11.2 mm, board mounted, genitalia dissected and glued on same board with specimen, “*Cina - W Sichuan* / *Wolong* / *150 Km NW Chengdo* / *9*. *VII*. *94*. *Beneš*.”; “HOLOTYPUS / Pterostichus / (Circinatus)/ beneshi sp. n. / Det. Sciaky, 1994” [red label].

##### Non-type material examined

(total 53 specimens). 3 males, 7 females (IZAS), “Sichuan, Wolong, Wulidun, pit fall trap, YU Xiaodong lgt.”, various altitude between 2290m-2615m, and date between 2004 VI.6–VIII.8; 4 males, 1 female (IZAS), “Sichuan, Wolong, Wuyipeng, pit fall trap, YU Xiaodong lgt.”, various altitude between 2545m-2630m, and date between 2004 VI.14–VIII.14; 8 males, 2 females (IZAS), “Sichuan, Wolong, Wuyipeng, Caoyuandi, pit fall trap, YU Xiaodong lgt.”, various altitude between 2370m-2570m, and date between 2004 V.31–VIII.31; 1 male (IZAS), “Sichuan, Wolong, Wuyipeng, 1990.VII.20, T. Deuve et Xie Weiping”; 2 males (IZAS), “China, Sichuan, Baoxing county, Donglashan gorge park, N30.41675, E102.59366; 2012.VI.28D 1933m, Liu Ye, Shi Hongliang leg.”; 3 males (IZAS), “China, Sichuan, Baoxing county, Jiajinshan mt., Mahuanggou, mixed forest, N30.85669, E102.76615, 2012.VI.29 D 2681m, Liu Ye, Shi Hongliang leg.”; 1 male (IZAS), “China, Sichuan, Baoxing county, Jiajinshan mt., Borigou viewing platform, mixed forest, N30.82233, E102.71886; 3238m, 2012.VI.29, Shi Hongliang, Liu Ye leg”; 1 male (IZAS), “Sichuan, Baoxing, Fengtongzhai, 1995m, 2001.VI.30–VII.3, YU Xiaodong lgt.”; 1 female (IZAS), “Sichuan, Baoxing, Jiajinshan, Mahuanggou, 2495m, 2001.VII.2–5, YU Xiaodong, ZHOU Hongzhang lgt.”; 4 males, 1 female (IZAS), “China, Sichuan, Tianquan, Erlangshan, 2500m, 2011.8.5, Huang Hao lgt.”; 1 male, 2 females (IZAS), “China, Sichuan, Tianquan, Erlangshan, 2500m, 2011.7.21, Huang Hao lgt.”; 5 males, 2 females (IZAS), “China, Sichuan Tianquan county, Erlangshan pass, meadow with shrubs, N29.86240, E102.28123; 2012.VI.20 D, pit fall trap, 2985, Liu Ye, Shi Hongliang leg.”; 2 males (IZAS), “China, Sichuan, Tianquan county, Erlangshan pass, meadow with shrubs, 2985m, N29.86240, E102.28123, 2012.VI.24, pit fall trap, Shi Hongliang, LIU Ye lgt.”; 2 males (IZAS), “Sichuan, Tianquan, Lianglu, Erlangshan, 2229m, 2014.IV.8, Yang Xiaodong lgt.”.

##### Diagnosis.

Pronotum with single mid-lateral seta; posterior seta distant from hind angle, hind angle completely rounded; basal foveal outer groove absent, outer region of inner groove completely flat; basal fovea area finely punctate between two inner grooves; elytral humeral tooth almost invisible; elytron with faint linear microsculpture; fifth tarsomeres glabrous beneath; terminal sternum of male not modified.

This species is very similar to *Pterostichus zoiai*. They can be easily separated by their allopatric distribution. But morphologically, these two species can be separated only with difficulty by: (1) *Pterostichus beneshi* with apical lamella longer, length approx 3/4 of the basal width; *Pterostichus zoiai* with apical lamella shorter, length approx half the basal width; (2) *Pterostichus zoiai* with pronotum relatively narrower as indicated by Allegro and Sciaky (2010: 18, key). These two species are very different in endophallus: *Pterostichus zoiai* with endophallus strongly bent dorsal-basally, the dorsal margin of endophallus closely adjacent to the apical orifice of aedeagus; dl and ll completely fused together. In *Pterostichus beneshi*, the endophallus fully expanded on the dorsal face of the aedeagus, the dorsal margin of the endophallus distinctly separate from the apical orifice; dl and ll well separated. (Figs 49, 50, 51)

##### Supplemental description.

**Male sternum**. Penultimate and terminal sterna without special structures. **Endophallus** (Fig. 50D, E, G) extended in dorsal direction of aedeagus, main portion of endophallus located on dorsal side of aedeagus; gonopore (**gp**) located at level near middle of aedeagus, pointing to right (gonopore lobe folded in Fig. 50). Six distinct lobes recognized: dorsal lobe (**dl**) large with rounded apex, bent backward, close to apical orifice of aedeagus, dorsal half decorated with heavy scales; left lobe (**ll**) a little larger than dl, with finer scales, also bent backward; ventral lobe I (**vl-I**) small, tubiform, pointing in apical direction of aedeagus; ventral lobe II (**vl-II**) small, tubiform, a little longer than vl-I, apex slightly bent to right in dorsal view; right lobe (**rl**) shorter and thicker than vl-II; pre-apical lobe (**pa**) located on right surface, close to gp, apex enlarged, with very fine scales; basal-dorsal region on endophallus decorated with fine and dense scales; endophallus without chitinized piece. **Female genitalia**. Spermatheca with seminal canal approx 3.5 times as long as receptaculum; receptaculum capitate (Fig. 68), club approx half length of receptaculum; seminal canal inserted at base of common oviduct, base of seminal canal with sclerotized region long and thick. Stylomere II with two ensiform setae at basal half of outer margin, and a small one near middle of inner margin; two short nematiform setae located in a furrow near apex. Female sternum VIII (Fig. 87B) with fine setae on posterior margin, setae on middle region thicker; posterior margin narrowly notched in middle; posterior region well chitinized; anterior region weakly chitinized, deeply notched in middle; middle transparent region V-shaped, adjacent to anterior and posterior notches in middle. Female tergum VIII (Fig. 87A) short, posterior region semi-chitinized, with sparse and irregular spots; middle of anterior region with a chitinized large patch; anterior margin notched in middle, posterior margin evenly arcuate.

##### Distribution.

Recorded from four counties of central Sichuan province: Wenchuan (Wolong nature reserve), Dayi (Xilingxueshan), Baoxing (south slope of Jiajinshan), and Tianquan (east slope of Erlangshan). In Wolong and Dayi, this species is sympatric with *Pterostichus xilingensis*, while it is sympatric with *Pterostichus pohnerti* in Baoxing and Tianquan (Maps 2, 3).

##### Affinities.

This species is very close to *Pterostichus zoiai* in their similarities of external appearance and male genitalia. The endophalli are also similar, and all lobes can be recognized as homologous.

##### Geographical variation.

We studied males from three localities: Wolong (N31.03°, E103.18°), Erlangshan (N29.88°, E102.30°), and Baoxing (N30.86°, E102.77°). We found that the Erlangshan population is slightly different from the other two in male genitalia: (1) in the Erlangshan population, the apical lamella is slightly shorter and wider than that in the other two populations and is more similar to that in *Pterostichus zoiai*; (2) in the Erlangshan population, the endophallus with ll is strongly and abruptly narrowed to the apex, the upper margin is strongly sinuate, and the apex of ll is much narrower than that in the other two localities; and (3) in the Erlangshan population, vl-I and vl-II are much thicker than those in the other two localities (Figs 50, 51).

#### Pterostichus (Circinatus) bullatus

Taxon classificationAnimaliaColeopteraCarabidae

Allegro & Sciaky, 2010

Chinese common name: 蟾通缘步甲 (Chán Tōng Yuán Bù Jiă)

Figures 10 , 39 , 61 , 77 , 96 , 128

Pterostichus (Circinatus) bullatus Allegro and Sciaky 2010: 9 (Original: *Pterostichus (Circinatus)*; holotype deposited in collection of Allegro).

##### Type locality.

Sichuan: Ebian, Yizhi Yakou (= Yizi pass, N28.67°, E103.05°), Xiaoliang Shan, Si He (= Xihe, or Heizhugou town), 2800–3100 m.

##### Notes on types.

We did not examine type of this species.

##### Non-type material examined

(total 46 specimens). 16 males, 28 females (IZAS), “China, Sichuan Prov., Yizi pass btw. Meigu county and Ebian county, mixed forest; N28.67477 E103.05248; 2923 m; 2012.VI.15; by pitfall trap; SHI Hongliang & LIU Ye lgt.”; 1 female (IZAS), “China, Sichuan Prov., Yizi pass btw. Meigu county and Ebian county, mixed forest; N28.67477 E103.05248; 2923 m; 2012.VI.12; in dead log; SHI Hongliang & LIU Ye lgt.”; 1 male (IZAS), “China, Sichuan prov., Ebian county, way btw. Lewu xiang and Yizi pass, 2543 m, 2012.VI.15, pit fall trap, LIU Ye & YANG Ganyan lgt.”.

##### Diagnosis.

Pronotum with three to four mid-lateral setae; hind angle completely rounded; elytron with faint transverse microsculpture; even intervals of elytra strongly interrupted, forming large bumps, odd intervals also interrupted but less than even ones; fifth tarsomeres glabrous beneath; males with terminal and penultimate sterna unmodified; apical lamella of aedeagus long, slightly bent to left.

This species can be rapidly distinguished from all other *Circinatus* species by its modified elytral intervals.

##### Supplemental description.

**Male sternum**. Penultimate and terminal sterna of males without special structures. **Endophallus** (Fig. 39D, E, F) bent to ventral side across apical lamella; gonopore (**gp)** located approx at level of apical lamella, pointing to aedeagal base. Four distinct lobes recognized, the three (vb-I, vb-II, va) located on ventral side of endophallus strongly chitinized: ventral-basal lobe I (**vb-I**) close to left margin of apical lamella base, small, upper surface decorated with heavy spines and strongly chitinized, lower surface membranous; ventral-basal lobe II (**vb-II**) with a large T-shaped piece, strongly chitinized, apex truncate and wide, base narrow and long; ventral-apical lobe (**va**) between apex of apical lamella and gp, compressed, strongly chitinized for one half, and membranous for other half; right lobe (**rl**) very small, present as a small hump, not chitinized or decorated, close to gp. **Female genitalia.** Spermatheca with seminal canal approx three times as long as receptaculum; receptaculum capitate (Fig. 61), club approx half length of receptaculum; spermathecal gland slightly expanded; seminal canal inserted at base of common oviduct, base of seminal canal sclerotized. Stylomere II with two or three ensiform setae at outer margin, and two at basal third of inner margin; two very short nematiform setae located in a furrow near apex. Female sternum VIII (Fig. 77B) with dense and fine setae on posterior margin; posterior margin almost straight, strongly notched in middle; anterior margin deeply notched in middle; major part of sternum well chitinized, anterior third semi-chitinized; middle transparent region approx quadrate or semicircular, small, adjacent to posterior notch in middle. Female tergum VIII (Fig. 77A) with anterior region strongly chitinized, posterior half less chitinized than anterior region, posterior half pigmented with fine and irregular spots.

##### Distribution.

So far, this species is known only from mountains between Meigu and Ebian County in south Sichuan (Map 2). Large numbers of specimens were collected from several localities in this area. The altitude range is between 2541–2923 m.

##### Affinities.

With the unique bumps on elytral intervals, this species is one of the most special species in the subgenus. However, because of the similarities in elytral microsculpture and apical lamella of the aedeagus, this species could be close to *Pterostichus agilis* and *Pterostichus zhygealu* sp. n.

##### Variation.

We examined two very special females (Fig. 128): “China, Sichuan Prov., Yizi pass btw. Meigu county and Ebian county, mixed forest; N28.67477 E103.05248; 2923 m; 2012.VI.15; by pitfall trap; SHI Hongliang & LIU Ye lgt.” Unlike the other individuals, these two specimens have elytral striae without bumps, only slightly tangled near the base in one female and completely regular in the other female. Nevertheless, these two females are in perfect accord with *Pterostichus bullatus* in the shape of the pronotum, elytral microsculpture and female sternum VIII characters. These two females are likely individual aberrants instead of a distinct species. For a definitive determination of the taxonomic status of this “form”, male specimens are required.

#### Pterostichus (Circinatus) cavazzutianus s. str. replacement name

Taxon classificationAnimaliaColeopteraCarabidae

Chinese common name: 卡瓦通缘步甲 (Kă Wă Tōng Yuán Bù Jiă)

Figures 5 , 35 , 57 , 72 , 91 , 114

Pterostichus (Circinatus) cavazzutianus s. str. Synonym: *cavazzutii* Allegro & Sciaky, 2010: 8 (Original: *Pterostichus (Circinatus)*; holotype deposited in collection of Allegro). Junior homonym of Pterostichus (Sinosteropus) barbarae cavazzutii Sciaky & Facchini, 2003.

##### Type locality.

Sichuan: Ebian, Yizhi Yakou (= Yizi pass), Xiaoliang Shan, Si He (= Xihe, or Heizhugou Town), 2800–3100 m.

##### Type material examined.

**Paratype** of *Pterostichus cavazzutii* Allegro & Sciaky (CRS): male, board mounted, genitalia dissected and glued on board, pinned under specimen, “CHINA S Sichuan / Pass btw Xihe/Hongxi / Ta Yan Ping, 17.-25.6. / 1999 leg. V. Benes”; “PARATYPUS ♂/ Pterostichus / (Circinatus)/ cavazzutii sp. n. / Allegro & Sciaky det. 2010” [red label].

##### Non-type material examined

(19 specimens). 8 males, 3 females (IZAS), “China, Sichuan Prov., Yizi pass btw. Meigu county and Ebian county, mixed forest; N28.67477 E103.05248; 2923 m; 2012.VI.15; by pitfall trap; SHI Hongliang & LIU Ye lgt.”; 1 female (IZAS), “China, Sichuan Prov., Yizi pass btw. Meigu county and Ebian county, mixed forest; N28.67477 E103.05248; 2923 m; 2012.VI.12;in dead log; SHI Hongliang & LIU Ye lgt.”; 4 males, 1 female (IZAS), “China, Sichuan Prov., Meigu county, Dafengding nat. cons., Hongxi station, mixed forest; 2541 m, N28.65850 E103.06123; 2012.VI.15; by pitfall trap; SHI Hongliang & LIU Ye lgt.”; 1 male, 1 female (IZAS), “China, Sichuan Prov., Ebian county, Heizhugu forestry park, 2888 m, N29.02373 E102.98473; 2012.VI.17; by pitfall trap; rhododendra forest with bamboo, YANG Ganyan & LIU Ye lgt.”.

##### Nomenclature notes.

We here propose *Pterostichus cavazzuttianus* as a replacement name for *Pterostichus cavazzuttii* Allegro & Sciaky, 2010, preoccupied by *Pterostichus cavazzuttii* Sciaky and Facchini, 2003.

##### Diagnosis.

Pronotum usually with three mid-lateral setae; hind angle completely rounded; basal fovea punctate; lateral margin of elytron narrow and deep; males with terminal sternum slightly depressed in middle, not rugose in depression; apical lamella of aedeagus located on right side of aedeagal apex, basal width approx same as length.

For comparisons between *Pterostichus cavazzutianus* s. str. and the subspecies *Pterostichus cavazzutianus mianningensis* subsp. n., see the diagnosis of *Pterostichus cavazzutianus mianningensis* ssp. n. This species is similar to and sympatric with *Pterostichus adelphus* sp. n. For their comparisons see the diagnosis of *Pterostichus adelphus* sp. n.

##### Supplemental description.

**Male sternum**. Penultimate sternum unmodified; terminal sternum slightly depressed in middle, apex of sternum weakly but distinctly bending downwards, almost smooth inside sternum depression (Fig. 114). **Endophallus** (Figs 35D, E, F) short, bent to ventral side across apical lamella, and then turned to aedeagal base; gonopore (**gp**) located at approx same level as apical lamella. Three distinct lobes recognized: ventral-basal lobe I (**vb-I**) small, close to base of apical lamella, compressed, upper surface heavily scaled, apex pointed; ventral-basal lobe II (**vb-II**) large and long, close to apical lamella, pointing to ventral face of median lobe, upper surface strongly chitinized, lower surface membranous, apex capitate and scaled; ventral-apical lobe (**va**) at right side of vb-II, its main body strongly chitinized, forming a large piece with notched apex; right lobe (**rl**) membranous, very small and indistinct. **Female genitalia**. Spermatheca with seminal canal approx three times as long as receptaculum; receptaculum capitate (Fig. 57), club approx half length of receptaculum; seminal canal inserted at base of common oviduct, base of seminal canal sclerotized. Stylomere II with two ensiform setae at outer margin, and one at basal third of inner margin; two very short nematiform setae located in a furrow near apex. Female sternum VIII (Fig. 72B) evenly chitinized; posterior margin with fine and sparse setae, almost straight, notched in middle; middle transparent region small, approx semicircular, without oblique extensions, adjacent to anterior and posterior notches in middle. Female tergum VIII (Fig. 72A) semi-chitinized, posterior region without denser pigmentation, anterior margin without notch in middle, posterior margin evenly arcuate.

##### Distribution.

The nominotypical subspecies is only recorded from localities near the border between Meigu and Ebian County in south Sichuan (Map 2). It is sympatric with three other *Circinatus* species, *Pterostichus bullatus*, *Pterostichus adelphus* sp. n. and *Pterostichus zhygealu* sp. n.

##### Etymology.

The name *Pterostichus cavazzutii* is preoccupied by a subspecies Pterostichus (Sinosteropus) barbarae cavazzutii Sciaky & Facchini, 2003. We therefore propose a replacement name *Pterostichus cavazzutianus* for this species, respecting the authors’ original notion by changing only the suffix.

##### Affinities.

This species seems to be close to *Pterostichus adelphus* sp. n. in their similarities of elytral microsculpture and short apical lamella of aedeagus.

#### Pterostichus (Circinatus) dentifer

Taxon classificationAnimaliaColeopteraCarabidae

Allegro & Sciaky, 2010

Chinese common name: 肩齿通缘步甲 (Jiān Chi Tōng Yuán Bù Jiă)

Figures 22 , 33 , 107

Pterostichus (Circinatus) dentifer Allegro and Sciaky 2010: 4 (Original: *Pterostichus (Circinatus)*; holotype deposited in CRS).

##### Type locality.

Sichuan: Dayi County, Chadiping, 1500 m. Chadiping (N30.61°, E103.17°) is a small village under the south slope of Xilingxueshan Mt.

##### Type material examined.

**Holotype** of *Pterostichus dentifer* Allegro & Sciaky (CRS): male, body length = 14.5 mm, board mounted, genitalia dissected and mounted on plastic film, pinned under specimen, “China, C Sichuan, Dayi distr., / Chadiping env., 1500 m / 5–7.08.1996 / A. Zamotajlov, A. Miroshnikov & / D. Fedorenko”; “ex coll. / A. Zamotajlov”; “HOLOTYPUS ♂ / Pterostichus / (Circinatus)/ dentifer sp. n. / Allegro & Sciaky det. 2010” [red label].

##### Diagnosis.

Pronotum with single mid-lateral seta; posterior seta distant from hind angle, hind angle completely rounded; basal foveal grooves not completely fused, anterior part of outer groove distinctly separated from inner one, so that basal fovea is a little bifid anteriorly; elytron with faint transverse microsculpture; basal ridge strongly oblique lateral-posteriorly; shoulder angle forming obtuse angle, humeral tooth large, strongly pointed; males with terminal sternum not modified; median lobe of aedeagus strongly bent to right side, apical lamella very short, less than one fourth its basal width.

*Pterostichus dentifer* is very similar to *Pterostichus subtilissimus* and *Pterostichus yuxiaodongi* sp. n. They are all relatively large sized species within the subgenus, and have the aedeagus strongly bent to the right side. *Pterostichus dentifer* differs from the latter two species in: (1) elytral humeral tooth much larger; (2) pronotal basal fovea with more punctures; (3) median lobe of aedeagus with shorter apical lamella (than *Pterostichus yuxiaodongi*), and relatively simpler apex (than *Pterostichus subtilissimus*).

##### Supplemental description.

**Male sternum**. Penultimate and terminal sterna of males without special structures. Endophallus not studied, females unknown.

##### Distribution.

So far this species is known only from the holotype from Sichuan Prov., Dayi County, Chadiping, 1500 m. (Map 2)

##### Affinities.

This species is close to *Pterostichus subtilissimus* and *Pterostichus yuxiaodongi* sp. n. For details see discussions under *Pterostichus yuxiaodongi* sp. n. and subtilissimus-group in the section of infra-subgeneric taxonomy.

#### Pterostichus (Circinatus) liciniformis

Taxon classificationAnimaliaColeopteraCarabidae

Csiki, 1930

Chinese common name: 盘胸通缘步甲 (Pán Xīong Tōng Yuán Bù Jiă)

Figures 16 , 42 , 52 , 64 , 80 , 102 , 122

Pterostichus (Circinatus) liciniformis Csiki 1930: 669, replacement name for *Steropus licinoides* Fairmaire, 1888; Jedlička 1962: 298 (*Pterostichus* (*incertae sedis*)); Sciaky 1996: 224; Bousquet 2003: 488; Lorenz 2005: 280; Allegro and Sciaky 2010: 7.
Pterostichus (Circinatus) liciniformis Synonym: *licinoides*Fairmaire 1888: 10, (Original: *Steropus*; syntype deposited in MNHN?). Junior homonym of *Steropus licinoides* Motschulsky, 1866: 261; Tschitschérine 1897: 294 (*Feronia (Pterostichus)*).

##### Type locality.

In the original literature there was no detailed locality indicated, but only “Yunnan” was mentioned. According to our knowledge of the type material of the other Pterostichini species described in the same paper (see Shi et al., 2013: 118 for a detailed discussion) and of the further specimens of *Pterostichus liciniformis* collected in recent years, we infer that the type locality of this species could be in northeast Dali in Yunnan Province.

##### Notes on types.

The syntype was in Fairmaire’s collection, and then should have been transferred to the collection of MNHN. But despite a careful examination of the Carabidae collection in MNHN, we failed to find the type.

##### Non-type material examined

(total 22 specimens). 1 male (CCCC), “China, Lijiang, Laojunshan, 3500 m, 2009.VI.12, ZHU Xiaoyu lgt.”; 3 males, 1 female (IZAS), “China, Yunnan, Lijiang, Yulongxueshan, Yunshanping, in dead log, 2006.11.15, 3200 m, LIU Biao”; 2 males, “China, Yunnan, Lijiang, Yulongxueshan, Yunshanping, in dead log, 2006.11.15, 3200 m, ZHAO Yongshuang”; 2 males (IZAS), “China, Yunnan, Lijiang, Yulongxueshan, Yunshanping, in dead log, 2006.11.15, 3200 m, CHEN Jie”; 2 males (IZAS), “China, Yunnan, Lijiang, Yulongxueshan, Yunshanping, in dead log, 2007.03.14, 3205 m, LIU Biao”; 1 female (IZAS), “Yunnan, Lijiang, Yulongshan, 1979.VIII.10, LIU Guoqing, 2700–3300 m”; 1 male (IZAS), “China, Yunnan Prov., Lijiang county, Laojunshan, grassland, N26.64341 E99.76754, 3510 m, 2007.8.19 day, Liang H.B. collector”; 2 males (IZAS), “Yunnan, Lijiang, Yulongxueshan Mt., Maoniuping, 3271 m, 2012.VI.2, pit fall trap, LIU Ye, SHI Hongliang lgt.”; 1 male (IZAS), “Yunnan, Lijiang, Yulongxueshan Mt., Maoniuping, 3226, 2012.VI.1, LIU Ye, SHI Hongliang lgt.”; 2 males, 1 female (IZAS), “China, Yunnan, Deqen Pref., Zhongdian County, Luoji, pass S. of Jiulong village, 3594 m, N27.67278, E100.02623, 2013.VIII.22, under dead log, SHI Hongliang, LIU Ye & LIU Yizhou lgt.”; 1 female (IZAS), “Yunnan, N. of Zhongdian, 3446 m, 2000.VII.23–30, YU Xiaodong lgt.”; 1 female (IZAS), “Yunnan, Deqin county, E. slope of Baimaxueshan, 3700 m, 1981.VIII.26, LIAO Subai lgt.”; 1 male (NMPC), “China: Yunnan prov., 1.3–2.0, km S of Haba, 17.-20.VI.2007, Haba Xueshan Mts., 2830–, 3000 m, 27°22.1’N 100°08.2’E, J.Hajek & J.Ruzicka leg”; “individually collected on soil surface and on plants and shrubs, sparse mixed forest (with dominant pinus); in/ near the brook”.

##### Diagnosis.

Pronotum with single mid-lateral seta; posterior seta distant from hind angle, hind angle completely rounded; elytral shoulder angle completely rounded, without humeral tooth; male and female with similar faint linear elytral microsculpture; fifth tarsomeres glabrous beneath; males with terminal sternum depressed in middle.

This species differs from all other *Circinatus* species by: body form relatively wide; elytral humeral angle completely rounded; elytral lateral expansion wide and reflexed.

##### Supplemental description.

**Male sternum**. Penultimate sternum without special structures; terminal sternum depressed in middle, depression occupying apical half of sternum, finely rugose inside depression, a faint vertical ridge present in middle of depression, frontal- lateral corners of depression forming two indistinct tubercles (Fig. 122). **Endophallus** (Fig. 42D, E, F) bent to ventral side across left side of aedeagus, major parts of endophallus located on ventral side of aedeagus (in lateral view); gonopore (**gp**) located at level near aedeagal base, pointing to ventral face, gonopore lobe (**gpl**) bent in apical direction. Five distinct lobes recognized: basal lobe (**bl**) close to base of apical lamella, small and rounded; ventral lobe (**vl**) with a long and strongly chitinized piece, rounded membranous lobe borne on ventral-apical part of chitinized piece; right lobe (**rl**) rounded and large, shallowly constricted in middle, decorated with fine scales; left lobe I (**lf-I**) close to base of vl, apex enlarged; left lobe II (**lf-II**) narrower than lf-I, pointing to left side of aedeagus. **Female genitalia**. Spermatheca with seminal canal approx 3.5 times as long as receptaculum; receptaculum clavate (Fig. 64), gradually expanded to apex; seminal canal inserted at base of common oviduct, base of seminal canal sclerotized. Stylomere II with two ensiform setae at basal half of outer margin, and one near middle of inner margin; two short nematiform setae located in a furrow near apex. Female sternum VIII (Fig. 80B) with fine setae on posterior margin; posterior margin almost straight, slightly notched in middle; anterior region more weakly chitinized than posterior region, without denser pigmentation, deeply notched in middle; middle transparent region diamond-shaped, with two very narrow oblique extensions adjacent to anterior and posterior notches in middle. Female tergum VIII (Fig. 80A) very short, mainly semi-chitinized with sparse and irregular spots; lateral-anterior region with two chitinized patches; anterior margin not notched in middle, posterior margin evenly arcuate.

##### Distribution.

This species is relatively widely distributed in northwest Yunnan. So far it has been recorded from several localities in Lijiang, Shangri-La (=Zhongdian), and Deqin (Map 1).

##### Affinities.

As indicated by Allegro and Sciaky (2010: 7), *Pterostichus liciniformis* appears to be isolated in the species group. This species could be close to the other two species from north Yunnan, *Pterostichus wangjiani* sp. n. and *Pterostichus dimorphus* sp. n. Despite differences in the pronotal hind angle, elytral shoulder angle and microsculpture, these species are very similar in the depressed male terminal sternum and aedeagus. Nevertheless, *Pterostichus liciniformis* could represent an early branch in the *baenningeri*-group. A detailed discussion is provided below.

##### Geographical variation.

We studied males from two localities, Lijiang (Yulongxueshan Mt.; N27.20°, E100.27°) and Shangri-La (= Zhongdian; pass S. of Jiulong village; N27.67°, E100.03°). The specimens from these two localities have no differences in the external or aedeagal (endophallus excluded) characters. However, for the endophallus, we found one difference: in a specimen from Lijiang, the apex of lf-II is distinctly enlarged in dorsal view; in the specimen from Shangri-La, lf-II is smaller than that from Lijiang, and the apex less enlarged in ventral view. (Figs 42, 52)

#### Pterostichus (Circinatus) pohnerti

Taxon classificationAnimaliaColeopteraCarabidae

Jedlička, 1934

Chinese common name: 波纳通缘步甲 (Bō Nà Tōng Yuán Bù Jiă)

Figures 26 , 47 , 56 , 85 , 111

Pterostichus (Circinatus) pohnerti Jedlička 1934: 19 (Original: *Pterostichus* (*incertae sedis*); holotype deposited in NMPC); Jedlička 1962: 298 (*Pterostichus* (*incertae sedis*)); Sciaky 1996: 221; Bousquet 2003: 488; Lorenz 2005: 280; Allegro and Sciaky 2010: 7.

##### Type locality.

The original literature mentioned the locality “Tatsienlu”, without further details. But the label of the holotype indicates that the type locality is “Kiulung” (= Jiulong), a county west of Gonggashan Mountain.

##### Type material examined.

**Holotype** of *Pterostichus pohnerti* Jedlička (NMPC), male, body length = 10.9 mm, board mounted, with left foreleg, right hind tarsomeres (apical four segments), left antenna (apical ten antennomeres) and right antenna (apical six antennomeres) missing, genitalia dissected and stored in micro vial pinned under specimen, “Tatsienlu-Kiulung / China Em. Reitter”; ”TYPE” [red label]; ”Mus. Nat. Prague / Inv. *22407*” [orange label]; ”*Pohnerti* / *type*, *sp. n.*, / DET. ING. JEDLIČKA” [pink label].

##### Non-type material examined

(66 specimens). 3 males, 4 females (IZAS), “China, Sichuan, Luding county, Moxi town, way btw. Moxi and Yajiageng pass, mixed forest, 3204 m, N 29.84110, E 102.04802, by pit fall trap, 2012.VII.11, SHI Hongliang & WANG Ya’nan lgt.”; 1 male, 3 females (IZAS), “Sichuan, Luding, Moxi, 1990.VII.6, T. Deuve & XIE Weiping lgt.”; 1 male (IZAS), “Sichuan, Luding, Hailuogou, 3000 m, 2004.VIII.8, BAI Ming lgt.”; 4 males, 5 females (IZAS), “China, Sichuan, Luding, Hailuogou, 3200, 2011.7.16, Huang Hao”; 13 males, 5 females (IZAS), “China, Sichuan Prov., Luding, Hailuogou, Caohaizi, N29.58228, E102.02171, 2775 m, 2009.V.18, Liang H.B. lgt.”; 1 male, 8 females (IZAS), “China, Sichuan Prov., Luding, Hailuogou, Guanjingtai, N29.56721, E101.97994, 3155 m, 2009.V.16, Yang G.Y. lgt.”; 2 males (IZAS), “China, Sichuan, Tianquan, Erlangshan, 2500 m, 2011.VIII.5, Huang Hao lgt.”; 9 males, 7 females (IZAS), “China, Sichuan, Baoxing county, Jiajinshan mt., Mahuanggou, mixed forest, N30.85669, E102.76615, 2012.VI.29 D 2681 m, Liu Ye, Shi Hongliang leg.”.

##### Diagnosis.

Pronotum with single mid-lateral seta; posterior seta distant from hind angle, hind angle completely rounded; outer basal foveal groove distinct, approx half length of inner one, forming a short ridge close to hind angle; basal fovea area finely punctate; elytron with sharp humeral tooth; elytral with faint linear microsculpture; fifth tarsomeres glabrous beneath; terminal sternum of male not modified.

This species is very similar to *Pterostichus xilingensis*. They are difficult to separate by external appearance (the only difference is the punctures in the pronotal basal fovea (see the key), but this does not always distinguish them), but they can be easily determined by their allopatric

##### Distribution.

In male and female genitalia, these two species are different. In *Pterostichus pohnerti*: median lobe of aedeagus stouter; apical lamella triangular, strongly narrowed to apex; endophallus extending in apical direction of aedeagus; female transparent region on sternum VIII V-shaped, with very long and narrow extensions. In *Pterostichus xilingensis*: median lobe of aedeagus much slenderer; apical lamella wide, slightly narrowed to apex; endophallus extending in apical-dorsal direction of aedeagus; female transparent region on sternum VIII nearly quadrate, with very short extensions.

##### Supplemental description.

**Male sternum**. Penultimate and terminal sterna without special structures. **Endophallus** (Fig. 47D, E, G) short and straight, extending in apical direction of aedeagus, major portion of endophallus located beyond apical lamella; gonopore (**gp**) located at level near aedeagal apex, pointing to ventral face of aedeagus, gonopore lobe (**gpl**, fully everted in Fig. 47) bent in basal direction of aedeagus. Four distinct parts recognized: basal lobe (**bl**) close to base of apical lamella, very small, approx triangular; main piece (**mp**) large and galericulate, with three branches, covering gpl, apical opening of gpl pointing out from ventral notch between mp branches; apical band (**ab**) close to gp, also covered by mp, half chitinized, and the rest half scaled and extended to inner margin of pa; pre-apical lobe (**pa**) small and compressed, ear-like, with fine and dense scales. **Female genitalia**. Spermatheca (Fig. 56) with seminal canal approx 4.5 times as long as receptaculum; receptaculum capitate, club approx one third as long as receptaculum; seminal canal inserted at base of common oviduct, base of seminal canal with sclerotized region long. Stylomere II with two ensiform setae at basal half of outer margin, and a small one near middle of inner margin; two short nematiform setae located in a furrow near apex. Female sternum VIII (Fig. 85B) with fine and dense setae on posterior margin; posterior margin almost straight, strongly notched in middle; anterior region semi-chitinized, deeply notched in middle; middle transparent region V-shaped, adjacent to anterior and posterior notches in middle. Female tergum VIII (Fig. 85A) short, major portion chitinized, apical fourth semi-chitinized, with very sparse spots; anterior margin weakly notched in middle, posterior margin evenly arcuate.

##### Distribution.

*Pterostichus pohnerti* is recorded from four counties of central Sichuan province: Luding (Gonggashan range), Jiulong (type locality), Baoxing (south slope of Jiajinshan), and Tianquan (east slope of Erlangshan). This species is very common and the only known *Circinatus* species in the Gonggashan range. A large number of specimens was collected from several localities around this mountain. Outside the Gonggashan range, it is also distributed in Baoxing and Tianquan, where it overlaps the range of *Pterostichus beneshi*. (Maps 2, 3)

##### Affinities.

*Pterostichus pohnerti* could be closest to *Pterostichus xilingensis* based on the similarity in external characters. However, in the stout aedeagus and endophallus extending to the aedeagal apex, *Pterostichus pohnerti* could be allied with some species in the *agilis*-group. Moreover, *Pterostichus pohnerti* has the southernmost distribution in the *pohnerti*-group, which is the closest distribution to the range of the *agilis*-group. Based on these distributions, we infer that the systematic position of *Pterostichus pohnerti* could be between *Pterostichus xilingensis* and the *agilis*-group.

##### Geographical variation.

We studied males from three localities: Hailuogou (N29.58°, E102.02°), Erlangshan (N29.88°, E102.30°), and Baoxing (N30.86°, E102.77°), and found no differences in external, aedeagal or endophallic characters.

#### Pterostichus (Circinatus) subtilissimus

Taxon classificationAnimaliaColeopteraCarabidae

Sciaky, 1996

Chinese common name: 修通缘步甲 (Xīu Tōng Yuán Bù Jiă)

Figures 21 , 45 , 67 , 84 , 106

Pterostichus (Circinatus) subtilissimus Sciaky 1996: 227 (Original: *Pterostichus (Circinatus)*; holotype deposited in CRS); Bousquet 2003: 488; Lorenz 2005: 280; Allegro and Sciaky 2010: 4.

##### Type locality.

Sichuan: Emei Shan Mt. (= Emei Mountain), 2800 m.

##### Type material examined.

**Holotype** of *Pterostichus subtilissimus* Sciaky (CRS): male, body length = 14.8 mm, board mounted, genitalia dissected and mounted on plastic film, pinned under specimen, “Cina- W Sichuan / Emei Shan 2800 m. / 25–31.VI.92. Sauer”; “HOLOTYPUS ♂ / Pterostichus / (Circinatus)/ subtilissimus sp. n. / Det. Sciaky, 1994” [red label].

##### Non-type material examined

(total 4 specimens). 1 male (IZAS), “China, Sichuan Prov., Mount Emei, Leidongping; 2420 m, N29.54669, E103.33552, 2012.VII.02, in dead log, SHI Hongliang & LIU Ye lgt.”; 1 female (IZAS), “China, Sichuan Prov., Mount Emei, Leidongping; 2420 m, N29.54669, E103.33552, 2012.VII.02 night, on tree trunk, SHI Hongliang & LIU Ye lgt.”; 1 male, 1 female (IZAS), “China, Sichuan Prov., Mount Emei, Leidongping; 2420 m, N29.54669, E103.33552, 2012.VII.06, by pit fall trap, SHI Hongliang & LIU Ye lgt.”.

##### Diagnosis.

Pronotum with single mid-lateral seta; posterior seta distant from hind angle, hind angle completely rounded; basal foveal grooves incompletely fused, anterior part of outer groove distinctly separated from inner one, so that basal fovea is a little bifid anteriorly; elytron with faint transverse microsculpture; basal ridge almost horizontal; shoulder angle forming obtuse angle, humeral tooth distinct, short and obtuse; males with terminal sternum not modified; median lobe of aedeagus strongly bent to right side, strongly constricted at middle of apical orifice, apical lamella very short, less than one fourth its basal width.

*Pterostichus subtilissimus* is very similar to *Pterostichus dentifer* and *Pterostichus yuxiaodongi* sp. n. They are all relatively large-sized species within the subgenus, and have the aedeagus strongly bent to the right side. *Pterostichus subtilissimus* differs from the latter two species by: (1) elytral basal ridge almost horizontal, humeral tooth pointing laterally (basal ridge strongly oblique, humeral tooth pointing lateral-posteriorly in the latter two species); (2) body form slenderer, elytron narrower than the latter two species; (3) median lobe of aedeagus strongly constricted in the middle part of apical orifice (not constricted in the latter two species).

##### Supplemental description.

**Male sternum**. Penultimate and terminal sterna of males without special structures. **Endophallus** (Fig. 45D, E, G) completely membranous, without any chitinized piece or heavy scale; bent to dorsal side of aedeagus, all parts of endophallus located on dorsal side of aedeagus; in dorsal view, endophallus rolled counter-clockwise a full circle, gonopore (**gp**, gonopore lobe folded in Fig. 45) located close to left margin of apical orifice, pointing to aedeagal apex, region around gp finely pigmented with spots; two major lobes recognized: basal lobe (**bl**) large and rounded, located at level of apical lamella; middle lobe (**ml**) narrower and longer than bl, apex finely stained, located on right margin of apical orifice, just supporting constriction on right face of aedeagus (Fig. 45D). **Female genitalia**. Spermatheca with seminal canal approx 4.5 times as long as receptaculum; receptaculum capitate (Fig. 67), club approx half length of receptaculum; seminal canal inserted at base of common oviduct, base of seminal canal sclerotized. Stylomere II with two ensiform setae at outer margin, and one near middle of inner margin; two very short nematiform seta located in a furrow near apex. Female sternum VIII (Fig. 84B) with fine and sparse spines on posterior margin; posterior margin slightly curved, strongly notched in middle; posterior region chitinized; anterior region semi-chitinized, deeply and widely notched in middle; middle transparent region V-shaped, adjacent to anterior and posterior notches, oblique extensions of V-shaped transparent region with sparse spots. Female tergum VIII (Fig. 84A) well chitinized, a narrow area close to posterior margin lighter, with irregular small spots; anterior margin deeply notched in middle.

##### Distribution.

So far this species is known only from the type locality, Emei mountain in Sichuan province. The altitude range is between 2420–2800 m. (Map 2)

##### Affinities.

This species is close to *Pterostichus dentifer* and *Pterostichus yuxiaodongi* sp. n. For details see discussions under *Pterostichus yuxiaodongi* sp. n. and *subtilissimus*-group.

#### Pterostichus (Circinatus) xilingensis

Taxon classificationAnimaliaColeopteraCarabidae

Allegro & Sciaky, 2010

Chinese common name: 西岭通缘步甲 (Xī Ling Tōng Yuán Bù Jiă)

Figures 27 , 48 , 70 , 86 , 112

Pterostichus (Circinatus) xilingensis Allegro and Sciaky 2010: 13 (Original: *Pterostichus (Circinatus)*; holotype deposited in CRS).

##### Type locality.

Xiling snow Mts. (= Xilingxueshan Mt.), 2100–3100 m. Xilingxueshan (~N30.70°, E103.16°) is a mountain in Dayi County, Sichuan province.

##### Type material examined.

**Holotype** of *Pterostichus xilingensis* Allegro & Sciaky (CRS), male, body length = 10.5 mm, board mounted, genitalia dissected and glued on board pinned under specimen, “CHINA C Sichuan / Xiling Snow Mts. / 2100–3100 m / 1–3.viii.1996 / leg. S. Kasantsev”; “HOLOTYPUS ♂ / Pterostichus / (Circinatus)/ xilingensis sp. n. / Allegro & Sciaky det. 2010” [red label].

##### Non-type material examined

(total 11 specimens). 4 males, 1 female (IZAS), “Sichuan, Wolong, Wuyipeng, 2525–2710 m, 2004.VI.29–VII.29, YU Xiaodong lgt.”; 2 males, “Sichuan, Wolong, Wuyipeng, Erdaoping, 3045–3055 m, 2004.VI.15–30, YU Xiaodong lgt.”; 1 male, 1 female (IZAS), “Sichuan, Wolong, Wuyipeng, Erdaoping, 2965–3045 m, 2004.VII.30–VIII.15, YU Xiaodong lgt.”; 1 male (IZAS), “Sichuan, Wolong, Wuyipeng, Erdaoping, 2945 m, 2004.V.30–VI.2, YU Xiaodong lgt.”; 1 male (IZAS), “Sichuan, Baoxing, Guobayan, 2880 m, pitfall trap, 2001.VII.1–4, YU Xiaodong, ZHOU Hongzhang lgt.”.

##### Diagnosis.

Pronotum with single mid-lateral seta; posterior seta distant from hind angle, hind angle completely rounded; outer basal foveal groove distinct, approx half length of inner one, forming a short ridge between it and lateral margin; basal fovea area almost impunctate; elytron with sharp humeral tooth; elytral microsculpture faint, linear; fifth tarsomeres glabrous beneath; terminal sternum of male not modified. This species is very similar to *Pterostichus pohnerti*. Their comparison has been provided under *Pterostichus pohnerti*.

##### Supplemental description.

**Male sternum**. Penultimate and terminal sterna without special structures. **Endophallus** (Fig. 48D, E, F) short, extending in dorsal-right direction of aedeagus, major portion of endophallus located on dorsal side of aedeagus; gonopore (**gp**, gonopore lobe folded in Fig. 48) located at level near apical lamella apex, pointing to right, with very fine scales around gp. Two distinct parts recognized: basal lobe absent; dorsal lobe (**dl**) large and coniform, pointing to dorsal face of aedeagus, apex strongly chitinized, with scales; ventral lobe (**vl**) with a strongly chitinized piece, narrow and long, pointing to ventral face of aedeagus, apical half with a membranous region on inner surface, apex with fine scales. **Female genitalia**. Spermatheca with seminal canal approx four times as long as receptaculum; receptaculum capitate (Fig. 70), club approx one third length of receptaculum; seminal canal inserted at base of common oviduct, base of seminal canal with distinct sclerotized region. Stylomere II with two ensiform setae at basal half of outer margin, and a small one near middle of inner margin; two short nematiform setae located in a furrow near apex. Female sternum VIII (Fig. 86B) with fine and dense setae on posterior margin; posterior margin slightly curved, narrowly notched in middle; posterior region well chitinized; anterior region semi-chitinized, deeply notched in middle; middle transparent region small, approx quadrate, with short extensions, middle transparent region adjacent to anterior and posterior notches in middle. Female tergum VIII (Fig. 86A) short, most of region semi-chitinized, with sparse and irregular spots; only lateral-anterior region with two chitinized patches; anterior margin not notched in middle, posterior margin evenly arcuate.

##### Distribution.

Sichuan (Xilingxueshan Mt., Wolong and Baoxing). In Xilingxueshan and Wolong, *Pterostichus xilingensis* is sympatric with *Pterostichus beneshi*. In Wolong, it seems less common than *Pterostichus beneshi*. *Pterostichus xilingensis* seems to be strictly allopatric with *Pterostichus pohnerti*, although their localities in Baoxing County are only approx 30km apart in linear distance. These two species could be isolated by different altitude in Baoxing (Maps 2, 3).

##### Affinities.

The affinity between *Pterostichus xilingensis* and *Pterostichus pohnerti* was discussed under *Pterostichus pohnerti*. The systematic position of *Pterostichus xilingensis* could be between *Pterostichus pohnerti* and (*Pterostichus zoiai* + *Pterostichus beneshi*).

##### Geographical variation.

*Pterostichus xilingensis* is recorded from three very close localities. No significant difference was found between specimens from each locality, but only the endophallus of specimens from Wolong were examined.

#### Pterostichus (Circinatus) zoiai

Taxon classificationAnimaliaColeopteraCarabidae

Sciaky, 1996

Chinese common name: 佐亚通缘步甲 (Zuŏ Yà Tōng Yuán Bù Jiă)

Figures 25 , 49 , 69 , 88 , 110

Pterostichus (Circinatus) zoiai Sciaky 1996: 222 (Original: *Pterostichus (Circinatus)*; holotype deposited in NHMB); Bousquet 2003: 488; Lorenz 2005: 280; Allegro and Sciaky 2010: 4.

##### Type locality.

Sichuan: Emei Mt. (~N29.55°, E103.34°), 600–1050 m.

##### Type material examined.

**Holotype** of *Pterostichus zoiai* Sciaky (NHMB), male, body length = 10.7 mm, board mounted, genitalia dissected and glued on same board with specimen, “CHINA: Sichuan / Mt. EMEI, 600–1050 m / 5.-19.5.1989 / Lad. Bocák, lgt.”; “*HOLOTYPUS* / *Pterostichus* / (*Circinatus*)/ *zoiai* *sp. n.* / *Det. Sciaky ‘94*” [red label]. **Paratype**, 1 male, “CHINA, Sichuan, / Mt. Emei, 2640 m, 6.5.1989, Lad. Bocak, lgt.”; “ex. coll. R. Sciaky, 2011”.

##### Non-type material examined

(total 44 specimens). 9 males, 6 females (IZAS), “China, Sichuan, mount. Emei, Leidongping, broadleaf forest with dead log under forest, N29.54669, E103.33552; 2420 m, 2012.VII.02 N, on ground or tree trunk, SHI Hongliang, LIU Ye, leg.”; 2 males, 2 females (IZAS), same data, but collecting date 2012.VII.03; 2 males, 7 females (IZAS), same data, but collecting date 2012.VII.05; 7 males, 8 females (IZAS), “China, Sichuan, mount. Emei, Leidongping, broadleaf forest with dead log under forest, N29.54669, E103.33552; 2420 m, 2012.VII.06, by pitfall trap, SHI Hongliang, LIU Ye, leg.”; 1 female (IZAS), “Mt. Emei, Sichuan Prov. 17-VII-2003, Hu & Tang leg.”.

##### Diagnosis.

Pronotum with single mid-lateral seta; posterior seta distant from hind angle, hind angle completely rounded; basal foveal outer groove absent, outer region of inner groove completely flat; basal fovea area finely punctate between two inner grooves; elytron with humeral tooth almost invisible; elytral microsculpture faint, linear; fifth tarsomeres glabrous beneath; terminal sternum of male not modified. This species is very similar to *Pterostichus beneshi*. Their comparison has been provided in the diagnosis under *Pterostichus beneshi*.

##### Supplemental description.

**Male sternum**. Penultimate and terminal sterna without special structures. **Endophallus** (Fig. 49D, E, G) extended in dorsal-basal direction of aedeagus, major portion of endophallus located on dorsal side of aedeagus; gonopore (**gp**, gonopore lobe folded in Fig. 49) located at level near middle of aedeagus, pointing to right. Five distinct lobes recognized: left lobe (**ll**) largest, located on left face near gp, its apex branched, forming two rounded sub-lobes, upper half of ll decorated with scales, scales near apex of ll very coarse; ventral lobe I (**vl-I**) tubiform, pointing to dorsal side of aedeagus, apex a little enlarged; ventral lobe II (**vl-II**) much smaller than vl-I; right lobe (**rl**) longer and thicker than vl-II, tubiform, straight; pre-apical lobe (**pa**) located on right face, close to gp, apex enlarged and branched, with very fine scales; basal-dorsal region on endophallus heavily pigmented; endophallus without chitinized piece. **Female genitalia**. Spermatheca with seminal canal approx three times as long as receptaculum; receptaculum capitate (Fig. 69), club approx half length of receptaculum; seminal canal inserted at base of common oviduct, base of seminal canal with sclerotized region short and thick. Stylomere II with two ensiform setae at basal half of outer margin, and a small one near middle of inner margin; two short nematiform setae located in a furrow near apex. Female sternum VIII (Fig. 88B) with fine setae on posterior margin, setae on middle region a little thicker; posterior margin almost straight, notched in middle; posterior region well chitinized; anterior region semi-chitinized, deeply notched in middle; middle transparent region small, roughly diamond-shaped, with two short oblique extensions; middle transparent region adjacent to anterior and posterior notches in middle. Female tergum VIII (Fig. 88A) short, posterior region semi-chitinized, without denser pigmentation; anterior region evenly chitinized; anterior margin notched in middle, posterior margin evenly arcuate.

##### Distribution.

Known only from Emei mountain in central Sichuan province (Map 2). The label of the holotype indicates that this species is from low altitude (600–1050 m) on Emei Mt. As a result of our expedition to Emei Mt. in 2012, we found that this species is very common at relatively high altitudes (more than 2000 m) of Emei Mountain.

##### Affinities.

This species is close to *Pterostichus beneshi* in similarities of external appearance and male genitalia. Their endophalli are also similar, all lobes being recognizable as homologous.

### Species removed from *Circinatus*

There are two species, *Pterostichus schuelkei* Sciaky & Wrase, 1997, and *Pterostichus wenxianensis* Allegro & Sciaky, 2010, described in subgenus *Circinatus*, which are in the present study moved to subgenus *Morphohaptoderus*.

#### Pterostichus (Morphohaptoderus) schuelkei

Taxon classificationAnimaliaColeopteraCarabidae

Sciaky & Wrase, 1997 new subgeneric assignment

Figures 89 , 126 , 137

Pterostichus (Morphohaptoderus) schuelkei Sciaky and Wrase 1999: 1102 (Original: *Pterostichus (Circinatus)*; holotype deposited in CDW); Bousquet 2003: 488; Lorenz 2005: 280 (*Pterostichus (Circinatus)*); Allegro and Sciaky 2010: 14 (*Pterostichus (Circinatus)*).

##### Type material examined.

**Holotype** of *Pterostichus schuelkei* Sciaky & Wrase (CDW), female, body length = 10.8 mm, board mounted, “CHINA (Shaanxi) / Qin Ling Shan / 108.47 E / 33.51 N / Mt. W pass / autoroute km 70,47km. S Xian 2350–2500 m / 7.–19.VII.1996 / Kleinfeld & Schutze”; “COLL.WRASE / BERLIN”; “HOLOTYPUS / Pterostichus / (Circinatus)/ schuelkei sp. n. / Sciaky & Wrase des. 1997” [red label].

##### Non-type material examined

(total 20 specimens). 12 females (IZAS), “CHINA, Shaanxi Prov., Ningshan, Huoditang, pitfall trap, 33.43368N 108.44747E, 1538 m, 2007.08.20, Shi H.L. Yang G.Y. coll.”; 1 male, 4 females (IZAS), “China, Shaanxi Prov., Zhouzhi, Houzhenzi, Laoxiancheng, pitfall trap, 33.79211N 107.74421E, 1860 m, 2007.08.14, Shi Hongliang collector”; 1 female (IZAS), “Shaanxi, Ningshan county, Huoditang, 1580 m, 1998.VII.27, Chen Jun leg.”; 1 male (IZAS), “Shaanxi, Foping county, Liangfengya, 2150–1750 m, 1999.VI.28, Yao Jian leg.”; 1 male (IZAS), “Shaanxi, Zhouzhi county, Houzhenzi W. 3km, 2008.V.11, Huang Hao leg.”.

##### Diagnosis.

Third antennomere without secondary setae; submentum with one seta on each side; pronotal hind angle completely rounded; posterior seta close to hind angle; basal foveal area finely punctate, outer groove short and superficial; third interval of elytra with two setigerous pores, all adjacent to second stria; metacoxa with three setae; fifth tarsomeres setose beneath; males with terminal and penultimate sterna unmodified. Median lobe of aedeagus carinate ventrally; apical lamella simple, pointing apical-ventrally; apical orifice opened dorsally; right paramere short, length approx 1.6 times greatest width, apical portion completely rounded.

##### Distribution.

This species was recorded from several localities (within 100 km of each other) in Qinling mountain (Shaanxi province).

##### Discussion.

*Pterostichus schuelkei* was originally placed in the subgenus *Circinatus* because of its typical *Circinatus*-like pronotum, namely, the almost rounded hind angle. However, after a careful morphological study, we found that *Pterostichus schuelkei* differs from the other members of *Circinatus* in some important characters, which leads us to move it from *Circinatus* to *Morphohaptoderus*. In the present study, we emphasize the following two characters: (1) number of setae on the metacoxa, and (2) shape of the right paramere of the aedeagus.

*Pterostichus schuelkei* has three setae on the metacoxa (Fig. 137), but in the true *Circinatus* species, only two are present. In most *Pterostichus* species, the metacoxa has only two setae, one close to the outer-posterior angle and the other seta close to anterior margin of the metacoxa (1 and 2 in Fig. 137). In some *Pterostichus* subgenera, one additional seta is present near the inner angle of the metacoxa (3 in Fig. 137), which is typically finer and shorter than the other two setae. The number of setae on the metacoxa is important in the subgeneric classification of the genus *Pterostichus*. In the Chinese fauna of *Pterostichus* (35 total subgenera recorded according to our unpublished results), only three subgenera have three setae present on the metacoxa: *Cryobius*, *Morphohaptoderus*, and *Tschitscherinea*. These three subgenera are closely allied to one another, and all have similar body forms and pronotal basal foveae.

The basal plan of the right paramere in the subgenus *Circinatus* is straight and fusiform such as in *Pterostichus baenningeri* (Fig. 40C), which is modified to be stouter or triangular in some species such as *Pterostichus camelus* sp. n. (Fig. 28C). The ratio of the right paramere length to its greatest width is between 2.5 and 4.0 in *Circinatus*. However, in *Pterostichus schuelkei*, the right paramere is much shorter (length/width = 1.6) and the apical portion is approximately round (Fig. 126D), which is consistent with the typical form in the subgenus *Morphohaptoderus*. In most species of *Morphohaptoderus*, the right parameres are almost identical, with the apical portions very short and approximately round; there are only two exceptions in the described species (*Pterostichus dundai* and *Pterostichus muellermotzfeldi*). In *Pterostichus dundai*, the apical portion of the right paramere is strongly elongated and bent, and in *Pterostichus muellermotzfeldi*, the right paramere is approximately fusiform. Moreover, this type of short and round right paramere is present only in the subgenus *Morphohaptoderus*.

*Pterostichus schuelkei* is surely the closest species to *Pterostichus wenxianensis*, as indicated by Allegro & Sciaky (2010). When sorting for *Morphohaptoderus* specimens in the collection of the IZAS, we found that two other *Morphohaptoderus* species could also be related to *Pterostichus schuelkei* and *Pterostichus wenxianensis*, which are *Pterostichus janatai* Sciaky & Wrase and *Pterostichus hubeicus* Facchini & Sciaky. All four of these species are found in or close to the Qinling Mountain range in central China. They share the following characters and may form a species group in subgenus *Morphohaptoderus*: (1) submentum with only one seta on each side; (2) third interval with two (occasionally three) setigerous pores, all adjacent to the second stria; (3) median lobe of the aedeagus carinate ventrally; and (4) apical lamella of the aedeagus simple, pointing apical-ventrally, and not hooked or twisted. The combination of these four characters is unique to the subgenus *Morphohaptoderus* and may support a relationship among these four species, although *Pterostichus janatai* and *Pterostichus hubeicus* differ from the other two because of a distinct pronotal hind angle.

Based on all considerations, *Pterostichus schuelkei* is assigned to the subgenus *Morphohaptoderus* in the present paper.

#### Pterostichus (Morphohaptoderus) wenxianensis

Taxon classificationAnimaliaColeopteraCarabidae

Allegro & Sciaky, 2010 new subgeneric assignment

Figures 113 , 127

Pterostichus (Morphohaptoderus) wenxianensis Allegro and Sciaky 2010: 15 (Original: *Pterostichus (Circinatus)*; holotype deposited in CRS).

##### Type material examined.

**Holotype** of *Pterostichus wenxianensis* Allegro & Sciaky (CRS), female, body length = 12.4 mm, board mounted, “*GANSU* / *Wenxian*”; “HOLOTYPUS ♂/ Pterostichus / (Circinatus)/ wenxianensis sp. n. / Allegro & Sciaky det. 2010” [red label].

##### Diagnosis.

This species is very similar to *Pterostichus schuelkei*, but differs from the latter by: (1) hind angle forming indistinct obtuse angle (completely rounded in *Pterostichus schuelkei*); (2) body larger and wider than *Pterostichus schuelkei*; (3) ventral carina of median lobe of aedeagus stronger and longer than in *Pterostichus schuelkei*.

##### Distribution.

Known only from the holotype, collected in Wenxian, Gansu, without further detailed locality.

##### Discussion.

This species is very close to *Pterostichus schuelkei*. We assign it to *Morphohaptoderus* for the same reason as *Pterostichus schuelkei*.

### Infra-subgeneric taxonomy

Allegro and Sciaky (2010) revised and divided the subgenus *Circinatus* into three species groups. However, their “group of *Pterostichus schuelkei*”, which included two species, actually did not belong in the subgenus *Circinatus*, as discussed above. The rest of the eleven species were divided into two species groups based primarily on male genitalia characters, namely, the length of the apical lamella and the presence of chitinized structures in the folded endophallus.

In the present study, we found that the endophallic characters have important value in the infra-subgeneric taxonomy of *Circinatus*. The endophalli of the 22 known species (the endophallus studied in 16 species) in the subgenus are classified into three types: **Type I** (Fig. 34) has the endophallus bent to the ventral side of the aedeagus across the apex of the apical lamella, with the major portion of endophallus located on the ventral side of aedeagus, and the gonopore oriented to the aedeagus base. All species of the *agilis*-group have endophallus type I. **Type II** (Fig. 40) has the apical orifice opened on the ventral face of the median lobe, with the endophallus bent to the ventral side of the aedeagus from the ventral opening (Figs 133, 134), the major portion of the endophallus located on the ventral side of the aedeagus, and the gonopore typically oriented to the aedeagus apex. All species of the *baenningeri*-group have endophallus type II. **Type III** (Fig. 50) has the endophallus bent to the dorsal side of the aedeagus, with the major portion of the endophallus located on the dorsal side of the aedeagus, and the gonopore orientation variable. All species of the *subtilissimus*-group and the two species of the *pohnerti*-group (*Pterostichus beneshi* and *Pterostichus zoiai*) have endophallus type III. The other two species of the *pohnerti*-group (*Pterostichus pohnerti* and *Pterostichus xilingensis*) have endophallus types intermediate between types I and III; *Pterostichus xilingensis* is closer to type III, and *Pterostichus pohnerti* is closer to type I. The character evolution of the endophallus types is discussed in the section on phylogenetic analyses and is illustrated in Fig. 3.

In addition to the endophallus, the following external or genital characters are also emphasized: (1) the number of pronotal mid-lateral setae; (2) the pronotal hind angle shape and position of the posterior seta; (3) elytral microsculpture; (4) modification of the male terminal and penultimate sterna; and (5) the shape of the median lobe of the aedeagus, including the apical orifice opening and the apical lamella shape and orientation.

Three types of characters are defined in the discussion of the morphology of each species group. **Definitive characters** of a species group are those characters that are present in all members of the species group but that are not present in any other species group. In general, definitive characters define a species group. **Exclusive characters** of a species group are those characters that are present only in some members of the species group and that are not present in any other species group. **Common characters** of a species group are those characters that are present in all members of the species group but are also present in some members of other species groups. The characters that occur in all species of the subgenus are not considered common characters of a species group.

As a result of the analysis of the morphological characters, the following four species groups in the subgenus *Circinatus* are recognized. Morphological characters, in addition to their geographical distributions, support each species group. (Maps 1, 2)

### Checklist of the subgenus *Circinatus* Sciaky

***agilis*-group**

Pterostichus (Circinatus) adelphus sp. n.

Pterostichus (Circinatus) agilis Allegro & Sciaky, 2010

Pterostichus (Circinatus) bullatus Allegro & Sciaky, 2010

Pterostichus (Circinatus) camelus sp. n.

Pterostichus (Circinatus) cavazzutianus cavazzutianus replacement name

Pterostichus (Circinatus) cavazzutianus mianningensis ssp. n.

Pterostichus (Circinatus) zhygealu sp. n.

***baenningeri*-group**

Pterostichus (Circinatus) ailaoicus sp. n.

Pterostichus (Circinatus) baenningeri Jedlička, 1931

Pterostichus (Circinatus) dimorphus sp. n.

Pterostichus (Circinatus) liciniformis Csiki, 1930

Pterostichus (Circinatus) maitreya sp. n.

Pterostichus (Circinatus) miao sp. n.

Pterostichus (Circinatus) tumulus sp. n.

Pterostichus (Circinatus) wangjiani sp. n.

Pterostichus (Circinatus) yan sp. n.

***subtilissimus*-group**

Pterostichus (Circinatus) dentifer Allegro & Sciaky, 2010

Pterostichus (Circinatus) subtilissimus Sciaky, 1996

Pterostichus (Circinatus) yuxiaodongi sp. n.

***pohnerti*-group**

Pterostichus (Circinatus) beneshi Sciaky, 1996

Pterostichus (Circinatus) pohnerti Jedlička, 1934

Pterostichus (Circinatus) xilingensis Allegro & Sciaky, 2010

Pterostichus (Circinatus) zoiai Sciaky, 1996

### Key to species groups of subgenus *Circinatus*

**Table:** 

1	Pronotum with two or more mid-lateral setae; male endophallus as type I	***agilis*-group**
–	Pronotum with only one mid-lateral seta; male endophallus not as type I	**2**
2	Male terminal sternum modified; male endophallus as type II; apical orifice opened ventrally	***baenningeri*-group**
–	Male terminal sternum not modified; male endophallus as or close to type III; apical orifice not opened ventrally	**3**
3	Elytral microsculpture transverse or isodiametric; apex of male median lobe strongly bent to right; body length more than 14.4 mm	***subtilissimus*-group**
–	Elytral microsculpture linear; apex of male median lobe not bent to right; body length less than 12.5 mm	***pohnerti*-group**

### 1) *agilis*-group

**Geographical distribution.** This species group contains six species and one subspecies, including two previously described species, four new species and one new subspecies described in the present work. The distributions of all members of the group are in southern Sichuan. The range of the *agilis*-group is approximately the same as the range of the Liangshan Yi Autonomous Prefecture (Map 2). Because the carabid fauna of Liangshan has been poorly studied, the discovery of more new species is expected on future expeditions. The range of the *agilis*-group does not overlap with any other species group.

**Endophallus**. In all species of the *agilis*-group, the endophallus is bent to the ventral side across the apical lamella, the major portion of the endophallus is located on the ventral side of the median lobe, and the gonopore is oriented to the aedeagal base (endophallus type I). The basal plan of the endophallus in the *agilis*-group has four lobes (Figs 35–39): the ventral-basal lobe I (vb-I) is small, more or less compressed, with the outer face chitinized, and is close to the left side of the apical lamella base; the ventral-basal lobe II (vb-II) is large and prolonged and contains a strongly chitinized piece and a membranous region; the ventral-apical lobe (va) is more or less chitinized and is smaller than or equal to vb-II; and the right lobe (rl) is membranous, typically weak, scaled or not, and is on the right side of the endophallus and close to the gp. Additional lobes are present only in *Pterostichus adelphus* sp. n. (Fig. 34), with a pre-apical lobe (pa) that may derive from the rl and a ventral-basal lobe III (vb-III) that may derive from the vb-II.

**Characters**. **Definitive characters**. Pronotum with two or more mid-lateral setae; endophallus as type I. **Exclusive characters**. Male penultimate sternum modified; median lobe of aedeagus relatively stout, bent less than 90 degrees; apical lamella of aedeagus with apex hooked to left; inner margin of stylomere II with two ensiform setae near middle. **Common characters.** Pronotal hind angle completely rounded, posterior seta distant from hind angle; apical lamella of aedeagus with length more than half its basal width, gradually narrowed to apex; apical orifice of aedeagus not opened on ventral side; receptaculum capitate.

**Monophyly**. The monophyly of the *agilis*-group appears clearly and is suggested by two characters (supposedly apomorphic), the numerous pronotal mid-lateral setae and the endophallic characters (see details above). Moreover, the isolated distribution of the *agilis*-group is also consistent with its monophyly.

**Affinities.** *Pterostichus pohnerti* could be the species with the most affinity to the *agilis*-group because its endophallus is most similar to the *agilis*-group (Fig. 47). The endophallus of *Pterostichus pohnerti* is also slightly bent to the ventral side of the aedeagus across its apical lamella. However, unlike the *agilis*-group, *Pterostichus pohnerti* has the major portion of its endophallus located beyond the apical lamella, and therefore, the endophallus is not completely bent to the venter (also, see the above discussion on endophallus types). The affinity between *Pterostichus pohnerti* and the *agilis*-group is also suggested by their geographical distributions; the range of *Pterostichus pohnerti* is very close to the range of the *agilis*-group (Map 2).

**Interspecific relationships**. *Pterostichus adelphus* sp. n. and *Pterostichus cavazzutianus* appear to be close to one another based on their similarities in linear elytral microsculpture and unmodified male penultimate sternum. However, the unique endophallus of *Pterostichus adelphus* may suggest otherwise; the endophallus has two additional lobes (vb-III, pa), which makes the endophallus more complex than any other species in the *agilis*-group.

The other four species (*Pterostichus agilis*, *Pterostichus zhygealu* sp. n., *Pterostichus camelus* sp. n., and *Pterostichus bullatus*) are considered close because of their similarities in elytral microsculpture (transverse or isodiametric) and apical lamella of the aedeagus (longer than one-third of the apical orifice length). *Pterostichus bullatus* could be relatively isolated from the other three because of its unmodified male penultimate sternum and different endophallus (vb-II is much longer than the va in *Pterostichus bullatus*, and vb-II is approximately the same length as va in *Pterostichus agilis* and *Pterostichus zhygealu* sp. n.). A close relationship between *Pterostichus zhygealu* sp. n. and *Pterostichus agilis* is suggested by their similar endophalli and by the similar apical lamella and male penultimate sternum.

The peculiar species *Pterostichus camelus* sp. n. has some special characters in the subgenus: the fifth tarsomeres setose; the male penultimate sternum with two large tubercles; and the female sternum VIII with small transparent regions isolated from the major one. Although we did not study the endophallus of *Pterostichus camelus* sp. n., a close relationship between *Pterostichus camelus* sp. n. and *Pterostichus agilis* is suggested by their modified penultimate sterna and relatively slender body forms.

### 2) *baenningeri*-group

**Geographical distribution.** This species group contains nine species, including two previously described species and seven new species described in the present paper. The range of the *baenningeri*-group is wider than the sum of all other species group distributions, but it does not overlap with any other species group (Map 1). Species of the *baenningeri*-group have been recorded in five provinces: Yunnan, Guangxi, Guizhou, Chongqing, and Hubei. Yunnan has the richest fauna with four species. In the *baenningeri*-group, most species are strictly allopatric, except for the two sympatric species (*Pterostichus maitreya* sp. n. and *Pterostichus tumulus* sp. n.) in the Fanjingshan Mountains (Guizhou). Most species of this group have been recorded from a single locality, except *Pterostichus liciniformis*, which has a relatively wide distribution in northwestern Yunnan.

**Endophallus**. In all species of the *baenningeri*-group, the apical orifice opens on the ventral side of the median lobe, the endophallus is bent to the ventral side of the aedeagus from the ventral opening, the major portion of the endophallus is located on the ventral side of the aedeagus, and the gonopore orientation is variable (endophallus type II). The basal plan of the endophallus in the *agilis*-group has five lobes (Figs 40–44): the basal lobe (bl) is small and close to the ventral side of the apical lamella base; the ventral lobe (vl) is large and highly variable (simple, chitinized, or divided into two parts); the right lobe (rl) is small and membranous with scales; the left lobe (lf) is large and variable and has one or two parts; and the pre-apical lobe (pa), which is typically indistinct, weakly pointed or absent. The endophallus in the *baenningeri*-group is more variable than in the *agilis*-group, and the variability is primarily represented in the trend of the endophallus main body and gonopore orientation. However, concerning the homology of the endophallic lobes, the endophalli of the five species studied in the present study are generally consistent with the basal plan, with the exception of *Pterostichus dimorphus* sp. n. with the bl absent; *Pterostichus wangjiani* sp. n. with the pa chitinized on the apex; and *Pterostichus maitreya* sp. n. with one additional small lobe (dl).

**Characters**. **Definitive characters**. Apical orifice opened on ventral side of median lobe; endophallus of type II. **Exclusive characters**. Pronotal hind angle forming obtuse angle; posterior seta close to hind angle, distance between seta and hind angle much shorter than the distance between hind angle and inner basal foveal groove; elytral shoulder angle completely rounded, without humeral tooth; female elytral microsculpture granular; receptaculum clavate. **Common characters**. Pronotum with one mid-lateral seta; male terminal sternum modified; male penultimate sternum not modified; apical lamella of aedeagus with length equal to or greater than basal width.

**Monophyly**. The monophyly of *baenningeri*-group appears clear, as suggested by the consistent and exclusive endophallus type (for details, see above) and by the distinctly modified male terminal sternum. Outside of the *baenningeri*-group, only one species (*Pterostichus cavazzutianus* in the *agilis*-group) has the male terminal sternum modified, but less distinctly than all members of the *baenningeri*-group. Moreover, the isolated distribution of the *baenningeri*-group is also consistent with its monophyly.

**Affinities.** It is difficult to estimate the affinities of the *baenningeri*-group because the male genitalia in this species group are different from all other species groups and no intermediate forms are found. However, the modified male terminal sternum might indicate a relationship to *Pterostichus cavazzutianus*.

**Interspecific relationships**. From the shape of the receptaculum and the male terminal sternum character, the nine species in the *baenningeri*-group are divided into two subgroups.

Subgroup I contains five species (*Pterostichus baenningeri*, *Pterostichus maitreya*, *Pterostichus yan*, *Pterostichus miao*, and *Pterostichus ailaoicus*) that have the following characters: (1) male terminal sternum with two tubercles; (2) female spermathecal receptaculum capitate; (3) median lobe of the aedeagus slenderer than other species; and (4) elytral microsculpture linear. Although it is difficult to estimate the evolutionary polarity of each character before a comprehensive cladistic study, we suggest a monophyletic clade for this subgroup based on their accordant male sterna and aedeagi. In subgroup I, the interspecific relationships are difficult to estimate.

Subgroup II contains four species (*Pterostichus liciniformis*, *Pterostichus dimorphus*, *Pterostichus wangjiani*, and *Pterostichus tumulus*) that have the following characters: (1) male terminal sternum concave or with one elongate tubercle; (2) female spermathecal receptaculum clavate; (3) median lobe of the aedeagus relatively stout; and (4) elytral microsculpture variable. The clavate receptaculum suggests the monophyly of subgroup II because all other species in *Circinatus* have capitate receptaculum. The three species from northern Yunnan appear to be allied because of their similarity in the male terminal sternum (concave in the middle). However, the median lobe of the aedeagus in *Pterostichus tumulus* sp. n. is very similar to some species in subgroup I (Fig. 32), and therefore, subgroup II may not be a monophyletic group. Under this supposition, the capitate receptaculum and the concave male terminal sternum should be considered plesiomorphic states (comparisons with allied subgenera also suggest these states), and subgroup II may contain early branches of the *baenningeri*-group.

### 3) *subtilissimus*-group

**Geographical distribution.** This species group contains three species, including two previously described species and one new species described in the present paper. The three species in the *subtilissimus*-group are strictly allopatric from one another and are known from only a single locality for each. However, each of these three species is sympatric with one or two species from the *pohnerti*-group. Moreover, the species of the *subtilissimus*-group are significantly rarer and larger in body size than their sympatric species from the *pohnerti*-group. All species of the *subtilissimus*-group and the *pohnerti*-group are distributed in central Sichuan Province. However, the range of the *subtilissimus*-group is narrower than that of the *pohnerti*-group (Map 2).

**Endophallus**. We studied the endophallus of two species (*Pterostichus subtilissimus* and *Pterostichus yuxiaodongi* sp. n.) in the *subtilissimus*-group. The endophallus is bent to the dorsal side of the aedeagus, the major portion is located on the dorsal side of the aedeagus (endophallus type III), and the gonopore points to the aedeagal base or apex. The endophallus decoration is simpler than in the other three species groups: without chitinized pieces, with at least two lobes (ml and bl in Figs 45, 46) present, and with lobes glabrous or with very fine scales.

**Characters**. **Definitive characters**. Median lobe of aedeagus strongly bent to right side at approximately apical third; body size largest in the subgenus, 14.4–16.5 mm in length (in the other species groups, the largest record is a 14.5 mm specimen of *Pterostichus zhygealu* sp. n.). **Exclusive characters**. Elytral basal ridge strongly oblique lateral-posteriorly. **Common characters**. Pronotum with one mid-lateral seta; elytral microsculpture transverse or isodiametric; male terminal and penultimate sterna not modified; endophallus type III, without chitinized pieces.

**Monophyly**. The three species in the *subtilissimus*-group are closely allied and their monophyly appears clear, suggested by their relatively large body size and the median lobe apex strongly bent to the right side. Moreover, based on the proposition of a close relationship between the *subtilissimus*-group and *Pterostichus beneshi* + *Pterostichus zoiai* (vide infra), the monophyly of the *subtilissimus*-group is also inferred from the following characters: elytral microsculpture transverse or isodiametric, pronotal outer basal foveal groove present, and area between inner and outer basal foveal grooves depressed.

**Affinities.** From the similarities of the endophalli, the *subtilissimus*-group could be closest to *Pterostichus beneshi* and *Pterostichus zoiai* in the *pohnerti*-group. These species are similar because the endophallus is bent to the dorsal side of the aedeagus, without chitinized piece.

**Interspecific relationships**. Because of the similar aedeagi (median lobe of the aedeagus relatively slender, not constricted near the apex), the strongly oblique elytral basal ridges, and the close distributions, *Pterostichus dentifer* and *Pterostichus yuxiaodongi* sp. n. could be close to one another.

### 4) *pohnerti*-group

**Geographical distribution.** This species group contains four species, which are all previously described and are all distributed in central Sichuan Province. The range of the *pohnerti*-group, although wider, overlaps the distribution of the *subtilissimus*-group (Map 2). Except for *Pterostichus zoiai*, which is known from only a single locality, the other three species are more widely distributed. *Pterostichus pohnerti* and *Pterostichus xilingensis* are strictly allopatric, as are *Pterostichus beneshi* and *Pterostichus zoiai*. The range of *Pterostichus beneshi* overlaps those of *Pterostichus pohnerti* and *Pterostichus xilingensis* (Map 3).

**Endophallus**. The endophallus is highly variable in this species group. The endophallus in *Pterostichus beneshi* and *Pterostichus zoiai* is located on the dorsal side of the aedeagus and is strongly bent to the aedeagal base (endophallus type III). The endophallus has scales, without chitinized pieces, and the gonopore points to the dorsal side of the aedeagus. In *Pterostichus pohnerti*, the endophallus is weakly bent to the ventral side across the apical lamella, the major portion of the endophallus is located beyond the apical lamella, and the endophallus has scales and chitinized pieces. The gonopore points to the aedeagal base. In *Pterostichus xilingensis*, the endophallus is located on the dorsal side of the aedeagus and extends to the right side of the aedeagus and has scales and chitinized pieces. The gonopore points to the right side of the aedeagus.

**Characters**. **Common characters**. Pronotum with one mid-lateral seta; hind angle completely rounded; posterior seta distant from hind angle; elytron with iridescent shine; elytral microsculpture linear; male terminal and penultimate sterna not modified.

**Monophyly**. The four species in the *pohnerti*-group have similar external characters and adjacent ranges, but their male genitalia are very different from one another, primarily in the endophallus (location and decoration, see above). Based on the diversity of the endophallus, we suppose that these four species form at least a paraphyletic group and may represent relatively basal clades in the subgenus. We propose this species group not for its monophyly but for its morphological accordance and similarity, and not for the purpose of systematics but for convenient taxa cognition in classification. Neither dividing the present *pohnerti*-group into two or three species groups nor combining all or some of the species to other species groups will provide a better infra-subgeneric system.

**Affinities.** For the endophallus characters, we propose the following affinities: *Pterostichus pohnerti* has relative affinity to the *agilis*-group; *Pterostichus beneshi* + *Pterostichus zoiai* have affinity to the *subtilissimus*-group; and the position of *Pterostichus xilingensis* is relatively isolated or closer to *Pterostichus pohnerti*.

**Interspecific relationships**. *Pterostichus beneshi* and *Pterostichus zoiai* are surely closely allied with one another in their accordant external and genitalia characters; they are two allopatric sister species. However, the relationship between *Pterostichus pohnerti* and *Pterostichus xilingensis* is ambiguous.

### Phylogenetic analysis of *Circinatus*

A phylogenetic analysis was conducted to provide more support for the above taxonomic distinctions. A total of 36 characters was selected, which included ten external characters, eleven male genital characters (including male secondary sexual characters on the sterna), nine male endophallus characters, and six female genital characters. Two of the characters were multistate, whereas the others were binary. Although all characters were unordered in the phylogenetic analysis (without demonstration of character polarities), the supposed plesiomorphic states were coded “0”. All 23 of the species and subspecies in *Circinatus* were included in the phylogenetic analysis, but some of them lacked female genitalia or male endophallus characters. Two species of the *Pterostichus* subgenera *Gutta* and *Sinosteropus* were selected as out-groups. The information on the matrix, character coding, and examined material of the out-groups is found in the Appendix.

### Phylogeny reconstruction

The phylogeny reconstruction was performed using WIN-PAUP* Version 4.0b10 with the following parameters: optimality criterion = parsimony; all characters were unordered; starting tree(s) was obtained via stepwise addition; addition sequence: random; number of replicates = 1000; number of trees held at each step during stepwise addition = 10; branch-swapping algorithm: TBR; steepest descent option not in effect; initial ‘MaxTrees’ setting = 100; ‘MulTrees’ option in effect; topological constraints not enforced; trees unrooted; bootstrap method with heuristic search; and number of bootstrap replicates = 1000. Both the equal weighting method (EW) and the successive weighting method (SW) were used in the phylogeny reconstruction. Branches with bootstrap values greater than 50% were maintained.

**Figure 1.**
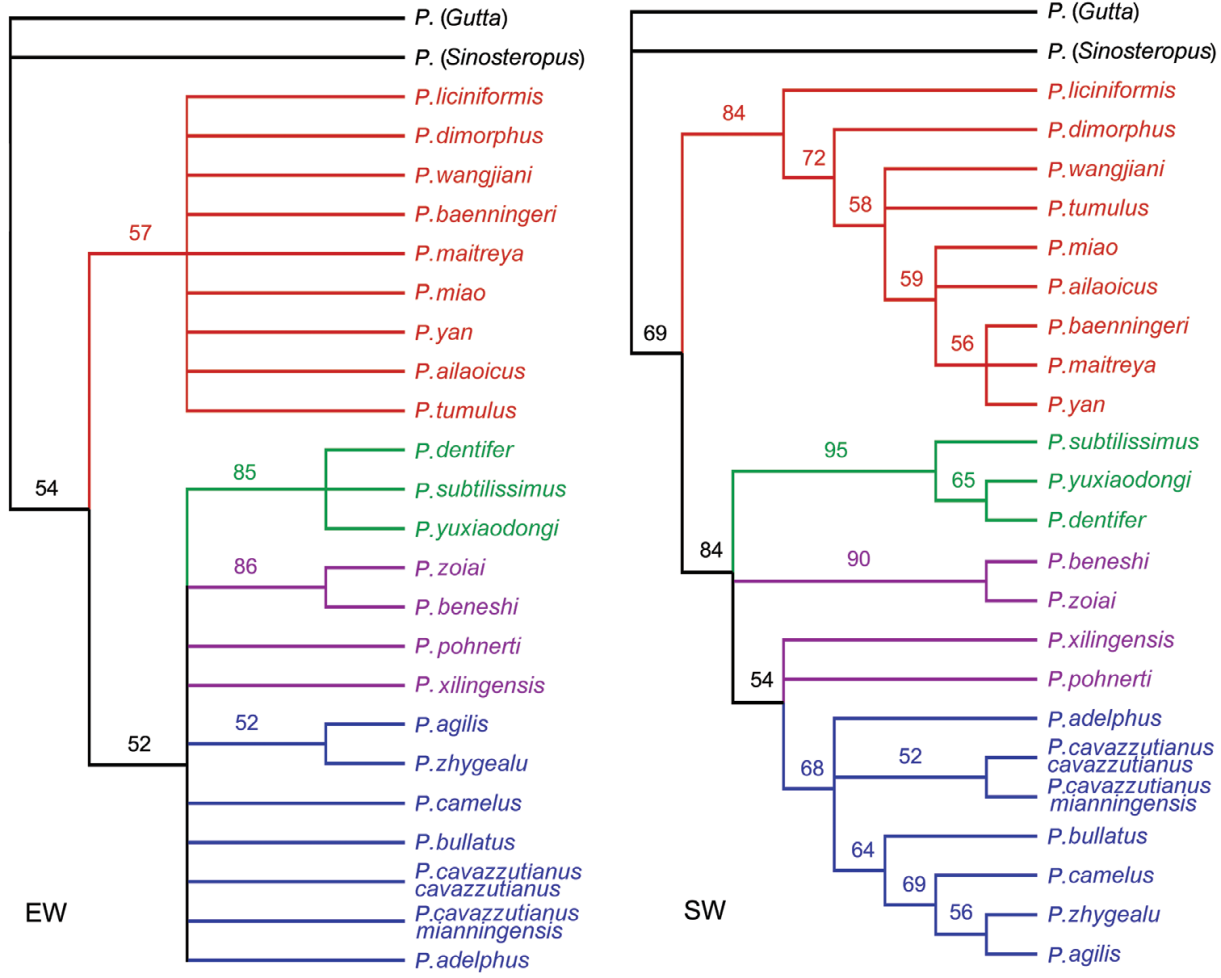
Cladograms of *Circinatus* species relationships with bootstrap values greater than 50%. EW: cladogram obtained by the equal weighting method; SW: cladogram obtained by the successive weighting method. Different colors show the four species groups: red, *baenningeri*- group; green, *subtilissimus*-group; purple, *pohnerti*-group; and blue, *agilis*-group.

The two cladograms from our heuristic analyses are shown in Fig. 1. In the EW analysis, the two most parsimonious trees were obtained with the same tree length and indices: tree length = 100; consistency index (CI) = 0.380; homoplasy index (HI) = 0.620; retention index (RI) = 0.675; and rescaled consistency index (RC) = 0.257. In the SW analysis, the two most parsimonious trees were obtained. For Tree I, the length and indices were as follow: tree length = 25.27; consistency index (CI) = 0.570; homoplasy index (HI) = 0.430; retention index (RI) = 0.846; and rescaled consistency index (RC) = 0.482. For Tree II, the length and indices were as follow: tree length = 24.39; consistency index (CI) = 0.591; homoplasy index (HI) = 0.409; retention index (RI) = 0.859; and rescaled consistency index (RC) = 0.507. The two most parsimonious trees were obtained for each of the EW and the SW analyses, and the topological configurations of the strict consensus of the two trees from the EW and SW methods were the same.

The cladograms generated with the EW and SW methods had no topological conflicts but did have different reliabilities. The SW tree had higher bootstrap values than the EW tree on most branches. Two monophyletic lineages were supported with relatively high reliabilities (bootstrap values >50% in EW and >80% in SW) in both the EW and SW analyses: Lineage I = *baenningeri*-group; Lineage II = *subtilissimus*-group + *pohnerti*-group + *agilis*-group. However, in the EW analysis, most species relationships in both lineages were unresolved, except for the monophyly of the *subtilissimus*-group, which was supported by a relatively high bootstrap value (= 85).

In the SW analysis, most species relationships were resolved with relatively high reliabilities. Three of the four species groups were suggested as monophyletic, with the exception of the *pohnerti*-group. In the *baenningeri*-group, a monophyletic clade composed of five species was supported (*Pterostichus miao* + *Pterostichus ailaoicus* + *Pterostichus baenningeri* + *Pterostichus yan* + *Pterostichus maitreya*, namely, subgroup I of the *baenningeri*-group, see above). The other four species formed early branches with the earliest branch of *Pterostichus liciniformis*. The monophyly of the *subtilissimus*-group was well supported (= 95), with a close relationship between *Pterostichus yuxiaodongi* and *Pterostichus dentifer*. In the *agilis*-group, a monophyletic clade composed of four species was supported, but the relationships of the other two species were unresolved. In the *pohnerti*-group, the only paraphyletic species group, the relationships among these four species and those among the four species and the other monophyletic clades remained unresolved. As indicated in the previous section, the proposal for the *pohnerti*-group was only an expedient arrangement for these four species. In the SW analysis, neither the clade of *Pterostichus pohnerti* + *Pterostichus xilingensis* + *agilis*-group nor that of *Pterostichus zoiai* + *Pterostichus beneshi* + *subtilissimus*-group was supported by relatively high bootstrap values.

In the present paper, the species relationships of *Circinatus* were discussed based on evolutionary systematics and phylogenetic systematics methods in separate sections. The major results (e.g., monophyly of the main clades) are in accord for both systematics approaches. However, conflicts are also present, such as some of the species relationships in each species group. We noticed flaws in both systematics analyses in the present work, which did not tend to resolve the species relationships further.

### Character evolution

Character evolution was reconstructed using WinClada and NONA software on one of the most parsimonious trees obtained with the SW analysis (tree I). The ancestral states were reconstructed considering only unambiguous transformations. The unambiguous character evolutions are shown with circles on the branches, and the apomorphies, parallelisms and reversals are shown in separate marks.

**Figure 2.**
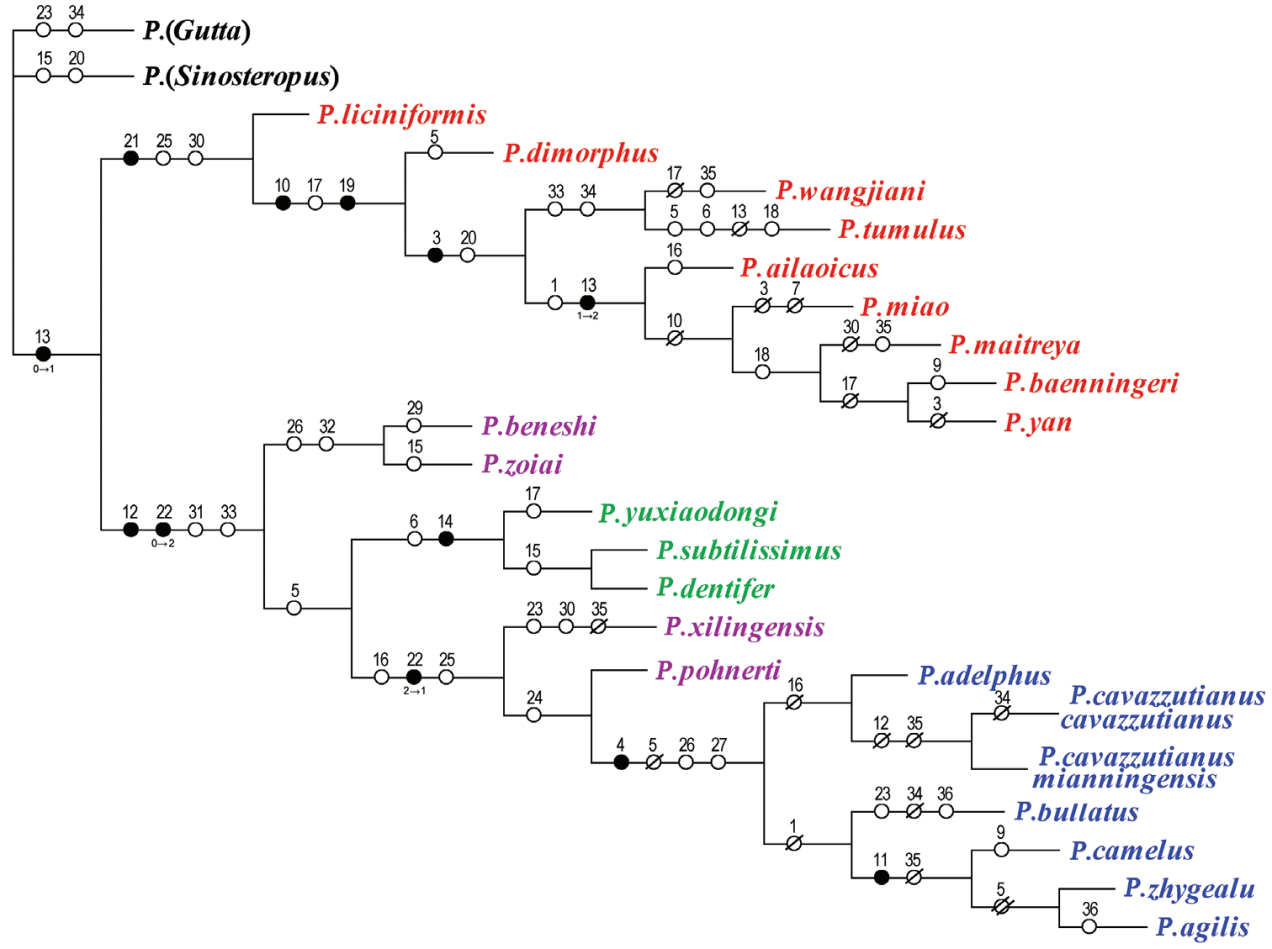
Possible character evolution of the subgenus *Circinatus* based on one of the most parsimonious trees (tree I) obtained from the SW analysis. Solid circles represent apomorphies; empty circles represent parallelisms and reversals; reversals are marked with a slash; twice-reversals are marked with a double slash; multistate evolution is marked under the characters. The species groups are shown in different colors, as in Figure 1.

The monophyly of the *subtilissimus*-group + *pohnerti*-group + *agilis*-group was supported by: male terminal sternum unmodified (char. 12: 1); major portion of endophallus located on the ventral or dorsal to the ventral-apical side of aedeagus (char. 22: 1, 2); spermatheca receptaculum capitate (char. 31: 1); and basal sclerotized piece of seminal canal distinct (char. 33: 1) (Fig. 2). The monophyly of the *baenningeri*-group was supported by: apical orifice opened on the ventral side (char. 21: 1); chitinized structure present on endophallus ventral side (char. 25: 1); gonopore oriented to aedeagus apex (char. 30: 1). The monophyly of the *subtilissimus*-group was supported by: pronotum base depressed between outer and inner basal foveal grooves (char. 6: 1); median lobe of aedeagus apex strongly bent to right (char. 14: 1). The monophyly of the *agilis*-group was supported by: pronotum with two or more mid-lateral setae (char. 4: 1); pronotal outer basal foveal groove absent (char. 5: 0, reversal); ventral lobe of endophallus divided into sub-lobes (char. 26: 1); endophallus with right lobe on the right side (char. 27: 1).

Nineteen unambiguous character transformations that belonged to 13 characters (slash in Fig. 2) had reversals. Only character 5 (pronotal outer basal foveal groove present or not) reversed twice. A total of 12 characters transformations were apomorphies, including two multistate characters (char. 13, 22).

Some characters were considered important in the above discussion (the section on infra-subgeneric taxonomy). The male elytral microsculpture (char. 1) transformed at least five times but only two of the transformations were unambiguous, which supported the two clades of the *baenningeri*-group and the *agilis*-group. The position of pronotal posterior seta (char. 2) transformed twice, both ambiguously, which might have transformed from 0 to 1 on the clade of (*Pterostichus wangjiani* + *Pterostichus tumulus* + *Pterostichus ailaoicus* + *Pterostichus miao* + *Pterostichus maitreya* + *Pterostichus baenningeri* + *Pterostichus yan*) and reversed to 0 on *Pterostichus tumulus*, under fast optimization. The pronotal mid-lateral setae number (char. 4) transformed only once, which supported the monophyly of the *agilis*-group. The modification of male terminal sternum (char. 12) unambiguously transformed twice. The first transformation (0→1) supported the monophyly of (*pohnerti*-group + *subtilissimus*-group + *agilis*-group) and was reversed in the species *Pterostichus cavazzutianus*. The apical orifice, whether opened on ventral side (char. 21), was transformed only once, which supported the monophyly of the *baenningeri*-group. The spermatheca receptaculum shape (char. 31) transformed approximately twice (some species lack this character), which supported the monophyly of (*pohnerti*-group + *subtilissimus*-group + *agilis*-group) and a clade in the *baenningeri*-group.

**Figure 3.**
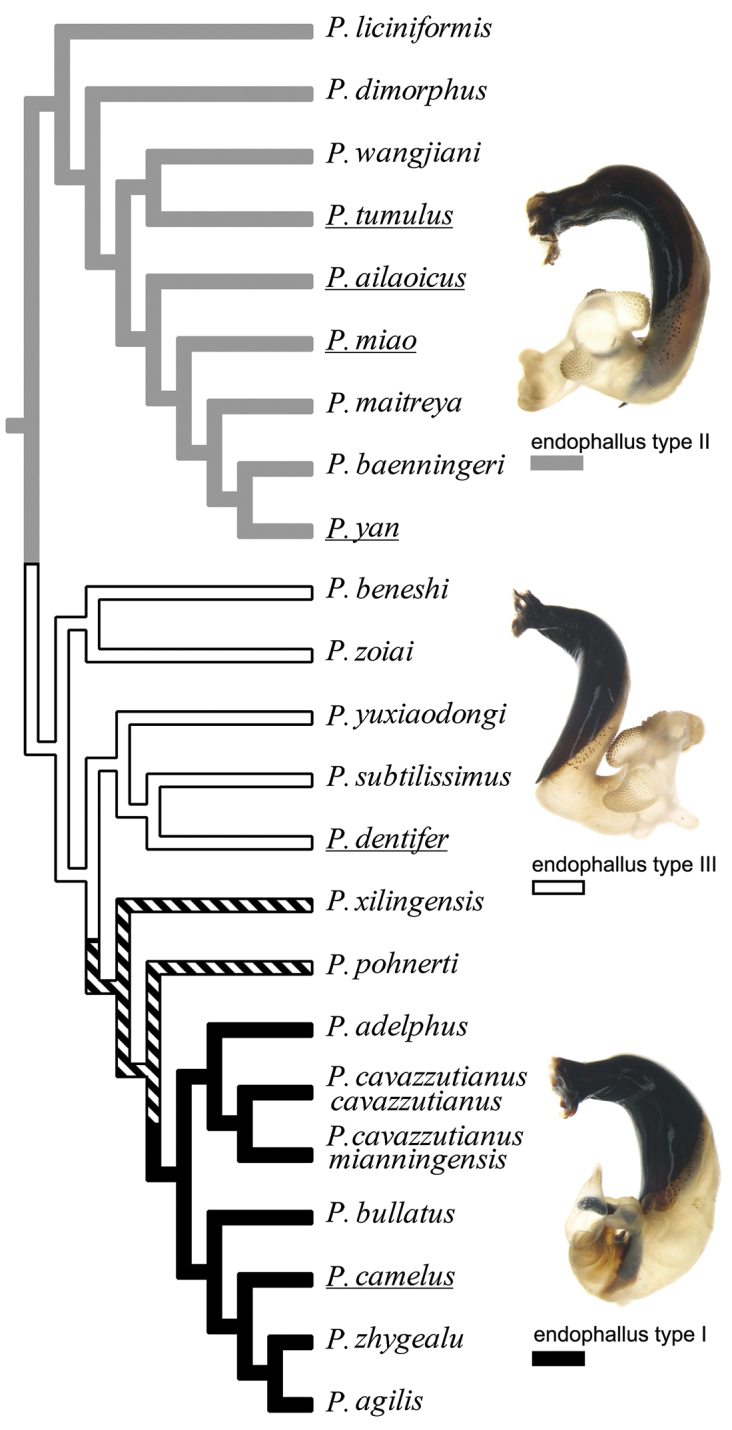
The evolution of the male endophallus in the subgenus *Circinatus* based on the most parsimonious tree obtained with the SW analysis. Black: endophallus type I; gray: endophallus type II; white: endophallus type III; black-white strips show the intermediate status between endophallus types I and III. The species with an unknown endophallus are marked with an underline.

The shape of the male endophallus was particularly emphasized in the character analysis. Approximately accordant with the three states of character 22 were the three types of endophallus (types I, II, and III), with small differences for two species (*Pterostichus pohnerti* and *Pterostichus xilingensis*). We redrew the evolution of endophallus types based on one of the most parsimonious trees (Fig. 3). The evolution polarity of the endophallus was type II → type III → type I. Type I and type II were only represented in one monophyletic clade of the subgenus. However, type I was apomorphic and supported the monophyly of the *agilis*-group, whereas type II was plesiomorphic. Type III was represented in a paraphyletic group. The two species (*Pterostichus pohnerti* and *Pterostichus xilingensis*) with intermediate status between type I and type III were in an intermediate systematic position between species with type I and type III endophalli.

## Plates

**Figures 4–9.**
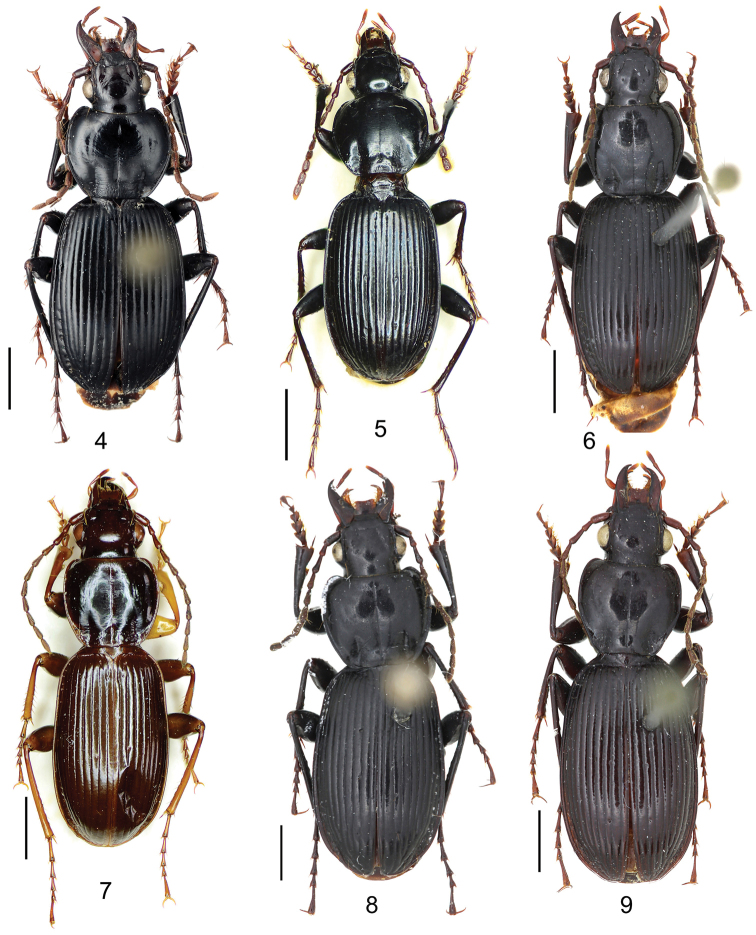
Habitus of *Pterostichus (Circinatus)* spp. **4** *Pterostichus adelphus* sp. n., holotype **5** *Pterostichus cavazzutianus* s. str. replacement name, paratype **6** *Pterostichus cavazzutianus mianningensis* n. ssp., holotype **7** *Pterostichus agilis* Allegro & Sciaky, holotype **8** *Pterostichus zhygealu* sp. n., holotype **9** *Pterostichus camelus* sp. n., holotype. Scale bar: 2.0 mm.

**Figures 10–15.**
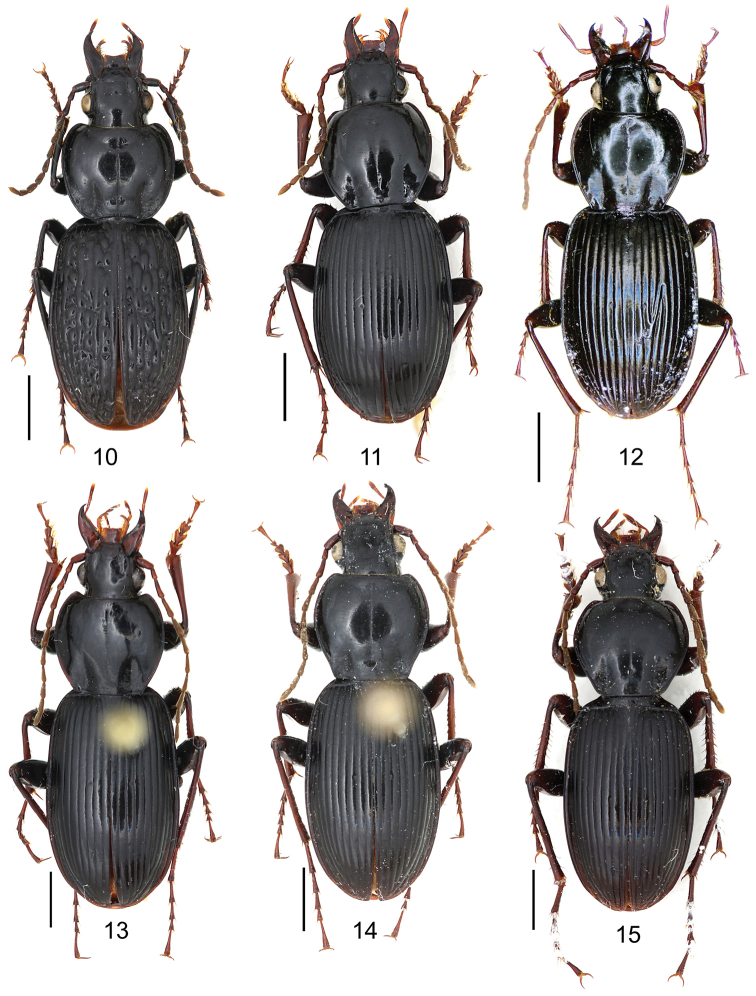
Habitus of *Pterostichus (Circinatus)* spp. **10** *Pterostichus bullatus* Allegro & Sciaky **11** *Pterostichus ailaoicus* sp. n., holotype **12** *Pterostichus baenningeri* Jedlička, lectotype **13** *Pterostichus maitreya* sp. n., holotype **14** *Pterostichus miao* sp. n., holotype **15** *Pterostichus yan* sp. n., holotype. Scale bar: 2.0 mm.

**Figures 16–21.**
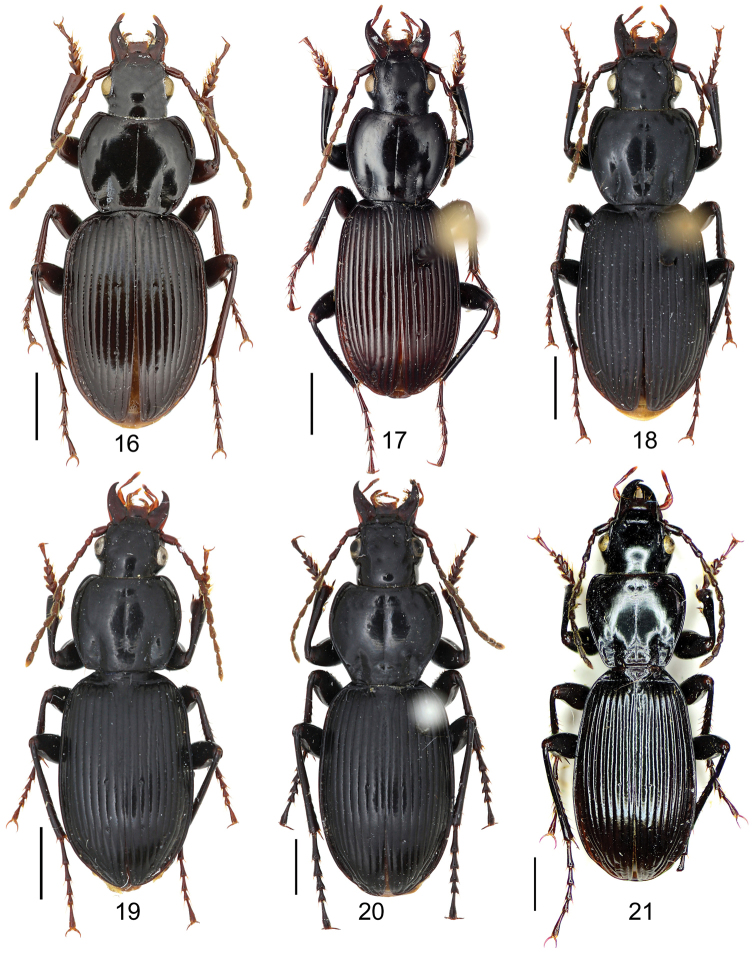
Habitus of *Pterostichus (Circinatus)* spp. **16** *Pterostichus liciniformis* Csiki, locality: Lijiang **17** *Pterostichus dimorphus* sp. n., holotype, male **18** *Pterostichus dimorphus* sp. n., paratype, female **19** *Pterostichus wangjiani* sp. n., holotype **20** *Pterostichus tumulus* sp. n., holotype **21** *Pterostichus subtilissimus* Sciaky, holotype. Scale bar: 2.0 mm.

**Figures 22–27.**
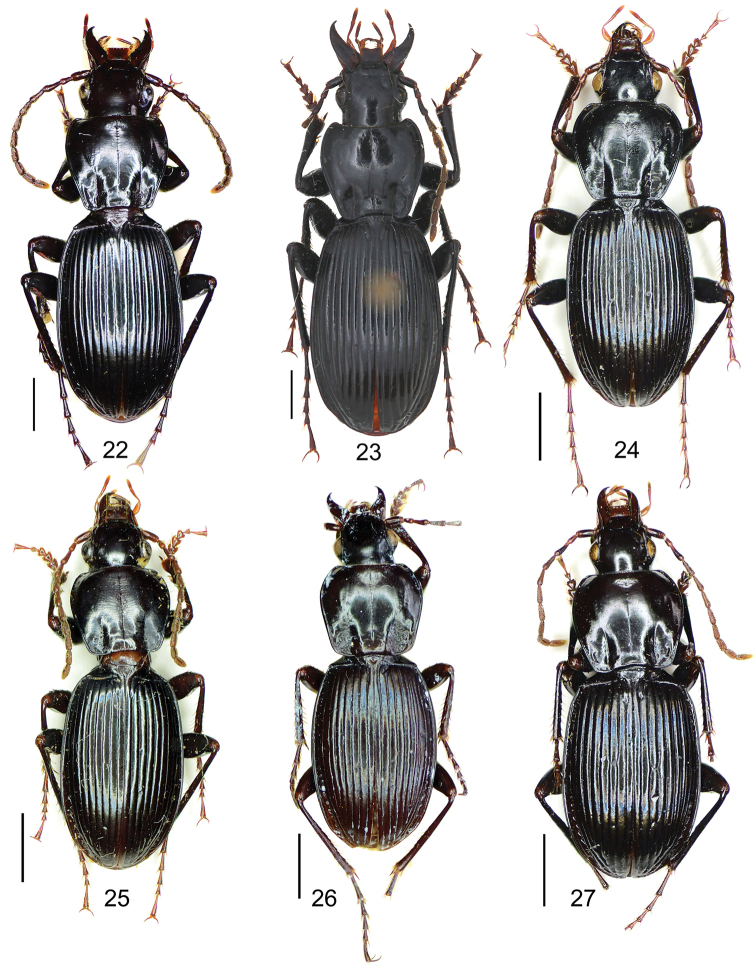
Habitus of *Pterostichus (Circinatus)* spp. **22** *Pterostichus dentifer* Allegro & Sciaky, holotype **23** *Pterostichus yuxiaodongi* sp. n., holotype **24** *Pterostichus beneshi* Sciaky, holotype **25** *Pterostichus zoiai* Sciaky, holotype **26** *Pterostichus pohnerti* Jedlička, holotype **27** *Pterostichus xilingensis* Allegro & Sciaky, holotype. Scale bar: 2.0 mm.

**Figures 28–33.**
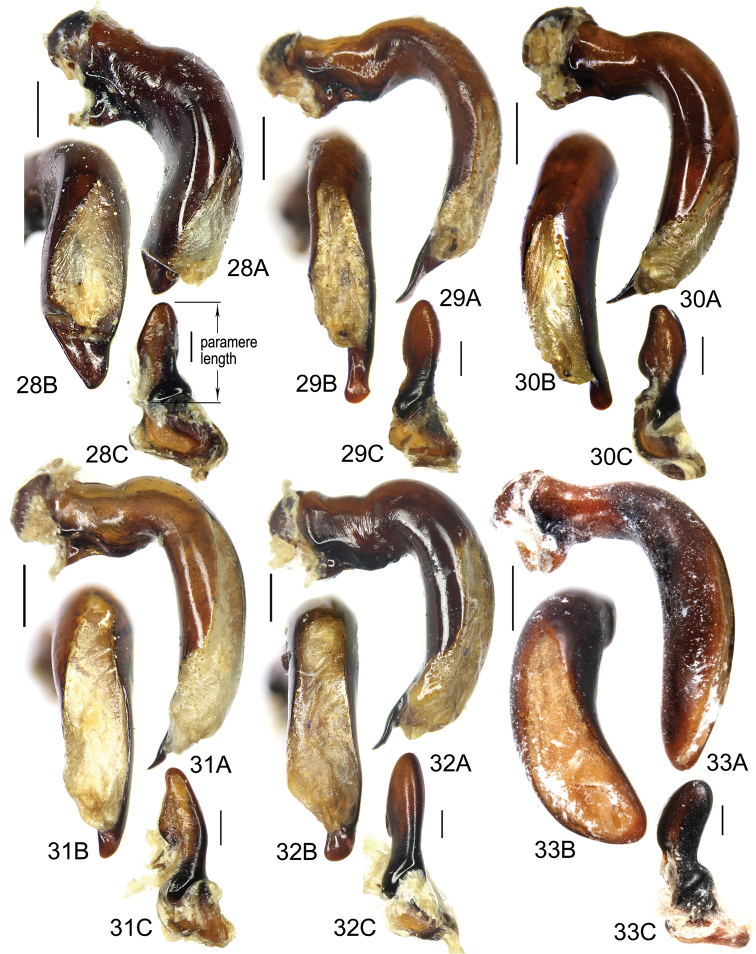
Male genitalia of *Pterostichus (Circinatus)* spp.; **A** left lateral view of median lobe **B** dorsal view of median lobe **C** right paramere **28** *Pterostichus camelus* sp. n., holotype **29** *Pterostichus ailaoicus* sp. n., holotype **30** *Pterostichus miao* sp. n. holotype **31** *Pterostichus yan* sp. n., holotype **32** *Pterostichus tumulus* sp. n., holotype **33** *Pterostichus dentifer* Allegro & Sciaky, holotype. Scale bars: 0.5 mm (**A, B**); 0.2 mm (**C**).

**Figures 34–36.**
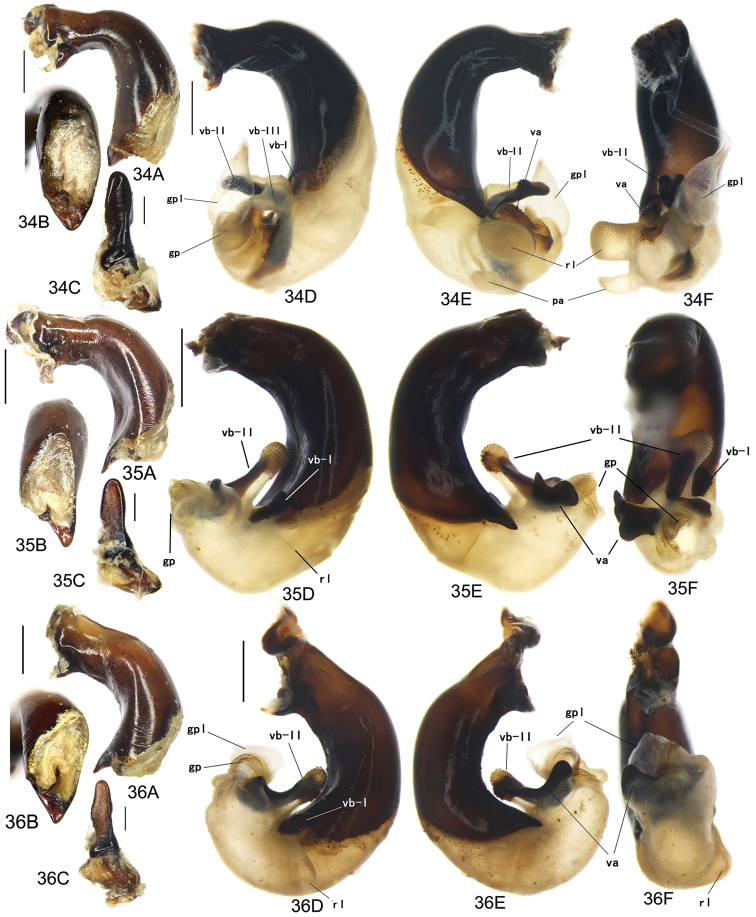
Male genitalia of *Pterostichus (Circinatus)* spp.; **A** left lateral view of median lobe **B** dorsal view of median lobe **C** right paramere **D** left lateral view of endophallus **E** right lateral view of endophallus **F** ventral view of endophallus **34** *Pterostichus adelphus* sp. n., holotype (**A, B, C**), paratype (**D, E, F**) **35** *Pterostichus cavazzutianus* s. str. replacement name, locality: Yizi pass **36** *Pterostichus cavazzutianus mianningensis* ssp. n., holotype (**A, B, C**), paratype (**D, E, F**). Abbreviations of endophallic lobes explained in description of each species. Scale bars: 0.5 mm (**A, B, D, E, F**); 0.2 mm (**C**).

**Figures 37–39.**
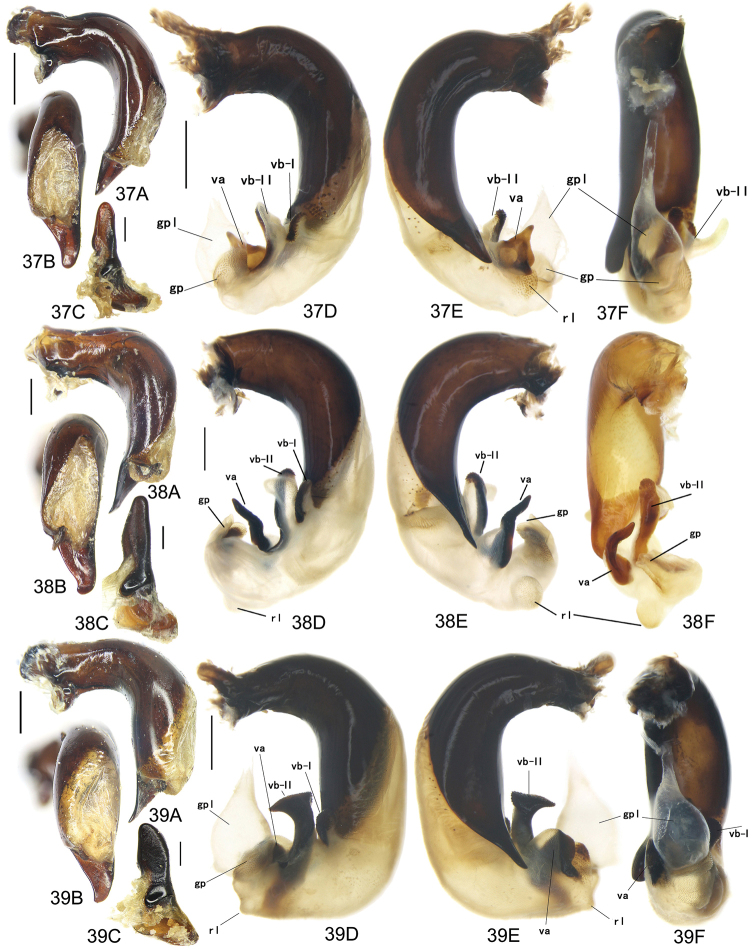
Male genitalia of *Pterostichus (Circinatus)* spp.; **A** left lateral view of median lobe **B** dorsal view of median lobe **C** right paramere **D** left lateral view of endophallus **E** right lateral view of endophallus **F** ventral view of endophallus **37** *Pterostichus agilis* Allegro & Sciaky, locality: Luojishan **38** *Pterostichus zhygealu* sp. n., holotype (**A, B, C**), paratype (**D, E, F**) **39** *Pterostichus bullatus* Allegro & Sciaky, locality: Yizi pass. Abbreviations of endophallic lobes explained in description of each species.Scale bars: 0.5 mm (**A, B, D, E, F**); 0.2 mm (**C**).

**Figures 40–42.**
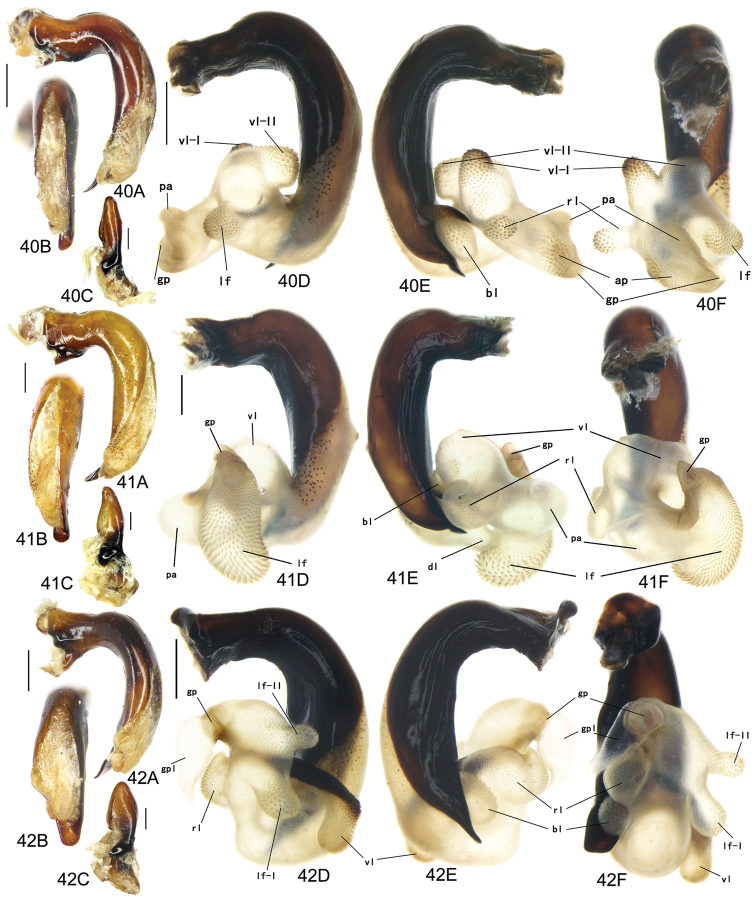
Male genitalia of *Pterostichus (Circinatus)* spp.; **A** left lateral view of median lobe **B** dorsal view of median lobe **C** right paramere **D** left lateral view of endophallus **E** right lateral view of endophallus **F** ventral view of endophallus **40** *Pterostichus baenningeri* Jedlička, locality: Jinfoshan **41** *Pterostichus maitreya* sp. n., holotype (**A, B, C**), paratype (**D, E, F**) **42** *Pterostichus liciniformis* Csiki, locality: Lijiang. Abbreviations of endophallic lobes explained in description of each species. Scale bars: 0.5 mm (**A, B, D, E, F**); 0.2 mm (**C**).

**Figures 43–45.**
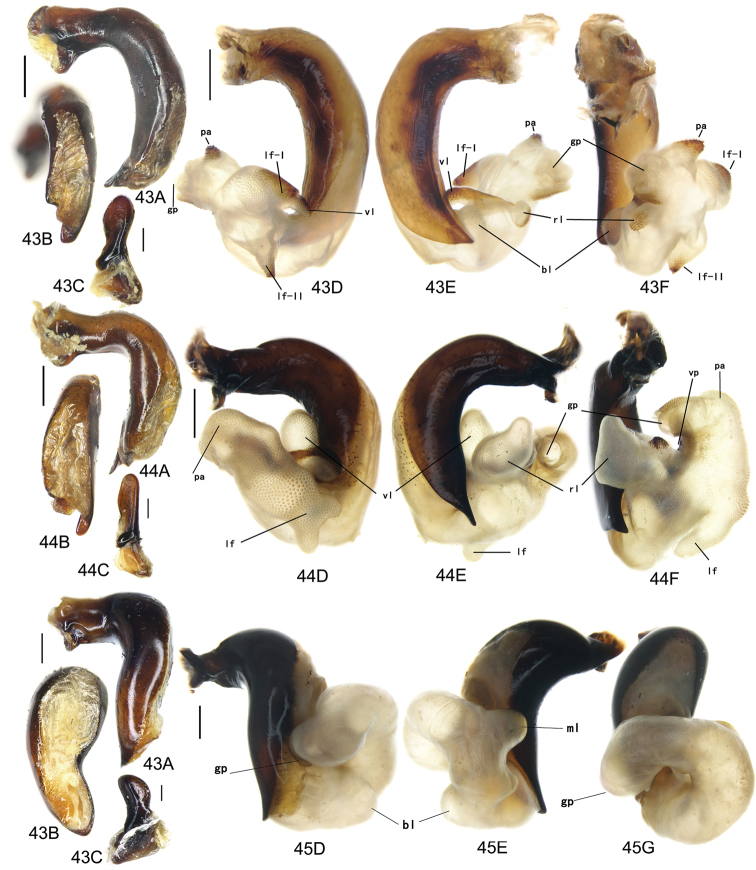
Male genitalia of *Pterostichus (Circinatus)* spp.; **A** left lateral view of median lobe **B** dorsal view of median lobe **C** right paramere **D** left lateral view of endophallus **E** right lateral view of endophallus **F** ventral view of endophallus **G** dorsal view of endophallus **43** *Pterostichus wangjiani* sp. n., holotype (**A, B, C**), paratype (**D, E, F**) **44** *Pterostichus dimorphus* sp. n., holotype (**A, B, C**), paratype (**D, E, F**) **45** *Pterostichus subtilissimus* Sciaky, locality: Emei mountain. Abbreviations of endophallic lobes explained in description of each species. Scale bars: 0.5 mm (**A, B, D, E, F, G**); 0.2 mm (**C**).

**Figures 46–48.**
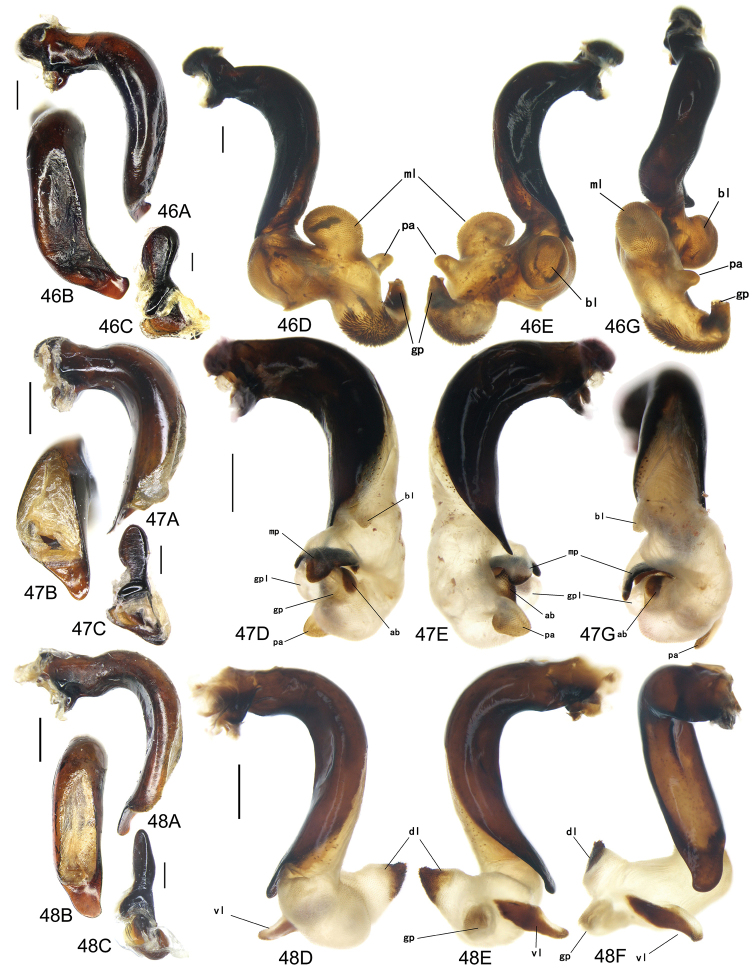
Male genitalia of *Pterostichus (Circinatus)* spp.; **A** left lateral view of median lobe **B** dorsal view of median lobe **C** right paramere **D** left lateral view of endophallus **E** right lateral view of endophallus **F** ventral view of endophallus **G** dorsal view of endophallus **46** *Pterostichus yuxiaodongi* sp. n., holotype **47** *Pterostichus pohnerti* Jedlička, locality: Hailuogou **48** *Pterostichus xilingensis* Allegro & Sciaky, locality: Wolong. Abbreviations of endophallic lobes explained in description of each species. Scale bars: 0.5 mm (**A, B, D, E, F, G**); 0.2 mm (**C**).

**Figures 49–52.**
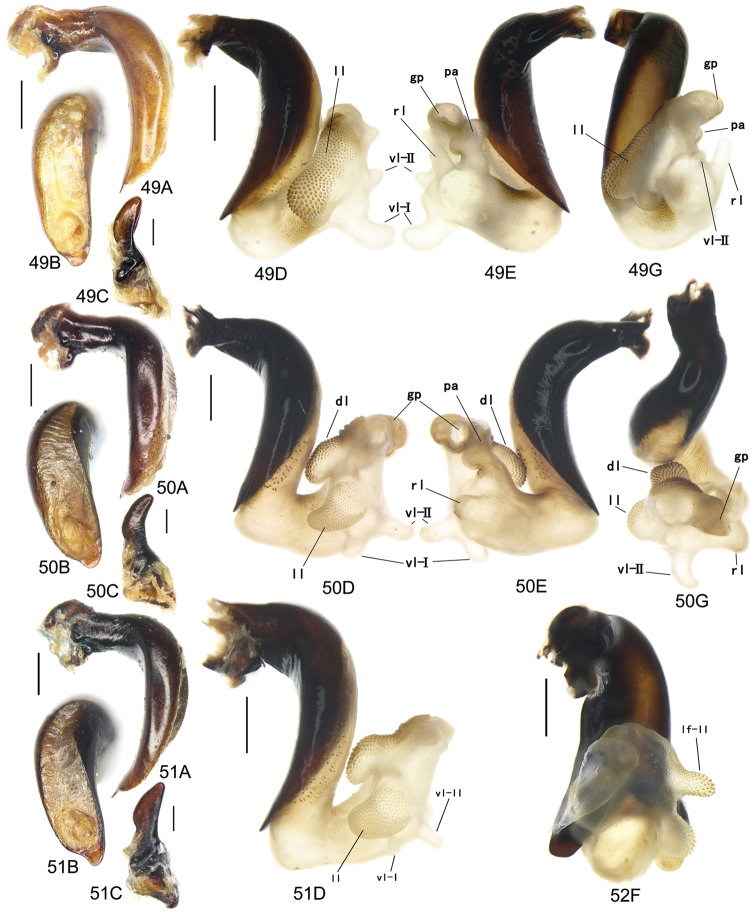
Male genitalia of *Pterostichus (Circinatus)* spp.; **A** left lateral view of median lobe **B** dorsal view of median lobe **C** right paramere **D** left lateral view of endophallus **E** right lateral view of endophallus **F** ventral view of endophallus **G** dorsal view of endophallus **49** *Pterostichus zoiai* Sciaky, locality: Emei mountain **50** *Pterostichus beneshi* Sciaky, locality: Erlangshan **51** *Pterostichus beneshi* Sciaky, locality: Wolong **52** *Pterostichus liciniformis* Csiki, locality: Zhongdian. Abbreviations of endophallic lobes explained in description of each species. Scale bars: 0.5 mm (**A, B, D, E, F, G**); 0.2 mm (**C**).

**Figures 53–70.**
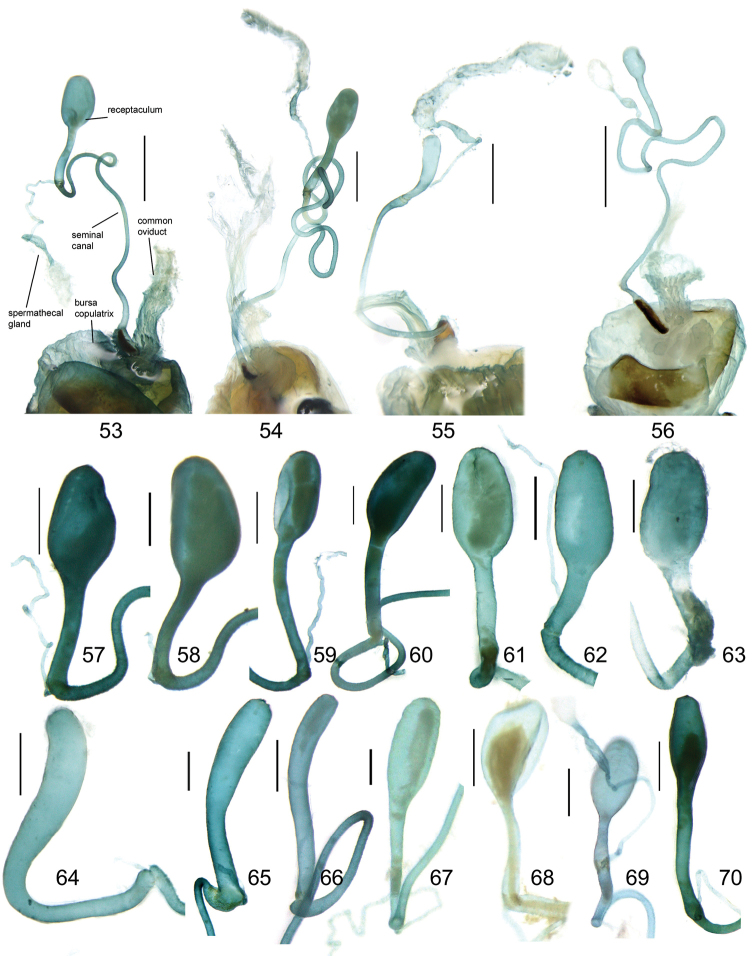
Female reproductive system of *Pterostichus (Circinatus)* spp. **57–70** show receptaculum only **53** *Pterostichus adelphus* sp. n. **54** *Pterostichus zhygealu* sp. n. **55** *Pterostichus tumulus* sp. n. **56** *Pterostichus pohnerti* Jedlička **57** *Pterostichus cavazzutianus* s. str. replacement name **58** *Pterostichus cavazzutianus mianningensis* ssp. n. **59** *Pterostichus agilis* Allegro & Sciaky **60** *Pterostichus camelus* sp. n. **61** *Pterostichus bullatus* Allegro & Sciaky **62** *Pterostichus baenningeri* Jedlička **63** *Pterostichus maitreya* sp. n. **64** *Pterostichus liciniformis* Csiki **65** *Pterostichus dimorphus* sp. n. **66** *Pterostichus wangjiani* sp. n. **67** *Pterostichus subtilissimus* Sciaky **68** *Pterostichus beneshi* Sciaky **69** *Pterostichus zoiai* Sciaky **70** *Pterostichus xilingensis* Allegro & Sciaky. Scale bars: 0.5 mm (**53–56**); 0.2 mm (**57–70**).

**Figures 71–89.**
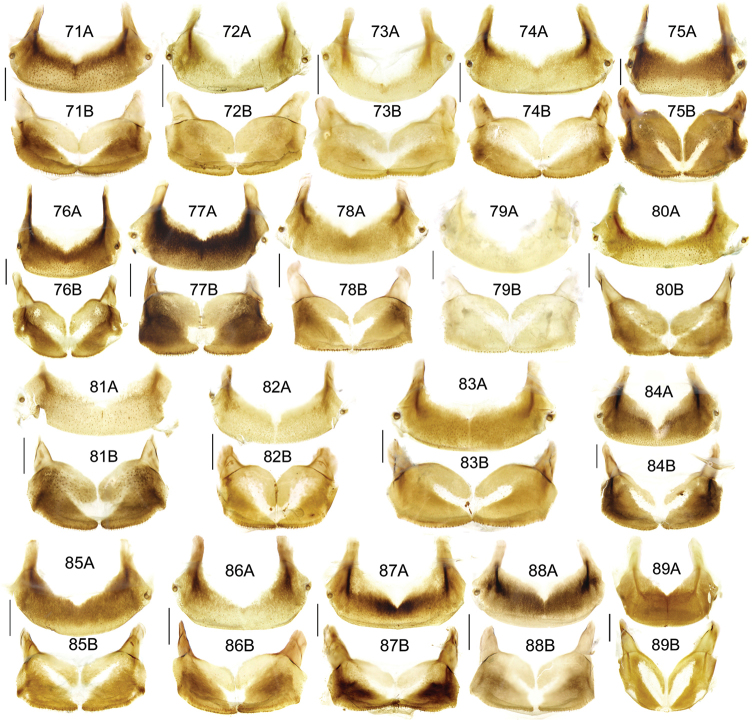
**A** female tergum VIII **B** female sternum VIII. **71** *Pterostichus adelphus* sp. n. **72** *Pterostichus cavazzutianus* s. str. replacement name **73** *Pterostichus cavazzutianus mianningensis* ssp. n. **74** *Pterostichus agilis* Allegro & Sciaky **75** *Pterostichus zhygealu* sp. n. **76** *Pterostichus camelus* sp. n. **77** *Pterostichus bullatus* Allegro & Sciaky **78** *Pterostichus baenningeri* Jedlička **79** *Pterostichus maitreya* sp. n. **80** *Pterostichus liciniformis* Csiki **81** *Pterostichus dimorphus* sp. n. **82** *Pterostichus wangjiani* sp. n. **83** *Pterostichus tumulus* sp. n. **84** *Pterostichus subtilissimus* Sciaky **85** *Pterostichus pohnerti* Jedlička **86** *Pterostichus xilingensis* Allegro & Sciaky **87** *Pterostichus beneshi* Sciaky **88** *Pterostichus zoiai* Sciaky **89** Pterostichus (Morphohaptoderus) schuelkei Sciaky & Wrase. Scale bar: 0.5 mm.

**Figures 90–101.**
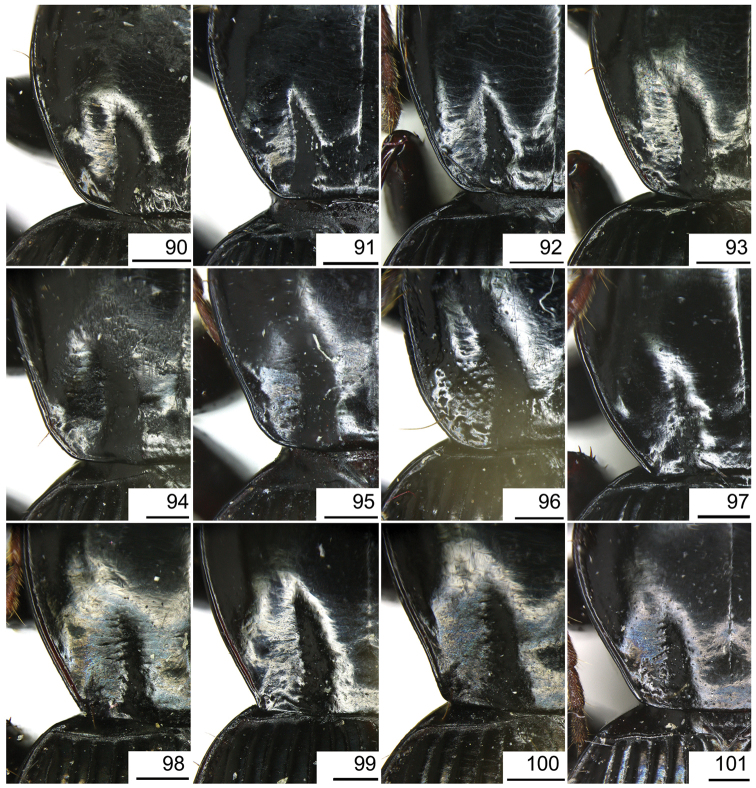
Pronotum hind angle and basal area of *Pterostichus (Circinatus)* spp. **90** *Pterostichus adelphus* sp. n. **91** *Pterostichus cavazzutianus* s. str. replacement name **92** *Pterostichus cavazzutianus mianningensis* ssp. n. **93** *Pterostichus agilis* Allegro & Sciaky **94** *Pterostichus zhygealu* sp. n. **95** *Pterostichus camelus* sp. n. **96** *Pterostichus bullatus* Allegro & Sciaky **97** *Pterostichus ailaoicus* sp. n. **98** *Pterostichus baenningeri* Jedlička **99** *Pterostichus maitreya* sp. n. **100** *Pterostichus miao* sp. n. **101** *Pterostichus yan* sp. n. Scale bar: 0.5 mm.

**Figures 102–113.**
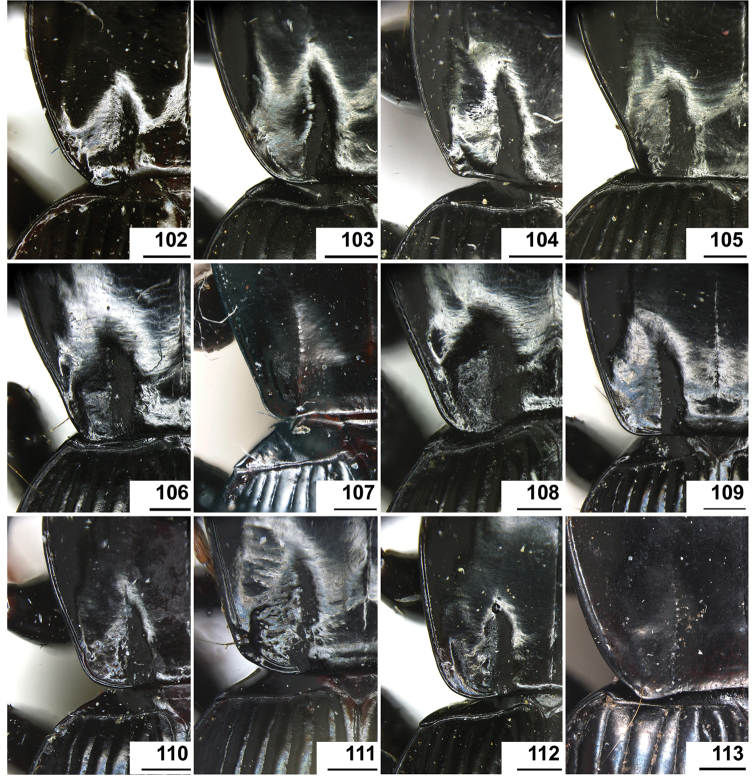
Pronotum hind angle and basal area of *Pterostichus (Circinatus)* spp. **102** *Pterostichus liciniformis* Csiki **103** *Pterostichus dimorphus* sp. n. **104** *Pterostichus wangjiani* sp. n. **105** *Pterostichus tumulus* sp. n. **106** *Pterostichus subtilissimus* Sciaky **107** *Pterostichus dentifer* Allegro & Sciaky **108** *Pterostichus yuxiaodongi* sp. n. **109** *Pterostichus beneshi* Sciaky **110** *Pterostichus zoiai* Sciaky **111** *Pterostichus pohnerti* Jedlička **112** *Pterostichus xilingensis* Allegro & Sciaky **113** Pterostichus (Morphohaptoderus) wenxianensis Allegro & Sciaky, holotype. Scale bar: 0.5 mm.

**Figures 114–125.**
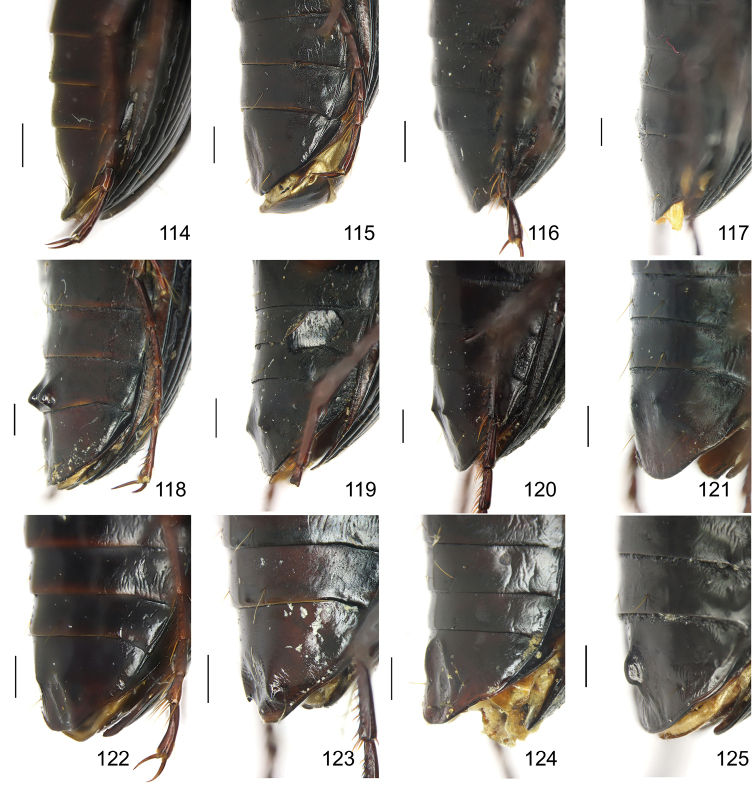
Male sternum of *Pterostichus (Circinatus)* spp. **114** *Pterostichus cavazzutianus* s. str. replacement name, locality: Yizi pass **115** *Pterostichus cavazzutianus mianningensis* ssp. n., paratype **116** *Pterostichus agilis* Allegro & Sciaky, locality: Luojishan **117** *Pterostichus zhygealu* sp. n., holotype **118** *Pterostichus camelus* sp. n., holotype **119** *Pterostichus baenningeri* Jedlička, locality: Jinfoshan **120** *Pterostichus maitreya* sp. n., holotype **121** *Pterostichus miao* sp. n., holotype **122** *Pterostichus liciniformis* Csiki, locality: Lijiang **123** *Pterostichus dimorphus* sp. n., holotype **124** *Pterostichus wangjiani* sp. n., holotype **125** *Pterostichus tumulus* sp. n., holotype. Scale bar: 0.5 mm.

**Figures 126–138.**
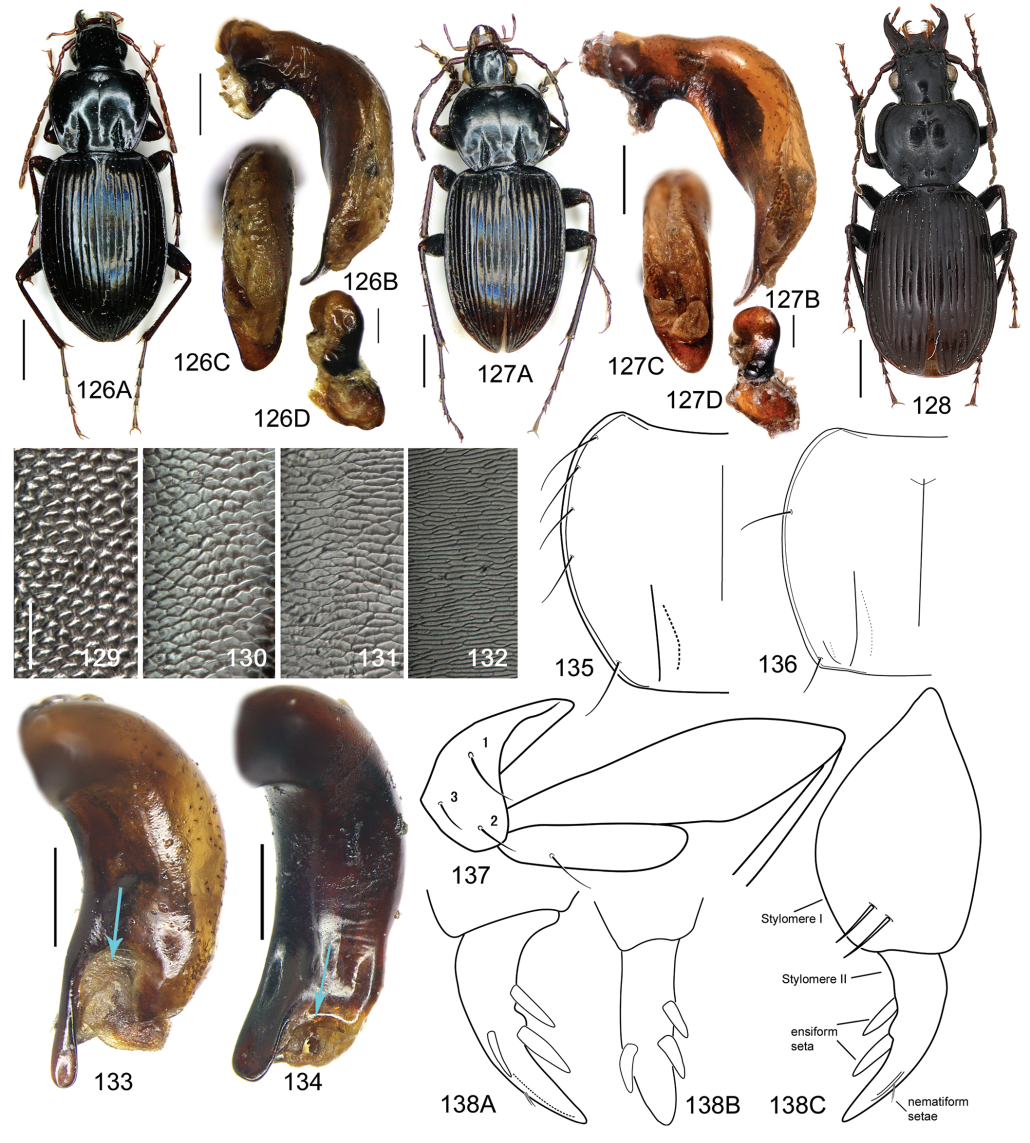
Characters of *Circinatus*. **126–127** Species removed from *Circinatus* **A** habitus **B** left lateral view of median lobe **C** dorsal view of median lobe **D** right paramere. **126** Pterostichus (Morphohaptoderus) schuelkei Sciaky & Wrase, holotype (**A**), locality: Houzhenzi **127** Pterostichus (Morphohaptoderus) wenxianensis Allegro & Sciaky, holotype **128** Habitus, an aberrant specimen of *Pterostichus bullatus* Allegro & Sciaky **129–132** Elytral microsculpture **129** granular microsculpture in female of *Pterostichus dimorphus* sp. n. **130** isodiametric microsculpture in male of *Pterostichus dimorphus* sp. n. **131** transversal microsculpture in *Pterostichus wangjiani* sp. n. **132** linear microsculpture in *Pterostichus maitreya* sp. n. **133–134** Ventral view of median lobe, showing ventral opening of apical orifice **133** *Pterostichus dimorphus* sp. n. **134** *Pterostichus wangjiani* sp. n. **135** Pronotum of *Pterostichus adelphus* sp. n. **136** pronotum of *Pterostichus wangjiani* sp. n. **137** Hind leg of Pterostichus (Morphohaptoderus) schuelkei Sciaky & Wrase, showing three setae on metacoxa **138** Stylomere of female ovipositor of *Pterostichus pohnerti* Jedlička, **A** dorsal view **B** outer lateral view **C** ventral view. Scale bars: 2.0 mm (**126A, 127A, 128**), 0.5 mm (**126B, 126C, 127B, 127C, 133, 134**), 0.2 mm (**126D, 127D**), 0.05 mm (**129–132**).

## Supplementary Material

XML Treatment for Circinatus

XML Treatment for Pterostichus (Circinatus) adelphus

XML Treatment for Pterostichus (Circinatus) ailaoicus

XML Treatment for Pterostichus (Circinatus) camelus

XML Treatment for Pterostichus (Circinatus) dimorphus

XML Treatment for Pterostichus (Circinatus) maitreya

XML Treatment for Pterostichus (Circinatus) cavazzutianus mianningensis

XML Treatment for Pterostichus (Circinatus) miao

XML Treatment for Pterostichus (Circinatus) tumulus

XML Treatment for Pterostichus (Circinatus) wangjiani

XML Treatment for Pterostichus (Circinatus) yan

XML Treatment for Pterostichus (Circinatus) yuxiaodongi

XML Treatment for Pterostichus (Circinatus) zhygealu

XML Treatment for Pterostichus (Circinatus) agilis

XML Treatment for Pterostichus (Circinatus) baenningeri

XML Treatment for Pterostichus (Circinatus) beneshi

XML Treatment for Pterostichus (Circinatus) bullatus

XML Treatment for Pterostichus (Circinatus) cavazzutianus s. str.

XML Treatment for Pterostichus (Circinatus) dentifer

XML Treatment for Pterostichus (Circinatus) liciniformis

XML Treatment for Pterostichus (Circinatus) pohnerti

XML Treatment for Pterostichus (Circinatus) subtilissimus

XML Treatment for Pterostichus (Circinatus) xilingensis

XML Treatment for Pterostichus (Circinatus) zoiai

XML Treatment for Pterostichus (Morphohaptoderus) schuelkei

XML Treatment for Pterostichus (Morphohaptoderus) wenxianensis

## Appendix

### Character coding

**External characters**

1. male elytral microsculpture: (0) transverse or isodiametric; (1) linear or absent.

2. pronotal posterior seta: (0) distant from hind angle; (1) close to hind angle.

3. pronotal hind angle: (0) completely rounded; (1) weakly angulate.

4. pronotal mid-lateral seta: (0) only one; (1) two or more.

5. pronotal outer basal foveal groove: (0) absent; (1) present.

6. pronotal base between outer and inner basal foveal grooves: (0) flat or convex; (1) depressed.

7. elytral shoulder angle (formed by basal ridge and lateral margin): (0) completely rounded; (1) forming obtuse angle.

8. elytral basal ridge: (0) approx horizontal; (1) strongly oblique lateral-posteriorly.

9. setae beneath fifth tarsomeres: (0) absent; (1) present.

10. outer ridge on second metatarsomere: (0) distinct, as on first metatarsomere; (1) shallow or absent, as on third metatarsomere.

**Male genital characters**

11. male penultimate sternum: (0) unmodified; (1) modified.

12. male terminal sternum: (0) modified; (1) unmodified.

13. male sexual character on terminal sternum: (0) longish ridge; (1) concave; (2) two tubercles.

14. median lobe of aedeagus apex (in dorsal view): (0) straight; (1) strongly bent to right.

15. apical lamella of aedeagus: (0) longer or subequal to its basal width; (1) shorter than half of its basal width.

16. apical lamella of aedeagus: (0) shorter than one fourth of apical orifice; (1) longer than one third of apical orifice.

17. apical lamella of aedeagus widened to apex: (0) no; (1) yes.

18. apical lamella base whether grooved on dorsal side: (0) not grooved; (1) grooved.

19. apical lamella base in dorsal view: (0) gradually narrowed after apical orifice; (1) abruptly narrowed after apical orifice.

20. apical lamella abruptly bent downwards before apex: (0) no; (1) yes.

21. apical orifice opened on ventral side: (0) no; (1) yes.

**Male endophallus characters**

22. major portion of endophallus located on: (0) ventral side of aedeagus; (1) ventral-apical or dorsal-apical side of aedeagus; (2) dorsal side of aedeagus.

23. scaled area on endophallus: (0) present; (1) absent.

24. endophallus basal lobe (adjacent to basal-ventral face of apical lamella): (0) absent; (1) present.

25. chitinized structure on endophallus venter: (0) absent; (1) present.

26. ventral lobe of endophallus divided into sub-lobes: (0) no; (1) yes.

27. right lobe on the right side of endophallus: (0) absent; (1) present.

28. left lobe on the left side of endophallus: (0) absent; (1) present.

29. left lobe of endophallus divided into sub-lobes or apex bifid: (0) yes; (1) no.

30. gonopore orientation: (0) to aedeagus base; (1) to aedeagus apex;.

**Female genital characters**

31. spermatheca receptaculum shape: (0) clavate or tubiform; (1) capitate.

32. ratio of spermatheca seminal canal length / receptaculum length: (0) 3 times or more; (1) approx 2 times.

33. basal sclerotized piece of seminal canal: (0) very small or absent; (1) well developed.

34. middle transparent region on female sternum VIII: (0) V-shaped, more than half width of sternum; (1) triangular or quadrate, less than half width of sternum.

35. anterior margin of female tergum VIII: (0) strongly notched in the middle; (1) not or very weakly notched in the middle.

36. ensiform setae at the inner margin of female stylomere II: (0) 0 or 1 seta; (1) 2 setae.

### Out-group material

Out-group 1: *Pterostichus (Gutta)* sp. cf. *gaoligongensis* Wrase & Schmidt: 2 males, 2 females, “CHINA, Yunnan Prov., Gongshan county, Dongshaofang-Yakou, N27°41'40", E98°28'47"; 3400 m, 2002.5.1, day, Liang H.B., Ba Weidong, Yuan Guodong, Li X.Q.”.

Out-group 2: Pterostichus (Sinosteropus) sinensis ssp.: 2 males, 3 females, “China, Sichuan prov., Kangding county, Jiagenba Xiang, Gonggashan Mt. W slope, pine forest, 4050 m, N29.77855, E101.69564; pit fall trap, 2012.VII.12, SHI Hongliang, LIU Ye & WANG Ya’nan lgt.”.

**Table:** **Characters matrix for subgenus *Circinatus* and two out-groups**

Character / Taxa	000000000111111111122222222222333333
	123456789012345678901234567890123456
*Gutta* (out-group)	0000000000000000000000100000–0000010
*Sinosteropus* (out-group)	1000000000000010000100000000–0000100
*Pterostichus adelphus*	100100100001–000000001011110–0101000
*Pterostichus cavazzutianus* s. str.	1001001000001000000001011110–0101110
*Pterostichus cavazzutianus mianningensis*	1001001000001000000001011110–0101010
*Pterostichus bullatus*	000101100001–001000001111110–0101101
*Pterostichus zhygealu*	000110100011–001000001011110–0101010
*Pterostichus agilis*	000110100011–001000001011110–0101011
*Pterostichus camelus*	000101101011–00100000?????????101010
*Pterostichus liciniformis*	000000000000100000001001101101000110
*Pterostichus dimorphus*	000010100100100010101000101101010110
*Pterostichus wangjiani*	011000100100100000111001101101001000
*Pterostichus baenningeri*	1110 00101000200001111001011111110110
*Pterostichus maitreya*	111000100000200011111001001110100100
*Pterostichus miao*	110000000000200010111???????????????
*Pterostichus yan*	110000100000200001111???????????????
*Pterostichus ailaoicus*	111000100100200110111???????????????
*Pterostichus tumulus*	001011100100000011111?????????011010
*Pterostichus subtilissimus*	000011100001–110000002100000–1101000
*Pterostichus yuxiaodongi*	000011110001–100100002000000–0??????
*Pterostichus dentifer*	000011110001–11000000???????????????
*Pterostichus beneshi*	100000100001–00000000200011110111100
*Pterostichus zoiai*	100000100001–01000000200011100111100
*Pterostichus xilingensis*	100010100001–001000001101000–1101110
*Pterostichus pohnerti*	100010100001–001000001011000–0101000

“?” = missing data; “–” = gap.
